# A novel prognostic model based on three integrin subunit genes-related signature for bladder cancer

**DOI:** 10.3389/fonc.2022.970576

**Published:** 2022-10-04

**Authors:** Hongtao Tu, Haolin Liu, Longfei Zhang, Zhiyong Tan, Hai Wang, Yongming Jiang, Zhongyou Xia, Liwei Guo, Xiaodong Xia, Peng Gu, Xiaodong Liu

**Affiliations:** ^1^ Department of Urology, The First Affiliated Hospital of Kunming Medical University, Kunming, China; ^2^ The First Affiliated Hospital of Kunming Medical University, Yunnan Province Clinical Research Center for Chronic Kidney Disease, Kunming, China; ^3^ Department of Urology, Institute of Urology, West China Hospital, Sichuan University, Chengdu, China; ^4^ Department of Vascular Surgery, The First Affiliated Hospital of Bengbu Medical College, Bengbu, China; ^5^ Department of Urology, The Second Affiliated Hospital of Kunming Medical University, Kunming, China; ^6^ Department of Urology, The Dazhu County People’s Hospital, Dazhou, China

**Keywords:** integrin subunit genes (ITGs), bladder cancer (BLCA), prognostic model, immune landscape, qRT-PCR

## Abstract

**Background:**

Presently, a comprehensive analysis of integrin subunit genes (ITGs) in bladder cancer (BLCA) is absent. This study endeavored to thoroughly analyze the utility of ITGs in BLCA through computer algorithm-based bioinformatics.

**Methods:**

BLCA-related materials were sourced from reputable databases, The Cancer Genome Atlas (TCGA) and Gene Expression Omnibus (GEO). R software-based bioinformatics analyses included limma-differential expression analysis, survival-Cox analysis, glmnet-Least absolute shrinkage and selection operator (LASSO), clusterProfiler-functional annotation, and gsva-estimate-immune landscape analysis. The expression difference of key genes was verified by quantitative real-time polymerase chain reaction (qRT-PCR).

**Results:**

Among the 11 ITGs that were abnormally expressed in BLCA, ITGA7, ITGA5, and ITGB6 were categorized as the optimal variables for structuring the risk model. The high-risk subcategories were typified by brief survival, abysmal prognosis, prominent immune and stromal markers, and depressed tumor purity. The risk model was also an isolated indicator of the impact of clinical outcomes in BLCA patients. Moreover, the risk model, specifically the high-risk subcategory with inferior prognosis, became heavily interlinked with the immune-inflammatory response and smooth muscle contraction and relaxation.

**Conclusion:**

This study determined three ITGs with prognostic values (ITGA7, ITGA5, and ITGB6), composed a novel (ITG-associated) prognostic gene signature, and preliminarily probed the latent molecular mechanisms of the model.

## Introduction

Bladder cancer (BLCA) originates from the mucosal epithelium of the bladder and is one of the most common malignancies of the genitourinary system. Globally, the prevalence of BLCA ranks ninth among malignant tumors, with about 500,000 new cases and 130,000 deaths from BLCA each year, and the tendency is gradually rising annually (1). Clinically, the prognosis of patients with BLCA is closely related to the degree of infiltration, depth of invasion and metastatic potential of the lesion. BLCA is classified into non-muscle invasive bladder cancer (NMIBC) and muscle invasive bladder cancer (MIBC) according to the degree of tumor infiltration (2). About 60% of bladder cancers are NMIBC at the initial diagnosis and have a better prognosis. Approximately 50%-70% of NMIBC still recur and may develop into MIBC or even distant metastases after treatment, and the 5-year overall survival rate for metastatic MIBC is only 6% (3). Despite tremendous advances in imaging, chemotherapy and surgery, there has been no significant change in clinical survival benefits. In recent years, our understanding of the molecular pathogenesis of BLCA has improved dramatically with the rapid advances in gene sequencing technology; however, the number of known biomarkers associated with BLCA prognosis remains limited (4). Therefore, exploring and studying biomarkers that can predict and monitor the development of BLCA is crucial for the diagnosis, precise treatment and improved prognosis of BLCA patients.

Integrins are a group of heterophilic cell adhesion molecules commonly associated with vertebrate cell surfaces, mediating cell-to-cell and cell-to-extracellular matrix mutual recognition and adhesion, and having a role in linking intra and extracellular structures (5, 6). The primary function of integrins is to provide position control of the actions of cytokine and growth factor receptors to coordinate development, regeneration and various repair processes, and also act as signaling receptors that can control intracellular pathways that regulate cell survival, proliferation and cell fate (7, 8). For example, integrins and receptor tyrosine kinases (RTKs) are jointly involved to ensure optimal activation of pro-native and pro-survival signals *via* the Ras-extracellular signal-regulated kinase (ERK) and phosphatidylinositol 3-kinase (PI3K)-AKT signaling pathways. Integrins and growth factor receptors co-activate critical downstream signaling components such as Shc, PI3K, Rac and MEK in a summated, co-dependent or synergistic manner, and optimal activation of many downstream targets such as AP-1 (cJun/c-Fos) and target of rapamycin (TOR) requires the simultaneous linkage of integrins and RTK (9). Integrin signaling drives a variety of stem cell functions including tumor inception, epithelial plasticity, metastatic reactivation and resistance to oncogenes and immune-targeted therapies (10). A few integrin subunit genes (ITGs) have also been reported to be associated with epigenetic alterations in BLCA, but the underlying mechanisms of ITGs remain unclear. Based on the above, integrin-related pathways could be potential targets for bladder cancer treatment and may be of targeted therapeutic value in the future. However, few studies have been reported on the clinical prognosis and biological course of ITG in BLCA. To date, there are no relevant reports in the literature on comprehensive analysis of ITGs in BLCA. Therefore, this study aimed to explore the expression of ITGs in BLCA based on publicly available high-throughput sequencing data, and to reveal their biological processes and signaling through bioinformatics approaches, and to elucidate the potential prognostic value of ITGs in BLCA.

In this study, we aimed to thoroughly investigate the role of ITGs in BLCA and develop a novel survival risk stratification model based on ITGs signature. First, BLCA transcriptome data were downloaded from TCGA to comprehensively analyze the expression profile of ITGs and their prognostic value in BLCA prognosis. Subsequently, ITGs signature was created in the TCGA cohort and then validated in the GEO cohort. Finally, we also analyzed the association of the ITGs signature with the immune microenvironment of BLCA. We hope that our findings will provide a more comprehensive understanding of the role of ITGs in BLCA.

## Materials and methods

### Data capture

The 19 normal and 414 tumor samples selected for inclusion in this study were from the TCGA-BLCA cohort, of which, 383 BLCA samples contained complete survival time records. Both the GSE32894 and GSE7476 sets were extracted from the GEO database. The GSE32894 dataset (https://www.ncbi.nlm.nih.gov/geo/query/acc.cgi?acc=GSE32894) (11), which was focused on the risk model validation, contained 224 available BLCA samples (which could be queried for complete survival information and matched expression profiles). The GSE7476 dataset (https://www.ncbi.nlm.nih.gov/geo/query/acc.cgi?acc=GSE7476) (12), which embraced 3 healthy bladder tissues (controls) and 7 BLCA tumor tissues, was mainly responsible for the authentication of the prognostic genes’ expression patterns.

### Variance expression analysis

Gene expression abnormality analysis was implemented in the R software using the limma package. The database for the analysis was the mRNA expression profiles of normal and BLCA samples from the TCGA database. Saliency thresholds: |log_2_ fold change (FC)| > 0.5 and adjusted (adj.) *P*< 0.05.

### Differentially expressed ITGs (DE-ITGs)

Thirty ITGs were retrieved from the reviewed published literature (13) (**Table 1**). ITGs belonging to differentially expressed genes (DEGs), designated as DE-ITGs, were recognized in Jvenn online tool (14) using intersection analysis.

**Table 1:** Thirty ITGs.

ITG names					
NO.	Name	NO.	Name	NO.	Name
1	ITGA1	11	ITGA8	21	ITGB1BP2
2	ITGA10	12	ITGA9	22	ITGB2
3	ITGA11	13	ITGAD	23	ITGB3
4	ITGA2	14	ITGAE	24	ITGB3BP
5	ITGA2B	15	ITGAL	25	ITGB4
6	ITGA3	16	ITGAM	26	ITGB5
7	ITGA4	17	ITGAV	27	ITGB6
8	ITGA5	18	ITGAX	28	ITGB7
9	ITGA6	19	ITGB1	29	ITGB8
10	ITGA7	20	ITGB1BP1	30	ITGBL1

### Connectivity networks for DE-ITGs

The physical and functional linkages of DE-ITGs were evaluated using the Search Tool for the Retrieval of Interacting Genes (STRING) database (URL: http://string-db.org) (15). Cytoscape software (16) was in turn deployed to imagine the PPI network of DE-ITGs.

### Regression analysis and risk scoring system

The 383 TCGA-BLCA samples were sorted by randomization (ratio 7:3) into a training set (n = 269) and a test set (n = 114). The optimal DE-ITGs were appraised by a combination of Cox (univariate) and LASSO (glmnet package in the R) analyses (in the training set). Variables with Cox *P*< 0.05 were incorporated into the LASSO procedure, and the corresponding variables were retrieved subject to the minimum value of Lambda (λ). Risk scores for the BLCA samples in each dataset were captured by the regression coefficients (coef; **Table 2**) and expressions of the selected ITGs based on the following formula:

**Table 2:** The regression coefficients of three gene characterastics calculated by LASSO regression algorithm.

Gene	Coefficent
ITGA7	0.08440408
ITGA5	0.0920655
ITGB6	-0.10878208


$$risk\;score=coef_{1}\times expression\;of\;gene_{1}+coef_{2}\times expression\;of\;gene_{2}+\cdots coef_{n}\times expression\;of\;gene_{n}$$

The optimal threshold for separating patients into high-risk and low-risk subgroups (based on risk scores in the corresponding dataset) was calculated using the surv_cutpoint function, which is affiliated with the R package survminer. The competence of the risk model to distinguish and forecast patient clinical endpoints was scrutinized using the R package survival and survivalROC.

The independence of the model affecting the overall survival of BLCA patients was furthermore inferred by Cox analysis (R package), univariate and multivariate regression, and other considered parameters including baseline (age and sex) and clinical characteristics (grade, stage, and TNM stage) of the sample.

### Enrichment analysis of pre-defined gene sets based on the risk model

The Gene Set Enrichment Analysis (GSEA) was implemented using the GSEA subfunction from the R software clusterProfiler package (17). Specifically, we first calculated log_2_ FC values for all genes between each risk subsection by the R package limma, and then set the Gene Ontology (GO) and Kyoto Encyclopedia of Genes and Genomes (KEGG) gene sets in the clusterProfiler package as the indicator gene sets, and the gene sets that satisfied the |normalized enrichment scores (NES)| > 1, *P*< 0.05, and q< 0.05 as significantly enriched.

### Inference of immune cell abundance based on the risk model

The level and activity of 28 immune gene sets in BLCA patients were appraised by the single-sample gene set enrichment analysis (ssGSEA) (18) function of R package GSVA (19). The extent of immune cell infiltration (immune score), stromal cell content (stromal score), ESTIMATE score (combined immune and stromal markers), and tumor purity were also measured for apiece BLCA sample utilizing ESTIMATE (20) (R package estimate).

### Patient preparation

Six pairs of BLCA tissues and adjacent normal specimens were collected from the First Affiliated Hospital of Kunming Medical University. All participants provided written informed consent prior to the study. The experiment was approved by the Institutional Ethics Committee of the First Affiliated Hospital of Kunming Medical University. All BLCA patients did not receive any treatment prior to surgery. Finally, the tissues were frozen in liquid nitrogen and then stored in a -80°C refrigerator pending further experiments.

### RNA isolation and quantitative real-time polymerase chain reaction (qRT-PCR)

A total of six pairs of BLCA and paracancerous tissue samples were lysed with TRIzol reagent (Life Technologies-Invitrogen, Carlsbad, CA, USA), and total RNA was isolated following the manufacturer’s instructions. Then the concentration and purity of the RNA solution were quantified using a NanoDrop 2000FC-3100 nucleic acid protein quantifier (Thermo Fisher Scientific, Waltham, MA, USALife Real). The extracted RNA was reverse-transcribed to cDNA using the sweScript RT I First strand cDNA SynthesisAll-in-OneTM First-Strand cDNA Synthesis Kit (Servicebio, Wuhan, China) prior to qRT-PCR. The qRT-PCR reaction consisted of 3 µl of reverse transcription product, 5 µl of 5×BlazeTaq qPCR Mix (Genecopoeia, Guangzhou, China), and 1 µl each of forward and reverse primer. PCR was performed in a BIO-RAD CFX96 Touch TM PCR detection system (Bio-Rad Laboratories, Inc., USA) under the following conditions: initial denaturation at 95°C for 1 min, followed by 40 cycles that each involved incubation at 95°C for 20 s, 55°C for 20 s, and 72°C for 30 s. The forward primer of ITGA5 was “CAGAAGCAGAAGGGAGGGGTA”. The reverse primer of ITGA5 was “CGATGTGAATCGGCGAGAGTT”. The forward primer of ITGA7 was “CTCTTCGCTTGCCCGTTG”. The reverse primer of ITGA7 was “CTCGCTGCCTTGCCTCAT”. The forward primer of ITGB6 was “TGGTTCTGTTTCCTGCTCTCTG”. The reverse primer of ITGB6 was “CCACTTGGCTTTTGATCGTTCT”. The forward primer of GAPDH was “GGAAGGTGAAGGTCGGAGT”. The reverse primer of GAPDH was “TGAGGTCAATGAAGGGGTC”. All primers were synthesized by Servicebio (Servicebio, Wuhan, China). The GAPDH gene served as an internal control, and the relative expression of 3 key mRNAs was determined using the 2^-ΔΔCt^ method (21). The experiment was repeated in triplicate on independent occasions. Statistical differences of 3 key mRNAs between normal and BLCA samples were detected by unpaired t-tests, using GraphPad Prism V6 (GraphPad Software, La Jolla, CA, USA), and the level of statistical significance was tested and represented as *** for *P*< 0.001 and **** for *P*< 0.0001.

### Statistical analysis

All statistical analyses were executed in the corresponding R software (Version 4.0.3) as described. If not otherwise stated,*P*< 0.05 represents the optimal screening threshold.

## Results

### Analysis of BLCA-related DE-ITGs

**Figure 1:**
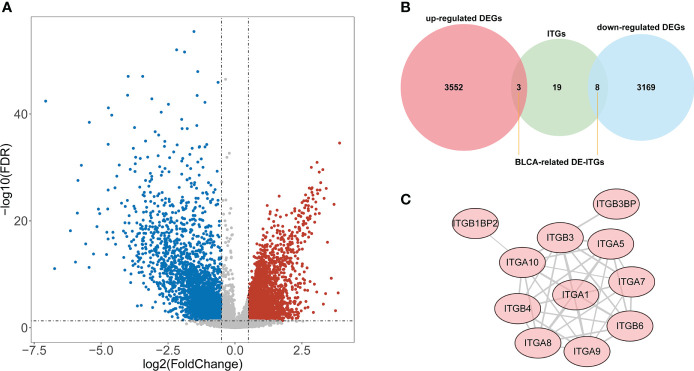
Identification of BLCA-related DE-ITGs. **(A)** 6732 DEGs were identified between BLCA and normal groups from the TCGA cohort . **(B)** 11 ITGs were illustrated from DEGs by the Venn diagram. **(C)** PPI network of DE-ITGs.

According to the TCGA cohort, a total of 6732 DEGs were recognized between BLCA and normal groups after differential expression analysis. There were 3555 genes that met log_2_ FC > 0.5 and adj. *P*< 0.05, and their expression was significantly upregulated in BLCA samples; 3177 genes that were downregulated and fulfilled log_2_ FC< -0.5 and adj. *P*< 0.05 (**Figure 1A**). Among these DEGs, 11 genes were identified as ITGs as illustrated in **Figure 1B**. Among them, ITGB3BP, ITGB4, and ITGB6 were up-regulated in BLCA; while ITGA1, ITGA10, ITGA5, ITGA7, ITGA8, ITGA9, ITGB1BP2, and ITGB3 were down-regulated genes; they were defined as DE-ITGs. **Figure 1C** showed the reciprocal relationship of these DE-ITGs.

### Risk characteristics associated with ITGs

**Figure 2:**
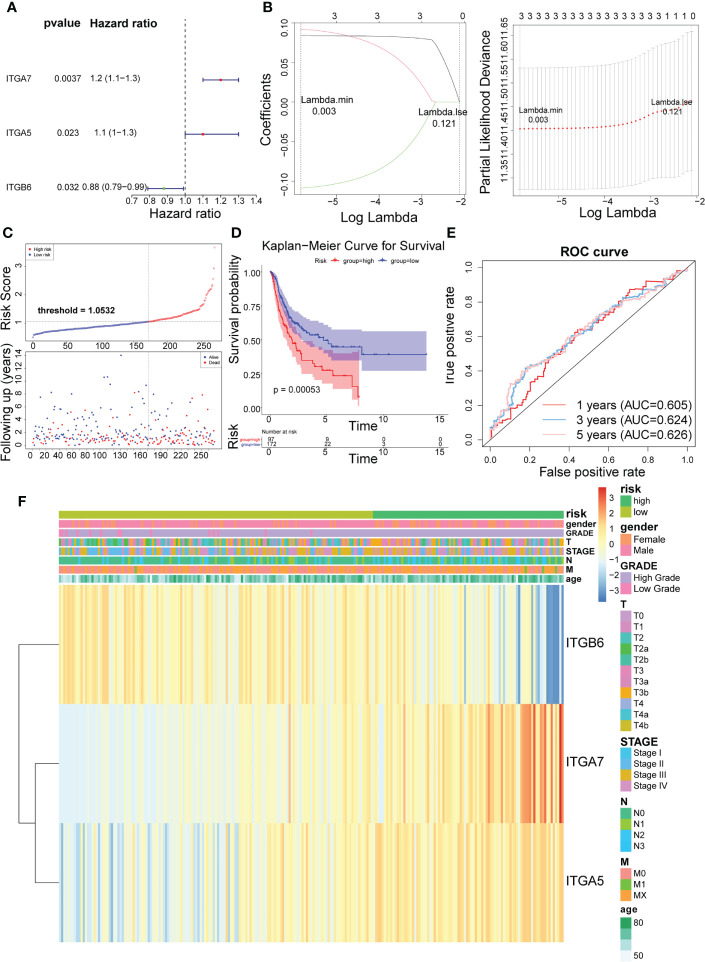
Identification of 3 risk characteristics associated with ITGs in the training set from TCGA cohort. **(A)** Three condidate model genes were screened by univariate Cox analysis. **(B)** Three risk characteristics associated with ITGs were identified by LASSO algorithm. **(C)** Risk score of the three risk characteristics. **(D)** Kaplan-Meier curve of the three risk characteristics. **(E)** ROC curve of the three risk characteristics. **(F)** The heatmap of the three risk characterastics in high- and low-risk groups, the distribution of clinicopathological features was compared between the low- and high-risk groups.

In the training set, Cox analysis (univariate) indicated that ITGA7 (*P* = 0.0037), ITGA5 (*P* = 0.023), and ITGB6 (*P* = 0.032) with *P*< 0.05 were the candidate model genes (**Figure 2A**). After further feature dimensionality reduction analysis, the LASSO algorithm identified ITGA7, ITGA5, and ITGB6 as the optimal ITGs for the construction of prognostic signature based on λ _min_ = 0.003 (**Figure 2B**).

Risk scores for the training set-BLCA patients were calculated as previously described and the specimens were classified into high- and low-risk subgroups according to the cutoff value = 1.0532 (**Figure 2C**). The position of the red curve representing the high-risk score was appreciably below than the curve for the low-risk subtype (blue) (*P* = 0.00053; **Figure 2D**). Predictive sensitivity analysis indicated that the risk model had tolerable prognostic validity in the training set (**Figure 2E**). Moreover, ITGA5 and ITGA7 were found to be more inclined to be expressed in the high-risk group; ITGB6, on the contrary (**Figure 2F**).

**Figure 3:**
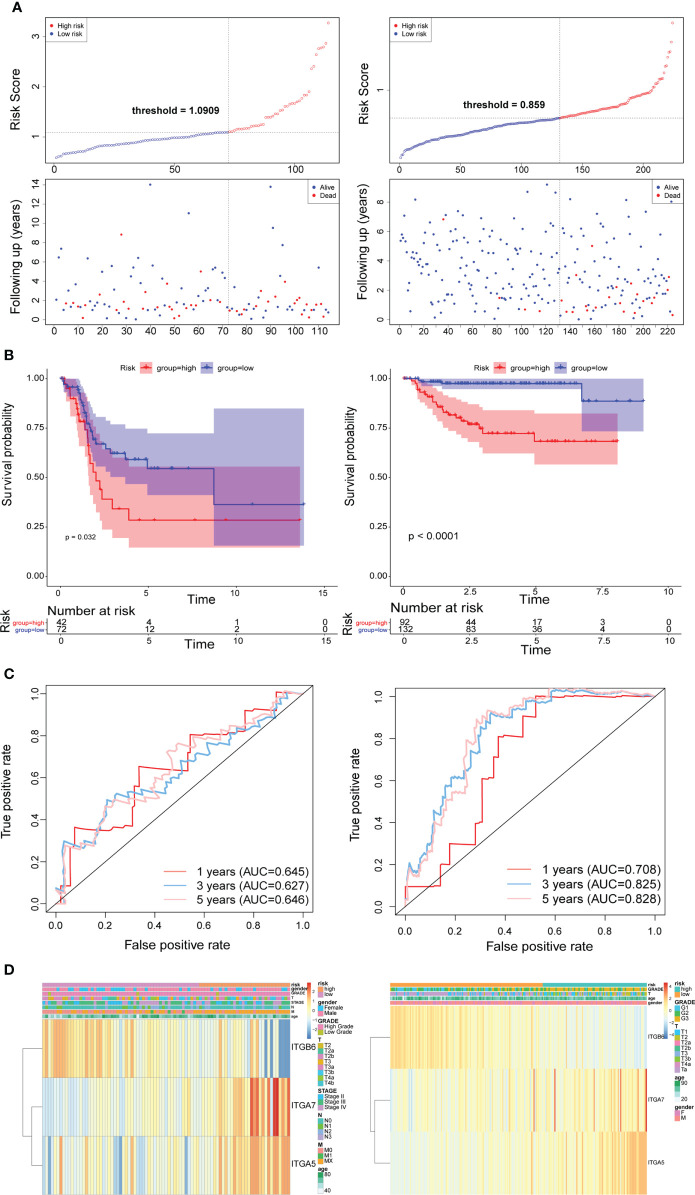
Time-dependent ROC analysis, risk score analysis, and Kaplan-Meier analysis for the three characteristics in testing set from TCGA (left) and the validation set from GSE32894 cohort (right). **(A)** Risk score of three gene signature. **(B)** Kaplan-Meier curve of the three risk characteratics. **(C)** ROC curve of the three-gene signature. **(D)** The heatmap of the three gene characterastics in high- and low-risk groups, the distribution of clinicopathological features was compared between the low- and high-risk groups.

Subsequently, we implemented equal analysis in the testing set and the GSE32894 cohort. The rendering of the risk scoring system behaved exactly as in the training set (**Figure 3A**). The height of the blue-low risk scoring curve outweighed the red curve (high-risk subcategory) (**Figure 3B**). The predictive strength of the ITGs model was more impressive in the GSE32894 cohort (**Figure 3C**). Additionally, the relationships across the three prognostic ITGs with risk score subcategories (in both validation cohorts) were illustrated in **Figure 3D**.

### ITGs-based risk model as an individual predictor of outcome in BLCA patients

**Figure 4:**
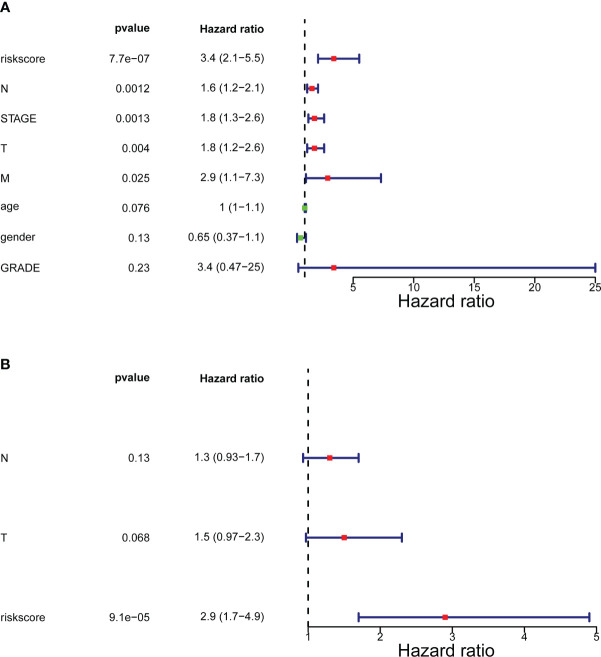
Forrest plot of the univariate and multivariate Cox regression analysis. **(A)** Engaged clinical characteristics into univariate Cox regressive. **(B)** Multivariate Cox regressive. The green square indicates that the HR value is less than 1, the red square indicates that the HR alue is larger than 1, and the line segments on both sides of the square are the 95% confidence interval of the HR Value.

In the training set **(Supplementary Table 1)**, the testing set **(Supplementary Table 2)**, and the GSE32894 cohort **(Supplementary Table 3)**, ANOVAs suggested that the distribution of clinical characteristics of patients in distinct risk subcategories was strikingly diverse. The Cox analyses (univariate and multivariate) pointed to the risk score as the only stand-alone prognostic predictor for patients with BLCA (both *P*< 0.05; **Figure 4**).

### Uncovering the molecular mechanisms involved in the risk score

**Figure 5:**
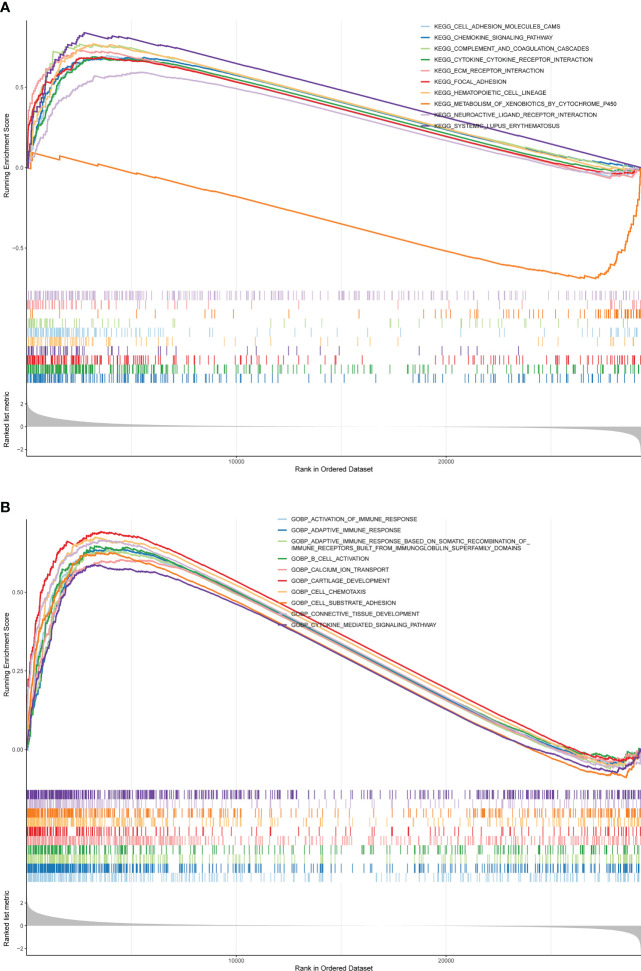
GSEA is adopted to annotate the genes with different expression in the terms of GO and KEGG between different risk groups. **(A)** Top 10 KEGG pathways. **(B)** Top 10 GO pathways.

GSEA was conducted to analyze the enrichment differences in the terms of GO and KEGG between different risk groups. A total of 41 pathways were activated in the high-risk group, mainly related to immune-inflammatory responses (‘chemokine signaling pathway’, ‘primary immunodeficiency’, ‘antigen processing and presentation’, *etc.*) and multiple cancer (‘systemic lupus erythematosus’, ‘dilated cardiomyopathy’, ‘pathways in cancer’, *etc.*) (**Figure 5A**; **Supplementary Table 4**). In the GO annotation system, a total of 1776 terms were harvested (**Supplementary Table 5**), where the high-risk subset was tightly matched to immune response (‘activation of immune response’, ‘adaptive immune response’, ‘regulation of immune effector process’, *etc.*), immune cell physiological processes (‘granulocytes migration’, ‘leukocyte proliferation’, ‘mononuclear cell differentiation’, *etc.*), tissue and organ development (‘muscle organ development’, ‘regulation of vasculature development’, ‘bone development’, *etc.*), and multiple diseases (‘aortic aneurysm’, ‘meningitis’, ‘vasculitis’, *etc.*); notably, smooth muscle (regulation of contraction and relaxation) and smooth muscle cell proliferation, migration, and differentiation terms were prominently enrolled in the high-risk subtype. **Figure 5B** exhibited the top 10 terms in the GO system. This evidence suggested that the risk score may influence disease progression and clinical outcomes in BLCA patients by modulating cancer trigger pathways, smooth muscle pathways, and immune response pathways.

### ITGs-based high scoring group with robust immune cell infiltration characteristics

**Figure 6:**
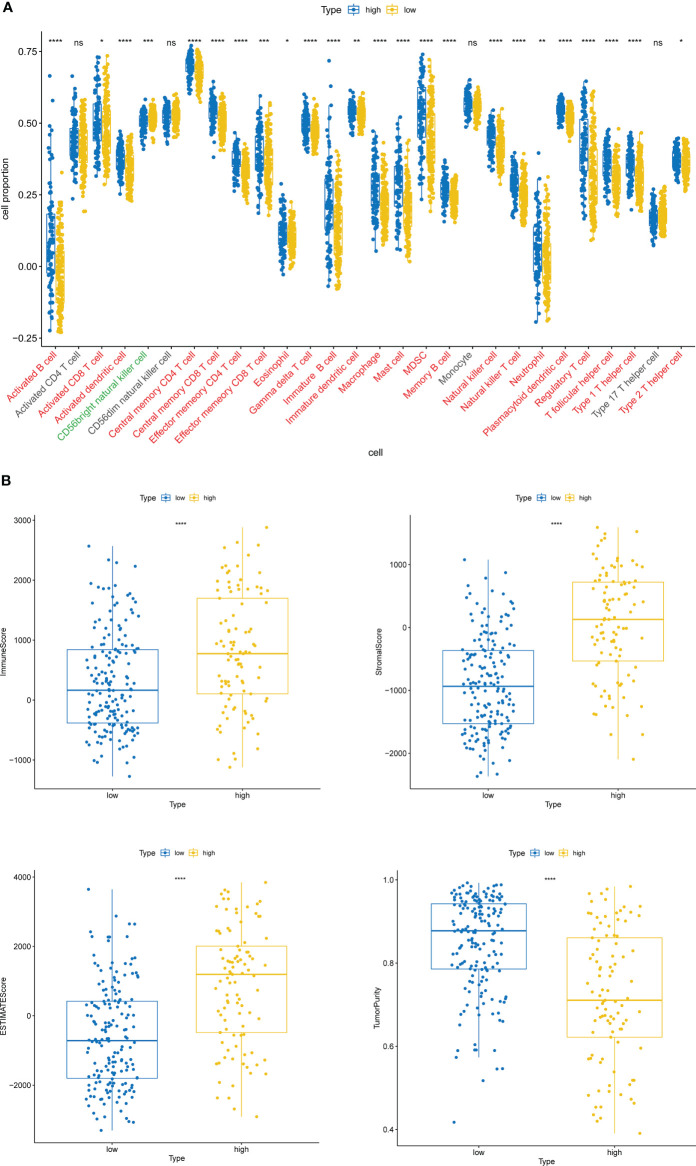
Immune cell infiltration characteristics of high- and low-risk groups. **(A)** The content of 28 immune cells in diverse risk subclasses assessed by the ssGSEA algorithm. ns, non-significant. **(B)** The immune score, stromal score, ESTIMATE score, and tumor purity in diverse risk subgroups assessed by the ESTIMATE algorithm. *P < 0.05, **P < 0.01, ***P < 0.001,****P < 0.0001.

Inspired by the above results, we extrapolated the content of 28 immune cells in diverse risk subclasses *via* the ssGSEA algorithm. Except for Activated CD4 T cell, CD56dim natural killer cell, Monocyte, and Type 17 T helper cell, which were comparable in both risk subcategories (all rank-sum test *P* > 0.05), all the other 24 immune cells were strikingly divergent across the above two categories of samples (all rank-sum test *P*< 0.05); only CD56bright natural killer cell was detected to be more infiltrative in the low-risk subgroup (rank-sum test *P*< 0.05) (**Figure 6A**). Meanwhile, the ESTIMATE algorithm demonstrated that high-risk subgroup patients had more immune and stromal cells and reduced tumor purity (**Figure 6B**).

### Detection of mRNA expression levels of prognostic genes in BLCA clinical samples

**Figure 7:**
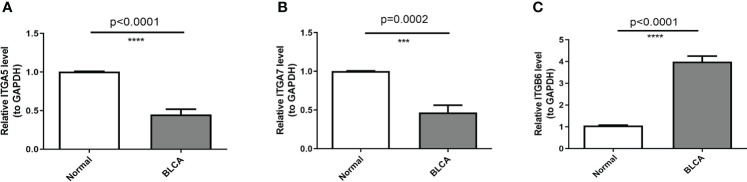
Exprerimental validation of ITGA5, ITGA7, and ITGB6. **(A)** Relative mRNA expression of ITGA5 in BLAC tissue and paracancerous tissues. **(B)** The mRNA expression level of ITGA7 in mRNA expression levels of prognostic genes in BLCA clinical samples. **(C)**The mRNA expression level of ITGB6 in mRNA expression levels of prognostic genes in BLCA clinical samples ***P < 0.001,****P < 0.0001.

We matched the expression profiles of three prognostic genes in the TCGA-BLCA (**Supplementary Figure 1A**) and GSE7476 (**Supplementary Figure 1B**) datasets and found that both ITGA5 and ITGA7 were significantly reduced in tumor samples; while ITGB6 was notably overexpressed in the BLCA group (all *P*< 0.05). Furthermore, a total of 6 samples were collected from newly diagnosed BLCA patients in The First Affiliated Hospital of Kunming Medical University from March 2022 to May 2022. ITGA5 (*P*< 0.0001) and ITGA7 (*P* = 0.0002) were significantly reduced in the BLCA population; whereas ITGB6 (*P*< 0.0001) was markedly overexpressed in the BLCA group (**Figure 7**), which in accordance with bioinformatics results.

## Discussion

BLCA is a multi-step, multifactorial and heterogeneous disease with a high disease burden and a poor prognosis in the event of metastasis and recurrence (1, 2). ITGs are a widely known class of cell adhesion molecule receptors that have been proven to be involved in cancer progression including pancreatic, colorectal, gastric and breast cancers (13, 22). Up until now, there has been no study to explore the role of ITGs in BLCA and their predictive value for clinical prognosis. In this study, machine learning algorithms (univariate Cox and LASSO) were used to identify the prognostic signatures associated with ITGs consisting of ITGA5, ITGB6 and ITGA7.

In our risk stratification model, high expression of ITGA5 and ITGA7 was associated with poorer survival, and in contrast to ITGB6. It has been shown that overexpression of ITGA5 is closely associated with enhanced O-GlcNAcylation, accelerating the progression of colorectal cancer. ITGA5 promotes proliferation, migration and invasion of oral squamous cell carcinoma cell lines through EMT (epithelial-mesenchymal transition) (23). ITGA5 plays an important role in Ta- T2 and T1-T2 transitions (24), suggesting a correlation between increased ITGA5 expression and histological staging, and a negative correlation between ITGA5 upregulation and prognostic overall survival in BLCA. Silencing ITGB6 inhibits the proliferation, migration and invasion of cervical cancer cells and promotes apoptosis by inhibiting the JAK/STAT signaling pathway (25). Low expression of ITGB6 in cholangiocarcinoma is associated with poorer prognosis and increased invasiveness (26). In the model we studied, ITGB6 was highly expressed in a low-risk population, possibly early in tumourigenesis, enhancing tumor cell adhesion and the ECM barrier, and may act as a protective factor in the risk stratification of BLCA mortality. ITGA7 acts as a pro-oncogene, promoting the stemness of oral squamous cell carcinoma cells and subsequently inducing tumourigenicity and metastasis (27). ITGA7 is highly expressed in hepatocellular carcinoma cells and knockdown of ITGA7 inhibits proliferation, migration, invasion and EMT of hepatocellular carcinoma cells (28). Due to the complexity of malignant pathological processes, different malignancies may be different in their pathological features and the effect of ITGA7 on cellular function and its potential mechanisms in different cancers may differ. We found that high expression of ITGA7 in high-risk groups may confer a worse survival benefit to patients, and there are few studies on ITGA7 associated with BLCA, and more research is needed.

Interestingly, we found that the expression levels of both ITGA5 and ITGA7 were down-regulated in BLCA compared to normal samples, while high expression was associated with poor OS (**Figures 7**, **2**). Conversely, ITGB6 was upregulated in BLCA, while low expression of ITGB6 was associated with poor OS (**Figures 7**, **2**). Previous studies have shown that CXCL11 expression is significantly upregulated in colon adenocarcinoma and that upregulation of CXCL11 expression is associated with better prognosis, and it has been speculated that the contradiction between CXCL11 expression and prognosis may be due to the complexity of regulation (29). Herewith, we hypothesize that ITGA5, ITGB6 and ITGA7 are changing dynamically in influencing the onset and development.

In addition, GSEA analysis revealed that immune inflammatory responses and multiple cancers (**Figure 5**), among other KEGG pathways, differ significantly between high- and low-risk groups. It has been shown that the presence of a large number of immune/inflammatory cells and cytokines in the tumor microenvironment leads to a chronic inflammatory state and immune suppression, regulating tumor cell migration, invasion, metastasis and anticancer drug sensitivity (30). Studies have confirmed that SLE is associated with an overall increased risk of cancer compared to the normal population and is a risk factor for cancer (31). This suggests that patients with BLCA in the high-risk group may have an impact on survival time due to dysregulation of immune inflammatory response pathways and multiple cancer pathways, among others. In the GO annotation system, the high-risk group is closely matched to immune responses, immune cell physiological processes, tissue and organ development and multiple diseases (32). Notably, smooth muscle (regulation of contraction and relaxation) and smooth muscle cell proliferation, migration and differentiation conditions are significantly involved in the high-risk subtype. Idiopathic urinary incontinence is a common complication of BLCA. Studies have shown that BLCA is associated with dramatic changes in the contractility of the smooth muscle of the detrusor (33, 34). One of the features of cancers that occur in the bladder is that the tumor invades and crosses the biophysical barrier of the smooth muscle (35). Based on the above literature, we hypothesise that modulation of cancer trigger pathways, smooth muscle pathways and immune response pathways contribute to the differences in prognosis between high and low risk patients and influence disease progression and clinical outcomes in patients with BLCA.

The high-risk group based on ITGs had a stronger immune cell infiltration profile with significantly higher immune scores, stromal scores and ESTIMATE scores than the low-risk group, while the opposite was true for tumor purity (**Figure 6**). The microenvironment of bladder tumor tissue contains not only tumor cells, but also stromal cells and immune cells, and others. Immune cells are an important component of the tumor stroma and cross-talk between cancer cells and proximal immune cells ultimately results in an environment that promotes tumor growth and metastasis (36). The predictive value of immune cells has been extensively studied. Stromal cell scores were positively correlated with cancer staging, indicating that the stromal component of TME may play an important role in BLCA progression (37). According to our findings, only CD56bright natural killer cells were detected to be more infiltrative in the low-risk subgroup (**Figure 6B**). It has been shown that CD56bright NK cells produce a large number of immunomodulatory cytokines and chemokines, exerting more immunomodulatory effects, and CD56bright NK cells have also recently been shown to be specifically responsive and protective against Epstein-Barr virus (EBV) infection in secondary lymphoid tissue (38). Thus, high levels of CD56bright natural killer cells may be identified as a protective factor against BLCA and are associated with good survival outcomes. The high-risk group was enriched with a high number of immune and stromal cells, diluting the purity of tumor cells and resulting in lower tumor purity in the high risk group, while the opposite was true in the low risk group. Patients with low tumor cell purity rarely show a good prognostic impact, but are more likely to be classified as malignant entities and to have a shorter survival time. On the one hand, tumor cells with limited proliferation and invasiveness tend to grow slowly, forming solid masses with less infiltration of non-tumor cells. On the other hand, the presence of tumor cells, capable of dominating the microenvironment, recruits a large number of surrounding cells and causes them to succumb, constituting a protective shield (39). Thus, high ESTIMATE scores, low tumor purity and associated cellular heterogeneity may account for the poor prognosis of invasive tumors.

Although some prognostic models for predicting BLCA have been developed in previous studies, our study has several advantages over them. Firstly, we used the new algorithm LASSO regression analysis as a screening variable to build a prognostic model, which was able to adjust for overfitting of the model, thereby avoiding extreme predictions and significantly improving prediction accuracy. Secondly, the model was able to demonstrate good performance in discrimination and calibration through internal and external validation. Clinicians may benefit from combining our model with other models. Relatively speaking, this study also has drawbacks. Firstly this is a retrospective analysis and selection bias may occur. Secondly, the endpoint of this study was OS and we did not assess the applicability of the model for predicting disease-free survival (DFS), distant metastasis-free survival (DMFS) and locoregional recurrence-free survival (LRFS) in patients with BLCA. It may be better to combine OS with DFS and DMFS. Finally, although we performed a multi-faceted, multi-database validation, the amount of data in this study was relatively small and the analysis may be biased. Therefore, future large-scale and multi-center validation of the model is needed.

In conclusion, we identified three ITGs (ITGA7, ITGA5 and ITGB6) with prognostic value, constituting a new (ITGs-related) prognostic marker for BLCA prognostic model, and preliminarily explored the potential molecular mechanisms of this model, providing potential targets for BLCA prognosis. We will continue to follow the progress of research on these genes in the coming work.

## Data availability statement

The original contributions presented in the study are included in the article/**Supplementary Material**. Further inquiries can be directed to the corresponding authors.

## Ethics statement

The studies involving human participants were reviewed and approved by Institutional Ethics Committee for Clinical Research and Animal Trials of the First Affiliated Hospital of Kunming Medical University [ (2022)L36]. The patients/participants provided their written informed consent to participate in this study.

## Author contributions

HT, HL, and LZ: conception and design. ZT, HW, and ZX: acquisition, analysis and interpretation of data. YJ, LG, and XX: figures drawing. HT and LZ: writing and revision of the manuscript. XL and PG: study supervision. All authors read and approved the final manuscript.

## Funding

This study was supported by the National Natural Science Foundation of China (Grant No. 81802548, 81860451), Yunnan Natural Science Foundation (Grant No. 202001AW070001, 202201AY070001-045, 202101AY070001-014, 2019FE001(-136), 2019FE001(-005)), Yunnan Health Training Project of High-Level Talents (Grant No. H2018070), Funding for Yunnan Province Clinical Research Center for Chronic Kidney Disease (Grant No. 202102AA10060), the post-graduate project of Natural Science research in Universities of Anhui Province (grant number: YJS2021A0538).

## Acknowledgments

We would like to thank everyone who took part in this study.

## Conflict of interest

The authors declare that the research was conducted in the absence of any commercial or financial relationships that could be construed as a potential conflict of interest.

The reviewer YH declared a shared parent affiliation with the authors HT, ZT, HW, YJ, ZX, PG, and XL to the handling editor at the time of review.

## Publisher’s note

All claims expressed in this article are solely those of the authors and do not necessarily represent those of their affiliated organizations, or those of the publisher, the editors and the reviewers. Any product that may be evaluated in this article, or claim that may be made by its manufacturer, is not guaranteed or endorsed by the publisher.
