# Supplementary material for: A novel prognostic model based on three integrin subunit genes-related signature for bladder cancer
Source: Front Oncol. 2022 Oct 4;12:970576. doi: 10.3389/fonc.2022.970576 (PMC9577111; doi:10.3389/fonc.2022.970576)
Supplement: Supplementary file 1 [file DataSheet_1.docx]

Supplementary Material

## Supplementary Figures

**
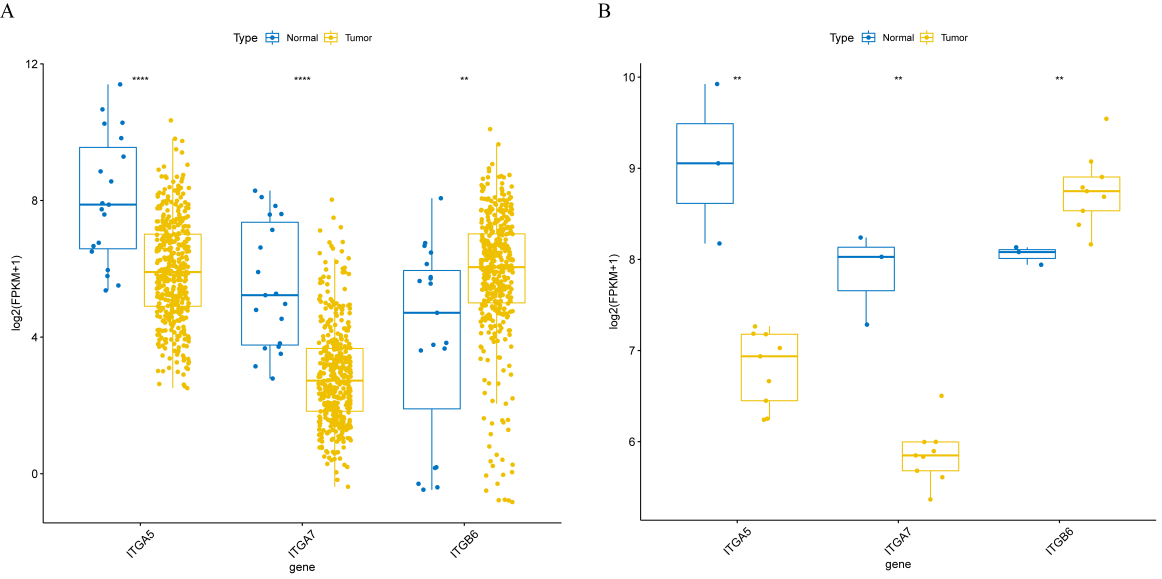
**

**Supplementary Figure 1.** The expression profiles of three prognostic genes in the TCGA-BLCA **(A)** and GSE7476 **(B)** datasets.

# Supplementary Tables

**2.1 Supplementary Table 1**. Characteristics of patients in high- and low-risk groups in training set.

|  |  | **risk** |  |  |
| --- | --- | --- | --- | --- |
|  | **Total (N=87)** | **High (N=32)** | **Low (N=55)** | **P-value** |
| **Gender,n(%)** |  |  |  |  |
| Female | 27 (31.0%) | 8 (25.0%) | 19 (34.5%) | 0.492 |
| Male | 60 (69.0%) | 24 (75.0%) | 36 (65.5%) |  |
| **M stage,** **n(%)** |  |  |  |  |
| M0 | 36 (41.4%) | 6 (18.8%) | 30 (54.5%) | <0.001 |
| M1 | 3 (3.4%) | 0 (0%) | 3 (5.5%) |  |
| MX | 48 (55.2%) | 26 (81.2%) | 22 (40.0%) |  |
| **N** **stage, n(%)** |  |  |  |  |
| N0 | 57 (65.5%) | 22 (68.8%) | 35 (63.6%) | 0.431 |
| N1 | 11 (12.6%) | 2 (6.2%) | 9 (16.4%) |  |
| N2 | 18 (20.7%) | 8 (25.0%) | 10 (18.2%) |  |
| N3 | 1 (1.1%) | 0 (0%) | 1 (1.8%) |  |
| **T stage, n(%)** |  |  |  |  |
| T2 | 5 (5.7%) | 1 (3.1%) | 4 (7.3%) | 0.654 |
| T2a | 6 (6.9%) | 3 (9.4%) | 3 (5.5%) |  |
| T2b | 16 (18.4%) | 7 (21.9%) | 9 (16.4%) |  |
| T3 | 11 (12.6%) | 2 (6.2%) | 9 (16.4%) |  |
| T3a | 17 (19.5%) | 5 (15.6%) | 12 (21.8%) |  |
| T3b | 23 (26.4%) | 10 (31.2%) | 13 (23.6%) |  |
| T4a | 8 (9.2%) | 4 (12.5%) | 4 (7.3%) |  |
| T4b | 1 (1.1%) | 0 (0%) | 1 (1.8%) |  |
| **STAGE, n(%)** |  |  |  |  |
| Stage II | 21 (24.1%) | 8 (25.0%) | 13 (23.6%) | 0.888 |
| Stage III | 36 (41.4%) | 14 (43.8%) | 22 (40.0%) |  |
| Stage IV | 30 (34.5%) | 10 (31.2%) | 20 (36.4%) |  |
| **GRADE, n(%)** |  |  |  |  |
| High Grade | 84 (96.6%) | 31 (96.9%) | 53 (96.4%) | 1 |
| Low Grade | 3 (3.4%) | 1 (3.1%) | 2 (3.6%) |  |
| **age (years), n(%)** |  |  |  |  |
| >=60 | 68 (78.2%) | 24 (75.0%) | 44 (80.0%) | 0.783 |
| <60 | 19 (21.8%) | 8 (25.0%) | 11 (20.0%) |  |

**2.2 Supplementary Table 2**. Characteristics of patients in high- and low-risk groups in testing set.

|  |  | **risk** |  |  |
| --- | --- | --- | --- | --- |
|  | **Total (N=87)** | **High (N=32)** | **Low (N=55)** | **P-value** |
| **gender, n(%)** |  |  |  |  |
| Female | 27 (31.0%) | 8 (25.0%) | 19 (34.5%) | 0.492 |
| Male | 60 (69.0%) | 24 (75.0%) | 36 (65.5%) |  |
| **M stage****,** **n(%)** |  |  |  |  |
| M0 | 36 (41.4%) | 6 (18.8%) | 30 (54.5%) | <0.001 |
| M1 | 3 (3.4%) | 0 (0%) | 3 (5.5%) |  |
| MX | 48 (55.2%) | 26 (81.2%) | 22 (40.0%) |  |
| **N** |  |  |  |  |
| N0 | 57 (65.5%) | 22 (68.8%) | 35 (63.6%) | 0.431 |
| N1 | 11 (12.6%) | 2 (6.2%) | 9 (16.4%) |  |
| N2 | 18 (20.7%) | 8 (25.0%) | 10 (18.2%) |  |
| N3 | 1 (1.1%) | 0 (0%) | 1 (1.8%) |  |
| **T stage, n(%)** |  |  |  |  |
| T2 | 5 (5.7%) | 1 (3.1%) | 4 (7.3%) | 0.654 |
| T2a | 6 (6.9%) | 3 (9.4%) | 3 (5.5%) |  |
| T2b | 16 (18.4%) | 7 (21.9%) | 9 (16.4%) |  |
| T3 | 11 (12.6%) | 2 (6.2%) | 9 (16.4%) |  |
| T3a | 17 (19.5%) | 5 (15.6%) | 12 (21.8%) |  |
| T3b | 23 (26.4%) | 10 (31.2%) | 13 (23.6%) |  |
| T4a | 8 (9.2%) | 4 (12.5%) | 4 (7.3%) |  |
| T4b | 1 (1.1%) | 0 (0%) | 1 (1.8%) |  |
| **STAGE, n(%)** |  |  |  |  |
| Stage II | 21 (24.1%) | 8 (25.0%) | 13 (23.6%) | 0.888 |
| Stage III | 36 (41.4%) | 14 (43.8%) | 22 (40.0%) |  |
| Stage IV | 30 (34.5%) | 10 (31.2%) | 20 (36.4%) |  |
| **GRADE,** **n(%)** |  |  |  |  |
| High Grade | 84 (96.6%) | 31 (96.9%) | 53 (96.4%) | 1 |
| Low Grade | 3 (3.4%) | 1 (3.1%) | 2 (3.6%) |  |
| **age (years), n(%)** |  |  |  |  |
| >=60 | 68 (78.2%) | 24 (75.0%) | 44 (80.0%) | 0.783 |
| <60 | 19 (21.8%) | 8 (25.0%) | 11 (20.0%) |  |

**2.3 Supplementary Table 3**. Characteristics of patients in high- and low-risk groups in the GSE32894 cohort.

|  |  | **risk** |  |  |
| --- | --- | --- | --- | --- |
|  | **Total (N=222)** | **high (N=90)** | **low (N=132)** | **P-value** |
|  |  |  |  |  |
| **Gender, n(%)** |  |  |  |  |
| Female | 61 (27.5%) | 21 (23.3%) | 40 (30.3%) | 0.323 |
| Male | 161 (72.5%) | 69 (76.7%) | 92 (69.7%) |  |
| **T stage, n(%)** |  |  |  |  |
| T1 | 63 (28.4%) | 31 (34.4%) | 32 (24.2%) | <0.001 |
| T2 | 40 (18.0%) | 32 (35.6%) | 8 (6.1%) |  |
| T2a | 1 (0.5%) | 1 (1.1%) | 0 (0%) |  |
| T2b | 2 (0.9%) | 2 (2.2%) | 0 (0%) |  |
| T3 | 1 (0.5%) | 1 (1.1%) | 0 (0%) |  |
| T3b | 5 (2.3%) | 4 (4.4%) | 1 (0.8%) |  |
| T4a | 1 (0.5%) | 0 (0%) | 1 (0.8%) |  |
| Ta | 109 (49.1%) | 19 (21.1%) | 90 (68.2%) |  |
| **GRADE, n(%)** |  |  |  |  |
| G1 | 45 (20.3%) | 4 (4.4%) | 41 (31.1%) | <0.001 |
| G2 | 84 (37.8%) | 23 (25.6%) | 61 (46.2%) |  |
| G3 | 93 (41.9%) | 63 (70.0%) | 30 (22.7%) |  |
| **age (years), n(%)** |  |  |  |  |
| >=60 | 184 (82.9%) | 79 (87.8%) | 105 (79.5%) | 0.156 |
| <60 | 38 (17.1%) | 11 (12.2%) | 27 (20.5%) |  |

**2.4 Supplementary Table 4**. 1719 GO pathways enriched by GSEA.

| **NO.** | **ID** | **NES** | **P value** | **P.adjust** | **Q values** |
| --- | --- | --- | --- | --- | --- |
| 1 | GOBP_ACTIVATION_OF_IMMUNE_RESPONSE | 1.734 | <0.001 | <0.001 | <0.001 |
| 2 | GOBP_ADAPTIVE_IMMUNE_RESPONSE | 1.757 | <0.001 | <0.001 | <0.001 |
| 3 | GOBP_ADAPTIVE_IMMUNE_RESPONSE_BASED_ON_SOMATIC_RECOMBINATION_OF_IMMUNE_RECEPTORS_BUILT_FROM_IMMUNOGLOBULIN_SUPERFAMILY_DOMAINS | 1.728 | <0.001 | <0.001 | <0.001 |
| 4 | GOBP_B_CELL_ACTIVATION | 1.757 | <0.001 | <0.001 | <0.001 |
| 5 | GOBP_CALCIUM_ION_TRANSPORT | 1.656 | <0.001 | <0.001 | <0.001 |
| 6 | GOBP_CARTILAGE_DEVELOPMENT | 1.854 | <0.001 | <0.001 | <0.001 |
| 7 | GOBP_CELL_CHEMOTAXIS | 1.837 | <0.001 | <0.001 | <0.001 |
| 8 | GOBP_CELL_SUBSTRATE_ADHESION | 1.714 | <0.001 | <0.001 | <0.001 |
| 9 | GOBP_CONNECTIVE_TISSUE_DEVELOPMENT | 1.803 | <0.001 | <0.001 | <0.001 |
| 10 | GOBP_CYTOKINE_MEDIATED_SIGNALING_PATHWAY | 1.612 | <0.001 | <0.001 | <0.001 |
| 11 | GOBP_EXTERNAL_ENCAPSULATING_STRUCTURE_ORGANIZATION | 2.001 | <0.001 | <0.001 | <0.001 |
| 12 | GOBP_GRANULOCYTE_CHEMOTAXIS | 1.896 | <0.001 | <0.001 | <0.001 |
| 13 | GOBP_GRANULOCYTE_MIGRATION | 1.922 | <0.001 | <0.001 | <0.001 |
| 14 | GOBP_HUMORAL_IMMUNE_RESPONSE | 1.793 | <0.001 | <0.001 | <0.001 |
| 15 | GOBP_LEUKOCYTE_CELL_CELL_ADHESION | 1.718 | <0.001 | <0.001 | <0.001 |
| 16 | GOBP_LEUKOCYTE_CHEMOTAXIS | 1.848 | <0.001 | <0.001 | <0.001 |
| 17 | GOBP_LEUKOCYTE_MEDIATED_IMMUNITY | 1.698 | <0.001 | <0.001 | <0.001 |
| 18 | GOBP_LEUKOCYTE_MIGRATION | 1.852 | <0.001 | <0.001 | <0.001 |
| 19 | GOBP_LEUKOCYTE_PROLIFERATION | 1.748 | <0.001 | <0.001 | <0.001 |
| 20 | GOBP_MONONUCLEAR_CELL_DIFFERENTIATION | 1.648 | <0.001 | <0.001 | <0.001 |
| 21 | GOBP_MONONUCLEAR_CELL_MIGRATION | 1.843 | <0.001 | <0.001 | <0.001 |
| 22 | GOBP_MUSCLE_CELL_DIFFERENTIATION | 1.750 | <0.001 | <0.001 | <0.001 |
| 23 | GOBP_MUSCLE_CONTRACTION | 1.760 | <0.001 | <0.001 | <0.001 |
| 24 | GOBP_MUSCLE_ORGAN_DEVELOPMENT | 1.789 | <0.001 | <0.001 | <0.001 |
| 25 | GOBP_MUSCLE_SYSTEM_PROCESS | 1.726 | <0.001 | <0.001 | <0.001 |
| 26 | GOBP_MUSCLE_TISSUE_DEVELOPMENT | 1.663 | <0.001 | <0.001 | <0.001 |
| 27 | GOBP_MYELOID_LEUKOCYTE_MIGRATION | 1.953 | <0.001 | <0.001 | <0.001 |
| 28 | GOBP_NEGATIVE_REGULATION_OF_IMMUNE_SYSTEM_PROCESS | 1.764 | <0.001 | <0.001 | <0.001 |
| 29 | GOBP_NEUTROPHIL_CHEMOTAXIS | 1.933 | <0.001 | <0.001 | <0.001 |
| 30 | GOBP_NEUTROPHIL_MIGRATION | 1.948 | <0.001 | <0.001 | <0.001 |
| 31 | GOBP_OSSIFICATION | 1.757 | <0.001 | <0.001 | <0.001 |
| 32 | GOBP_PHAGOCYTOSIS | 1.881 | <0.001 | <0.001 | <0.001 |
| 33 | GOBP_POSITIVE_REGULATION_OF_CELL_ACTIVATION | 1.778 | <0.001 | <0.001 | <0.001 |
| 34 | GOBP_POSITIVE_REGULATION_OF_CELL_ADHESION | 1.741 | <0.001 | <0.001 | <0.001 |
| 35 | GOBP_POSITIVE_REGULATION_OF_CYTOKINE_PRODUCTION | 1.654 | <0.001 | <0.001 | <0.001 |
| 36 | GOBP_POSITIVE_REGULATION_OF_ERK1_AND_ERK2_CASCADE | 1.856 | <0.001 | <0.001 | <0.001 |
| 37 | GOBP_POSITIVE_REGULATION_OF_MAPK_CASCADE | 1.715 | <0.001 | <0.001 | <0.001 |
| 38 | GOBP_POSITIVE_REGULATION_OF_RESPONSE_TO_EXTERNAL_STIMULUS | 1.688 | <0.001 | <0.001 | <0.001 |
| 39 | GOBP_REGULATION_OF_CELLULAR_RESPONSE_TO_GROWTH_FACTOR_STIMULUS | 1.753 | <0.001 | <0.001 | <0.001 |
| 40 | GOBP_REGULATION_OF_CYTOSOLIC_CALCIUM_ION_CONCENTRATION | 1.748 | <0.001 | <0.001 | <0.001 |
| 41 | GOBP_REGULATION_OF_IMMUNE_EFFECTOR_PROCESS | 1.670 | <0.001 | <0.001 | <0.001 |
| 42 | GOBP_REGULATION_OF_LEUKOCYTE_MIGRATION | 1.807 | <0.001 | <0.001 | <0.001 |
| 43 | GOBP_REGULATION_OF_LEUKOCYTE_PROLIFERATION | 1.745 | <0.001 | <0.001 | <0.001 |
| 44 | GOBP_REGULATION_OF_LYMPHOCYTE_ACTIVATION | 1.733 | <0.001 | <0.001 | <0.001 |
| 45 | GOBP_REGULATION_OF_T_CELL_ACTIVATION | 1.699 | <0.001 | <0.001 | <0.001 |
| 46 | GOBP_REGULATION_OF_VASCULATURE_DEVELOPMENT | 1.739 | <0.001 | <0.001 | <0.001 |
| 47 | GOBP_T_CELL_ACTIVATION | 1.685 | <0.001 | <0.001 | <0.001 |
| 48 | GOCC_ACTIN_CYTOSKELETON | 1.594 | <0.001 | <0.001 | <0.001 |
| 49 | GOCC_CELL_SUBSTRATE_JUNCTION | 1.631 | <0.001 | <0.001 | <0.001 |
| 50 | GOCC_COLLAGEN_CONTAINING_EXTRACELLULAR_MATRIX | 2.080 | <0.001 | <0.001 | <0.001 |
| 51 | GOCC_COLLAGEN_TRIMER | 2.045 | <0.001 | <0.001 | <0.001 |
| 52 | GOCC_CONTRACTILE_FIBER | 1.798 | <0.001 | <0.001 | <0.001 |
| 53 | GOCC_ENDOPLASMIC_RETICULUM_LUMEN | 1.820 | <0.001 | <0.001 | <0.001 |
| 54 | GOCC_EXTERNAL_SIDE_OF_PLASMA_MEMBRANE | 1.904 | <0.001 | <0.001 | <0.001 |
| 55 | GOMF_CYTOKINE_BINDING | 1.938 | <0.001 | <0.001 | <0.001 |
| 56 | GOMF_EXTRACELLULAR_MATRIX_STRUCTURAL_CONSTITUENT | 2.221 | <0.001 | <0.001 | <0.001 |
| 57 | GOMF_GLYCOSAMINOGLYCAN_BINDING | 1.994 | <0.001 | <0.001 | <0.001 |
| 58 | GOMF_HEPARIN_BINDING | 1.992 | <0.001 | <0.001 | <0.001 |
| 59 | GOMF_IMMUNE_RECEPTOR_ACTIVITY | 1.873 | <0.001 | <0.001 | <0.001 |
| 60 | GOMF_SULFUR_COMPOUND_BINDING | 1.827 | <0.001 | <0.001 | <0.001 |
| 61 | HP_ABNORMALITY_OF_THE_SYNOVIA | 1.700 | <0.001 | <0.001 | <0.001 |
| 62 | HP_ABNORMAL_BLEEDING | 1.654 | <0.001 | <0.001 | <0.001 |
| 63 | HP_ABNORMAL_HEART_VALVE_MORPHOLOGY | 1.696 | <0.001 | <0.001 | <0.001 |
| 64 | HP_ABNORMAL_HEART_VALVE_PHYSIOLOGY | 1.740 | <0.001 | <0.001 | <0.001 |
| 65 | HP_LIMITATION_OF_JOINT_MOBILITY | 1.665 | <0.001 | <0.001 | <0.001 |
| 66 | HP_SUBCUTANEOUS_HEMORRHAGE | 1.823 | <0.001 | <0.001 | <0.001 |
| 67 | GOBP_COLLAGEN_FIBRIL_ORGANIZATION | 2.025 | <0.001 | <0.001 | <0.001 |
| 68 | GOMF_INTEGRIN_BINDING | 1.860 | <0.001 | <0.001 | <0.001 |
| 69 | GOBP_ERK1_AND_ERK2_CASCADE | 1.669 | <0.001 | <0.001 | <0.001 |
| 70 | GOBP_REGULATION_OF_BLOOD_CIRCULATION | 1.750 | <0.001 | <0.001 | <0.001 |
| 71 | HP_ABNORMAL_CEREBRAL_VASCULAR_MORPHOLOGY | 1.710 | <0.001 | <0.001 | <0.001 |
| 72 | HP_ABNORMALITY_OF_THE_KNEE | 1.658 | <0.001 | <0.001 | <0.001 |
| 73 | GOBP_POSITIVE_REGULATION_OF_LEUKOCYTE_CELL_CELL_ADHESION | 1.754 | <0.001 | <0.001 | <0.001 |
| 74 | GOBP_BONE_DEVELOPMENT | 1.772 | <0.001 | <0.001 | <0.001 |
| 75 | GOBP_REGULATION_OF_METAL_ION_TRANSPORT | 1.631 | <0.001 | <0.001 | <0.001 |
| 76 | GOBP_NEGATIVE_REGULATION_OF_CELL_ACTIVATION | 1.787 | <0.001 | <0.001 | <0.001 |
| 77 | GOBP_CELL_ACTIVATION_INVOLVED_IN_IMMUNE_RESPONSE | 1.704 | <0.001 | <0.001 | <0.001 |
| 78 | GOBP_REGULATION_OF_LEUKOCYTE_DIFFERENTIATION | 1.711 | <0.001 | <0.001 | <0.001 |
| 79 | HP_JOINT_DISLOCATION | 1.650 | <0.001 | <0.001 | <0.001 |
| 80 | GOMF_G_PROTEIN_COUPLED_RECEPTOR_BINDING | 1.719 | <0.001 | <0.001 | <0.001 |
| 81 | GOBP_REGULATION_OF_TRANS_SYNAPTIC_SIGNALING | 1.601 | <0.001 | <0.001 | <0.001 |
| 82 | GOBP_POSITIVE_REGULATION_OF_VASCULATURE_DEVELOPMENT | 1.809 | <0.001 | <0.001 | <0.001 |
| 83 | GOBP_COLLAGEN_METABOLIC_PROCESS | 1.910 | <0.001 | <0.001 | <0.001 |
| 84 | GOBP_RESPONSE_TO_CHEMOKINE | 1.910 | <0.001 | <0.001 | <0.001 |
| 85 | GOMF_AMIDE_BINDING | 1.616 | <0.001 | <0.001 | <0.001 |
| 86 | GOBP_REGULATION_OF_CHEMOTAXIS | 1.763 | <0.001 | <0.001 | <0.001 |
| 87 | GOMF_CARBOHYDRATE_BINDING | 1.703 | <0.001 | <0.001 | <0.001 |
| 88 | GOBP_POSITIVE_REGULATION_OF_CELL_CELL_ADHESION | 1.696 | <0.001 | <0.001 | <0.001 |
| 89 | HP_ABNORMALITY_OF_THE_ELBOW | 1.743 | <0.001 | <0.001 | <0.001 |
| 90 | GOBP_SECOND_MESSENGER_MEDIATED_SIGNALING | 1.676 | <0.001 | <0.001 | <0.001 |
| 91 | HP_ARTHRALGIA | 1.778 | <0.001 | <0.001 | <0.001 |
| 92 | GOBP_REGULATION_OF_INFLAMMATORY_RESPONSE | 1.628 | <0.001 | <0.001 | <0.001 |
| 93 | HP_ABNORMAL_SCLERA_MORPHOLOGY | 1.868 | <0.001 | <0.001 | <0.001 |
| 94 | HP_ABNORMAL_ADIPOSE_TISSUE_MORPHOLOGY | 1.757 | <0.001 | <0.001 | <0.001 |
| 95 | GOBP_REGULATION_OF_CELL_CELL_ADHESION | 1.583 | <0.001 | <0.001 | <0.001 |
| 96 | GOBP_HEART_PROCESS | 1.706 | <0.001 | <0.001 | <0.001 |
| 97 | HP_ABNORMAL_SYSTEMIC_ARTERIAL_MORPHOLOGY | 1.565 | <0.001 | <0.001 | <0.001 |
| 98 | GOBP_MUSCLE_CELL_DEVELOPMENT | 1.781 | <0.001 | <0.001 | <0.001 |
| 99 | HP_ABNORMALITY_OF_THE_ABDOMINAL_WALL | 1.556 | <0.001 | <0.001 | <0.001 |
| 100 | HP_ABNORMALITY_OF_UPPER_LIMB_JOINT | 1.564 | <0.001 | <0.001 | <0.001 |
| 101 | GOBP_MYELOID_LEUKOCYTE_ACTIVATION | 1.735 | <0.001 | <0.001 | <0.001 |
| 102 | GOBP_REGULATION_OF_CATION_TRANSMEMBRANE_TRANSPORT | 1.634 | <0.001 | <0.001 | <0.001 |
| 103 | HP_JOINT_HYPERMOBILITY | 1.636 | <0.001 | <0.001 | <0.001 |
| 104 | HP_ABNORMAL_MITRAL_VALVE_PHYSIOLOGY | 1.814 | <0.001 | <0.001 | <0.001 |
| 105 | GOBP_REGULATION_OF_CALCIUM_ION_TRANSPORT | 1.702 | <0.001 | <0.001 | <0.001 |
| 106 | HP_BRUISING_SUSCEPTIBILITY | 1.807 | <0.001 | <0.001 | <0.001 |
| 107 | HP_BLUE_SCLERAE | 1.908 | <0.001 | <0.001 | <0.001 |
| 108 | GOBP_CELL_MATRIX_ADHESION | 1.717 | <0.001 | <0.001 | <0.001 |
| 109 | HP_ABNORMALITY_OF_THE_LYMPH_NODES | 1.738 | <0.001 | <0.001 | <0.001 |
| 110 | GOCC_I_BAND | 1.809 | <0.001 | <0.001 | <0.001 |
| 111 | GOBP_REGULATION_OF_HEMOPOIESIS | 1.618 | <0.001 | <0.001 | <0.001 |
| 112 | GOBP_MESENCHYME_DEVELOPMENT | 1.674 | <0.001 | <0.001 | <0.001 |
| 113 | GOBP_BONE_MINERALIZATION | 1.832 | <0.001 | <0.001 | <0.001 |
| 114 | GOCC_MEMBRANE_MICRODOMAIN | 1.642 | <0.001 | <0.001 | <0.001 |
| 115 | GOCC_ENDOCYTIC_VESICLE | 1.639 | <0.001 | <0.001 | <0.001 |
| 116 | GOBP_TRANSMEMBRANE_RECEPTOR_PROTEIN_SERINE_THREONINE_KINASE_SIGNALING_PATHWAY | 1.611 | <0.001 | <0.001 | <0.001 |
| 117 | GOBP_REGULATION_OF_ION_TRANSMEMBRANE_TRANSPORT | 1.563 | <0.001 | <0.001 | <0.001 |
| 118 | GOMF_PEPTIDE_BINDING | 1.648 | <0.001 | <0.001 | <0.001 |
| 119 | GOBP_STRIATED_MUSCLE_CELL_DIFFERENTIATION | 1.681 | <0.001 | <0.001 | <0.001 |
| 120 | GOCC_SARCOLEMMA | 1.821 | <0.001 | <0.001 | <0.001 |
| 121 | GOBP_TUMOR_NECROSIS_FACTOR_SUPERFAMILY_CYTOKINE_PRODUCTION | 1.750 | <0.001 | <0.001 | <0.001 |
| 122 | HP_ABNORMAL_ATRIOVENTRICULAR_VALVE_PHYSIOLOGY | 1.770 | <0.001 | <0.001 | <0.001 |
| 123 | GOBP_REGULATION_OF_ANATOMICAL_STRUCTURE_SIZE | 1.533 | <0.001 | <0.001 | <0.001 |
| 124 | GOBP_CALCIUM_ION_TRANSMEMBRANE_TRANSPORT | 1.633 | <0.001 | <0.001 | <0.001 |
| 125 | HP_ATRIAL_ARRHYTHMIA | 1.906 | <0.001 | <0.001 | <0.001 |
| 126 | GOBP_B_CELL_DIFFERENTIATION | 1.811 | <0.001 | <0.001 | <0.001 |
| 127 | GOBP_B_CELL_MEDIATED_IMMUNITY | 1.781 | <0.001 | <0.001 | <0.001 |
| 128 | HP_SUPRAVENTRICULAR_ARRHYTHMIA | 1.852 | <0.001 | <0.001 | <0.001 |
| 129 | GOBP_CALCIUM_ION_TRANSPORT_INTO_CYTOSOL | 1.764 | <0.001 | <0.001 | <0.001 |
| 130 | GOMF_COLLAGEN_BINDING | 1.931 | <0.001 | <0.001 | <0.001 |
| 131 | GOBP_CYTOSOLIC_CALCIUM_ION_TRANSPORT | 1.744 | <0.001 | <0.001 | <0.001 |
| 132 | GOBP_CELL_GROWTH | 1.541 | <0.001 | <0.001 | <0.001 |
| 133 | HP_ABNORMAL_AORTIC_MORPHOLOGY | 1.595 | <0.001 | <0.001 | <0.001 |
| 134 | GOBP_LYMPHOCYTE_MEDIATED_IMMUNITY | 1.653 | <0.001 | <0.001 | <0.001 |
| 135 | HP_PES_PLANUS | 1.620 | <0.001 | <0.001 | <0.001 |
| 136 | GOCC_SECRETORY_GRANULE_MEMBRANE | 1.625 | <0.001 | <0.001 | <0.001 |
| 137 | HP_ABNORMAL_STERNUM_MORPHOLOGY | 1.567 | <0.001 | <0.001 | <0.001 |
| 138 | HP_ABNORMAL_CARDIAC_VENTRICULAR_FUNCTION | 1.867 | <0.001 | <0.001 | <0.001 |
| 139 | GOMF_PEPTIDE_RECEPTOR_ACTIVITY | 1.778 | <0.001 | <0.001 | <0.001 |
| 140 | HP_ABNORMALITY_OF_HUMORAL_IMMUNITY | 1.657 | <0.001 | <0.001 | <0.001 |
| 141 | HP_ABNORMAL_VERTEBRAL_MORPHOLOGY | 1.546 | <0.001 | <0.001 | <0.001 |
| 142 | HP_LIPODYSTROPHY | 1.804 | <0.001 | <0.001 | <0.001 |
| 143 | HP_SLENDER_FINGER | 1.778 | <0.001 | <0.001 | <0.001 |
| 144 | HP_AUTOIMMUNITY | 1.738 | <0.001 | <0.001 | <0.001 |
| 145 | GOBP_PEPTIDYL_TYROSINE_MODIFICATION | 1.586 | <0.001 | <0.001 | <0.001 |
| 146 | GOMF_ACTIN_BINDING | 1.543 | <0.001 | <0.001 | <0.001 |
| 147 | GOBP_IMMUNE_RESPONSE_REGULATING_CELL_SURFACE_RECEPTOR_SIGNALING_PATHWAY | 1.658 | <0.001 | <0.001 | <0.001 |
| 148 | GOBP_LYMPHOCYTE_MIGRATION | 1.815 | <0.001 | <0.001 | <0.001 |
| 149 | GOBP_REGULATION_OF_PHAGOCYTOSIS | 1.845 | <0.001 | <0.001 | <0.001 |
| 150 | GOMF_GROWTH_FACTOR_BINDING | 1.775 | <0.001 | <0.001 | <0.001 |
| 151 | GOCC_PLATELET_ALPHA_GRANULE | 1.867 | <0.001 | <0.001 | <0.001 |
| 152 | GOBP_REGULATION_OF_BIOMINERALIZATION | 1.841 | <0.001 | <0.001 | <0.001 |
| 153 | HP_ABNORMAL_CEREBRAL_ARTERY_MORPHOLOGY | 1.920 | <0.001 | <0.001 | <0.001 |
| 154 | HP_AORTIC_ROOT_ANEURYSM | 1.845 | <0.001 | <0.001 | <0.001 |
| 155 | HP_ABNORMAL_ORAL_MUCOSA_MORPHOLOGY | 1.607 | <0.001 | <0.001 | <0.001 |
| 156 | GOBP_REGULATION_OF_HEART_CONTRACTION | 1.704 | <0.001 | <0.001 | <0.001 |
| 157 | HP_PROPTOSIS | 1.654 | <0.001 | <0.001 | <0.001 |
| 158 | GOBP_REGULATION_OF_TRANSMEMBRANE_RECEPTOR_PROTEIN_SERINE_THREONINE_KINASE_SIGNALING_PATHWAY | 1.660 | <0.001 | <0.001 | <0.001 |
| 159 | GOBP_HEMOSTASIS | 1.690 | <0.001 | <0.001 | <0.001 |
| 160 | GOBP_POSITIVE_REGULATION_OF_LEUKOCYTE_MIGRATION | 1.790 | <0.001 | <0.001 | <0.001 |
| 161 | GOBP_SEQUESTERING_OF_CALCIUM_ION | 1.782 | <0.001 | <0.001 | <0.001 |
| 162 | GOBP_CHONDROCYTE_DIFFERENTIATION | 1.840 | <0.001 | <0.001 | <0.001 |
| 163 | GOBP_ENDOTHELIAL_CELL_PROLIFERATION | 1.717 | <0.001 | <0.001 | <0.001 |
| 164 | GOBP_COMPLEMENT_ACTIVATION | 1.927 | <0.001 | <0.001 | <0.001 |
| 165 | HP_ABNORMAL_PLEURA_MORPHOLOGY | 1.761 | <0.001 | <0.001 | <0.001 |
| 166 | HP_ABNORMAL_VASCULAR_PHYSIOLOGY | 1.643 | <0.001 | <0.001 | <0.001 |
| 167 | GOBP_MAINTENANCE_OF_LOCATION | 1.611 | <0.001 | <0.001 | <0.001 |
| 168 | GOBP_ADENYLATE_CYCLASE_MODULATING_G_PROTEIN_COUPLED_RECEPTOR_SIGNALING_PATHWAY | 1.655 | <0.001 | <0.001 | <0.001 |
| 169 | GOCC_RECEPTOR_COMPLEX | 1.550 | <0.001 | <0.001 | <0.001 |
| 170 | GOBP_MONOCYTE_CHEMOTAXIS | 1.910 | <0.001 | <0.001 | <0.001 |
| 171 | GOMF_EXTRACELLULAR_MATRIX_STRUCTURAL_CONSTITUENT_CONFERRING_TENSILE_STRENGTH | 1.920 | <0.001 | <0.001 | <0.001 |
| 172 | GOBP_POSITIVE_REGULATION_OF_CHEMOTAXIS | 1.771 | <0.001 | <0.001 | <0.001 |
| 173 | GOBP_REGULATION_OF_WNT_SIGNALING_PATHWAY | 1.604 | <0.001 | <0.001 | <0.001 |
| 174 | HP_ABNORMAL_FORM_OF_THE_VERTEBRAL_BODIES | 1.606 | <0.001 | <0.001 | <0.001 |
| 175 | GOBP_NEGATIVE_REGULATION_OF_RESPONSE_TO_EXTERNAL_STIMULUS | 1.546 | <0.001 | <0.001 | <0.001 |
| 176 | HP_ABNORMALITY_OF_BLOOD_CIRCULATION | 1.612 | <0.001 | <0.001 | <0.001 |
| 177 | GOBP_IMMUNE_RESPONSE_REGULATING_SIGNALING_PATHWAY | 1.545 | <0.001 | <0.001 | <0.001 |
| 178 | HP_INGUINAL_HERNIA | 1.655 | <0.001 | <0.001 | <0.001 |
| 179 | HP_SCAPULAR_WINGING | 1.879 | <0.001 | <0.001 | <0.001 |
| 180 | GOBP_T_CELL_DIFFERENTIATION | 1.640 | <0.001 | <0.001 | <0.001 |
| 181 | GOBP_MAINTENANCE_OF_LOCATION_IN_CELL | 1.671 | <0.001 | <0.001 | <0.001 |
| 182 | HP_ABNORMAL_ATRIOVENTRICULAR_VALVE_MORPHOLOGY | 1.757 | <0.001 | <0.001 | <0.001 |
| 183 | GOBP_POSITIVE_REGULATION_OF_HEMOPOIESIS | 1.748 | <0.001 | <0.001 | <0.001 |
| 184 | HP_HYPERLORDOSIS | 1.700 | <0.001 | <0.001 | <0.001 |
| 185 | HP_LONG_FINGERS | 1.652 | <0.001 | <0.001 | <0.001 |
| 186 | GOBP_RESPONSE_TO_MOLECULE_OF_BACTERIAL_ORIGIN | 1.587 | <0.001 | <0.001 | <0.001 |
| 187 | GOBP_CELLULAR_RESPONSE_TO_BIOTIC_STIMULUS | 1.666 | <0.001 | <0.001 | <0.001 |
| 188 | GOBP_OSTEOBLAST_DIFFERENTIATION | 1.668 | <0.001 | <0.001 | <0.001 |
| 189 | HP_AORTIC_ANEURYSM | 1.694 | <0.001 | <0.001 | <0.001 |
| 190 | GOBP_MUSCLE_CELL_PROLIFERATION | 1.682 | <0.001 | <0.001 | <0.001 |
| 191 | GOBP_REGULATION_OF_LEUKOCYTE_CHEMOTAXIS | 1.776 | <0.001 | <0.001 | <0.001 |
| 192 | GOBP_ACTIN_FILAMENT_ORGANIZATION | 1.510 | <0.001 | <0.001 | <0.001 |
| 193 | GOBP_CALCIUM_MEDIATED_SIGNALING | 1.693 | <0.001 | <0.001 | <0.001 |
| 194 | GOBP_POSITIVE_REGULATION_OF_IMMUNE_EFFECTOR_PROCESS | 1.637 | <0.001 | <0.001 | <0.001 |
| 195 | HP_KYPHOSIS | 1.496 | <0.001 | <0.001 | <0.001 |
| 196 | GOCC_BASEMENT_MEMBRANE | 1.815 | <0.001 | <0.001 | <0.001 |
| 197 | GOBP_SMOOTH_MUSCLE_CELL_PROLIFERATION | 1.730 | <0.001 | <0.001 | <0.001 |
| 198 | HP_HYPEREXTENSIBLE_SKIN | 1.890 | <0.001 | <0.001 | <0.001 |
| 199 | HP_INTRACRANIAL_HEMORRHAGE | 1.744 | <0.001 | <0.001 | <0.001 |
| 200 | GOBP_NEGATIVE_REGULATION_OF_LOCOMOTION | 1.568 | <0.001 | <0.001 | <0.001 |
| 201 | GOBP_CELLULAR_RESPONSE_TO_MOLECULE_OF_BACTERIAL_ORIGIN | 1.675 | <0.001 | <0.001 | <0.001 |
| 202 | HP_ABNORMAL_AORTIC_VALVE_PHYSIOLOGY | 1.743 | <0.001 | <0.001 | <0.001 |
| 203 | GOBP_CARDIAC_MUSCLE_TISSUE_DEVELOPMENT | 1.659 | <0.001 | <0.001 | <0.001 |
| 204 | GOBP_WOUND_HEALING | 1.538 | <0.001 | <0.001 | <0.001 |
| 205 | HP_SKIN_NODULE | 1.808 | <0.001 | <0.001 | <0.001 |
| 206 | GOBP_VASCULAR_PROCESS_IN_CIRCULATORY_SYSTEM | 1.627 | <0.001 | <0.001 | <0.001 |
| 207 | HP_PLATYSPONDYLY | 1.766 | <0.001 | <0.001 | <0.001 |
| 208 | GOBP_REGULATION_OF_ACTIN_FILAMENT_BASED_PROCESS | 1.547 | <0.001 | <0.001 | <0.001 |
| 209 | GOBP_CELL_RECOGNITION | 1.739 | <0.001 | <0.001 | <0.001 |
| 210 | GOBP_POSITIVE_REGULATION_OF_CALCIUM_ION_TRANSPORT | 1.775 | <0.001 | <0.001 | <0.001 |
| 211 | HP_KYPHOSCOLIOSIS | 1.693 | <0.001 | <0.001 | <0.001 |
| 212 | GOBP_OVULATION_CYCLE | 1.872 | <0.001 | <0.001 | <0.001 |
| 213 | HP_MENINGITIS | 1.854 | <0.001 | <0.001 | <0.001 |
| 214 | GOBP_POSITIVE_REGULATION_OF_LEUKOCYTE_PROLIFERATION | 1.719 | <0.001 | <0.001 | <0.001 |
| 215 | GOBP_POSITIVE_REGULATION_OF_DEFENSE_RESPONSE | 1.600 | <0.001 | <0.001 | <0.001 |
| 216 | GOCC_ENDOCYTIC_VESICLE_MEMBRANE | 1.676 | <0.001 | <0.001 | <0.001 |
| 217 | GOBP_BIOMINERALIZATION | 1.697 | <0.001 | <0.001 | <0.001 |
| 218 | GOMF_CCR_CHEMOKINE_RECEPTOR_BINDING | 1.890 | <0.001 | <0.001 | <0.001 |
| 219 | HP_MITRAL_VALVE_PROLAPSE | 1.791 | <0.001 | <0.001 | <0.001 |
| 220 | GOCC_FIBRILLAR_COLLAGEN_TRIMER | 1.830 | <0.001 | <0.001 | <0.001 |
| 221 | GOBP_REGULATION_OF_BONE_MINERALIZATION | 1.864 | <0.001 | <0.001 | <0.001 |
| 222 | HP_HIP_DISLOCATION | 1.641 | <0.001 | <0.001 | <0.001 |
| 223 | HP_ABNORMAL_DIAPHYSIS_MORPHOLOGY | 1.586 | <0.001 | <0.001 | <0.001 |
| 224 | HP_HEMOPTYSIS | 1.861 | <0.001 | <0.001 | <0.001 |
| 225 | GOBP_AXON_DEVELOPMENT | 1.501 | <0.001 | <0.001 | <0.001 |
| 226 | HP_LOWER_EXTREMITY_JOINT_DISLOCATION | 1.604 | <0.001 | <0.001 | <0.001 |
| 227 | GOBP_REGULATION_OF_G_PROTEIN_COUPLED_RECEPTOR_SIGNALING_PATHWAY | 1.734 | <0.001 | <0.001 | <0.001 |
| 228 | GOMF_CYTOKINE_ACTIVITY | 1.633 | <0.001 | <0.001 | <0.001 |
| 229 | GOBP_REGULATION_OF_BLOOD_PRESSURE | 1.675 | <0.001 | <0.001 | <0.001 |
| 230 | GOBP_NEGATIVE_REGULATION_OF_WNT_SIGNALING_PATHWAY | 1.685 | <0.001 | <0.001 | <0.001 |
| 231 | GOBP_REGULATION_OF_CELL_DEVELOPMENT | 1.482 | <0.001 | <0.001 | <0.001 |
| 232 | GOBP_NEGATIVE_REGULATION_OF_TRANSPORT | 1.497 | <0.001 | <0.001 | <0.001 |
| 233 | GOBP_AMEBOIDAL_TYPE_CELL_MIGRATION | 1.499 | <0.001 | <0.001 | <0.001 |
| 234 | HP_ABNORMAL_ARTERIAL_PHYSIOLOGY | 1.719 | <0.001 | <0.001 | <0.001 |
| 235 | HP_PHENOTYPIC_VARIABILITY | 1.533 | <0.001 | <0.001 | <0.001 |
| 236 | HP_VASCULAR_DILATATION | 1.621 | <0.001 | <0.001 | <0.001 |
| 237 | GOBP_MACROPHAGE_ACTIVATION | 1.789 | <0.001 | <0.001 | <0.001 |
| 238 | HP_SUBCUTANEOUS_NODULE | 1.821 | <0.001 | <0.001 | <0.001 |
| 239 | GOMF_COMPLEMENT_BINDING | 1.839 | <0.001 | <0.001 | <0.001 |
| 240 | GOBP_NEGATIVE_REGULATION_OF_TRANSMEMBRANE_RECEPTOR_PROTEIN_SERINE_THREONINE_KINASE_SIGNALING_PATHWAY | 1.737 | <0.001 | <0.001 | <0.001 |
| 241 | HP_ARACHNODACTYLY | 1.740 | <0.001 | <0.001 | <0.001 |
| 242 | GOBP_RECEPTOR_MEDIATED_ENDOCYTOSIS | 1.617 | <0.001 | <0.001 | <0.001 |
| 243 | HP_WADDLING_GAIT | 1.769 | <0.001 | <0.001 | <0.001 |
| 244 | GOBP_CELLULAR_EXTRAVASATION | 1.837 | <0.001 | <0.001 | <0.001 |
| 245 | HP_SKELETAL_MUSCLE_ATROPHY | 1.478 | <0.001 | <0.001 | <0.001 |
| 246 | HP_LIPOATROPHY | 1.845 | <0.001 | <0.001 | <0.001 |
| 247 | GOBP_B_CELL_RECEPTOR_SIGNALING_PATHWAY | 1.873 | <0.001 | <0.001 | <0.001 |
| 248 | GOBP_POSITIVE_REGULATION_OF_PHAGOCYTOSIS | 1.830 | <0.001 | <0.001 | <0.001 |
| 249 | HP_THORACIC_AORTIC_ANEURYSM | 1.709 | <0.001 | <0.001 | <0.001 |
| 250 | GOBP_SKELETAL_SYSTEM_MORPHOGENESIS | 1.621 | <0.001 | <0.001 | <0.001 |
| 251 | GOBP_CELL_CELL_SIGNALING_BY_WNT | 1.493 | <0.001 | <0.001 | <0.001 |
| 252 | GOMF_CYTOKINE_RECEPTOR_BINDING | 1.597 | <0.001 | <0.001 | <0.001 |
| 253 | GOBP_REGULATION_OF_OSSIFICATION | 1.767 | <0.001 | <0.001 | <0.001 |
| 254 | HP_DILATATION_OF_THE_CEREBRAL_ARTERY | 1.853 | <0.001 | <0.001 | <0.001 |
| 255 | GOBP_POSITIVE_REGULATION_OF_CELL_DEVELOPMENT | 1.569 | <0.001 | <0.001 | <0.001 |
| 256 | GOBP_INTERLEUKIN_6_PRODUCTION | 1.719 | <0.001 | <0.001 | <0.001 |
| 257 | HP_ABNORMALITY_OF_CARDIOVASCULAR_SYSTEM_ELECTROPHYSIOLOGY | 1.497 | <0.001 | <0.001 | <0.001 |
| 258 | GOBP_HEART_MORPHOGENESIS | 1.609 | <0.001 | <0.001 | <0.001 |
| 259 | GOBP_REGULATION_OF_SUPRAMOLECULAR_FIBER_ORGANIZATION | 1.536 | <0.001 | <0.001 | <0.001 |
| 260 | HP_ABNORMALITY_OF_THE_CALF | 1.592 | <0.001 | <0.001 | <0.001 |
| 261 | GOBP_POSITIVE_REGULATION_OF_ION_TRANSPORT | 1.591 | <0.001 | <0.001 | <0.001 |
| 262 | GOBP_EXTRACELLULAR_MATRIX_DISASSEMBLY | 1.846 | <0.001 | <0.001 | <0.001 |
| 263 | GOBP_PLATELET_ACTIVATION | 1.728 | <0.001 | <0.001 | <0.001 |
| 264 | HP_PNEUMOTHORAX | 1.840 | <0.001 | <0.001 | <0.001 |
| 265 | HP_ABNORMALITY_OF_HAND_JOINT_MOBILITY | 1.868 | <0.001 | <0.001 | <0.001 |
| 266 | GOBP_POSITIVE_REGULATION_OF_CELL_SUBSTRATE_ADHESION | 1.724 | <0.001 | <0.001 | <0.001 |
| 267 | HP_EMG_ABNORMALITY | 1.605 | <0.001 | <0.001 | <0.001 |
| 268 | HP_ARTERIOSCLEROSIS | 1.808 | <0.001 | <0.001 | <0.001 |
| 269 | HP_SKELETAL_DYSPLASIA | 1.664 | <0.001 | <0.001 | <0.001 |
| 270 | GOBP_SMALL_GTPASE_MEDIATED_SIGNAL_TRANSDUCTION | 1.457 | <0.001 | <0.001 | <0.001 |
| 271 | HP_UNUSUAL_INFECTION_BY_ANATOMICAL_SITE | 1.765 | <0.001 | <0.001 | <0.001 |
| 272 | GOBP_MESENCHYMAL_CELL_DIFFERENTIATION | 1.603 | <0.001 | <0.001 | <0.001 |
| 273 | GOCC_PLATELET_ALPHA_GRANULE_LUMEN | 1.849 | <0.001 | <0.001 | <0.001 |
| 274 | HP_TRANSIENT_ISCHEMIC_ATTACK | 1.857 | <0.001 | <0.001 | <0.001 |
| 275 | GOBP_REGULATION_OF_LYMPHOCYTE_DIFFERENTIATION | 1.664 | <0.001 | <0.001 | <0.001 |
| 276 | HP_ABNORMAL_UPPER_LIMB_BONE_MORPHOLOGY | 1.472 | <0.001 | <0.001 | <0.001 |
| 277 | GOBP_REGULATION_OF_CELL_SUBSTRATE_ADHESION | 1.642 | <0.001 | <0.001 | <0.001 |
| 278 | GOBP_REGULATION_OF_MEMBRANE_POTENTIAL | 1.480 | <0.001 | <0.001 | <0.001 |
| 279 | GOBP_SPROUTING_ANGIOGENESIS | 1.685 | <0.001 | <0.001 | <0.001 |
| 280 | GOCC_PLASMA_MEMBRANE_SIGNALING_RECEPTOR_COMPLEX | 1.658 | <0.001 | <0.001 | <0.001 |
| 281 | GOBP_REGULATION_OF_MUSCLE_SYSTEM_PROCESS | 1.597 | <0.001 | <0.001 | <0.001 |
| 282 | GOMF_AMYLOID_BETA_BINDING | 1.794 | <0.001 | <0.001 | <0.001 |
| 283 | GOMF_METALLOPEPTIDASE_ACTIVITY | 1.639 | <0.001 | <0.001 | <0.001 |
| 284 | HP_ABNORMALITY_OF_THE_GINGIVA | 1.630 | <0.001 | <0.001 | <0.001 |
| 285 | GOBP_T_CELL_PROLIFERATION | 1.618 | <0.001 | <0.001 | <0.001 |
| 286 | HP_AORTIC_REGURGITATION | 1.790 | <0.001 | <0.001 | <0.001 |
| 287 | GOBP_REGULATION_OF_CALCIUM_ION_TRANSPORT_INTO_CYTOSOL | 1.756 | <0.001 | <0.001 | <0.001 |
| 288 | GOBP_RESPONSE_TO_INTERLEUKIN_1 | 1.685 | <0.001 | <0.001 | <0.001 |
| 289 | HP_ABNORMALITY_OF_CIRCULATING_ENZYME_LEVEL | 1.551 | <0.001 | <0.001 | <0.001 |
| 290 | HP_ABNORMALITY_OF_THE_METAPHYSIS | 1.623 | <0.001 | <0.001 | <0.001 |
| 291 | HP_ABNORMAL_SCAPULA_MORPHOLOGY | 1.687 | <0.001 | <0.001 | <0.001 |
| 292 | GOBP_POSITIVE_REGULATION_OF_SUPRAMOLECULAR_FIBER_ORGANIZATION | 1.665 | <0.001 | <0.001 | <0.001 |
| 293 | HP_CARDIAC_ARREST | 1.695 | <0.001 | <0.001 | <0.001 |
| 294 | GOBP_NEGATIVE_REGULATION_OF_CYTOKINE_PRODUCTION | 1.523 | <0.001 | <0.001 | <0.001 |
| 295 | GOMF_CYTOKINE_RECEPTOR_ACTIVITY | 1.761 | <0.001 | <0.001 | <0.001 |
| 296 | GOBP_NEGATIVE_REGULATION_OF_TUMOR_NECROSIS_FACTOR_SUPERFAMILY_CYTOKINE_PRODUCTION | 1.808 | <0.001 | <0.001 | <0.001 |
| 297 | GOBP_REGULATION_OF_PEPTIDYL_TYROSINE_PHOSPHORYLATION | 1.583 | <0.001 | <0.001 | <0.001 |
| 298 | GOBP_ORGAN_GROWTH | 1.676 | <0.001 | <0.001 | <0.001 |
| 299 | GOBP_PRODUCTION_OF_MOLECULAR_MEDIATOR_OF_IMMUNE_RESPONSE | 1.620 | <0.001 | <0.001 | <0.001 |
| 300 | GOBP_REGULATION_OF_B_CELL_ACTIVATION | 1.701 | <0.001 | <0.001 | <0.001 |
| 301 | HP_ABNORMALITY_OF_THE_MUSCULATURE_OF_THE_LIMBS | 1.456 | <0.001 | <0.001 | <0.001 |
| 302 | HP_PECTUS_EXCAVATUM | 1.572 | <0.001 | <0.001 | <0.001 |
| 303 | HP_RETROGNATHIA | 1.577 | <0.001 | <0.001 | <0.001 |
| 304 | GOBP_RAS_PROTEIN_SIGNAL_TRANSDUCTION | 1.514 | <0.001 | <0.001 | <0.001 |
| 305 | HP_ABNORMALITY_OF_THE_RIBS | 1.552 | <0.001 | <0.001 | <0.001 |
| 306 | GOBP_TISSUE_MIGRATION | 1.514 | <0.001 | <0.001 | <0.001 |
| 307 | HP_ABNORMALITY_OF_THE_SHOULDER_GIRDLE_MUSCULATURE | 1.708 | <0.001 | <0.001 | <0.001 |
| 308 | HP_CARDIAC_CONDUCTION_ABNORMALITY | 1.703 | <0.001 | <0.001 | <0.001 |
| 309 | GOBP_POSITIVE_REGULATION_OF_KINASE_ACTIVITY | 1.469 | <0.001 | <0.001 | <0.001 |
| 310 | GOBP_NEGATIVE_REGULATION_OF_LYMPHOCYTE_ACTIVATION | 1.661 | <0.001 | <0.001 | <0.001 |
| 311 | GOBP_REGULATION_OF_PROTEIN_BINDING | 1.631 | <0.001 | <0.001 | <0.001 |
| 312 | GOCC_SYNAPTIC_MEMBRANE | 1.501 | <0.001 | <0.001 | <0.001 |
| 313 | GOCC_PERIKARYON | 1.683 | <0.001 | <0.001 | <0.001 |
| 314 | GOBP_MYELOID_LEUKOCYTE_MEDIATED_IMMUNITY | 1.752 | <0.001 | <0.001 | <0.001 |
| 315 | HP_DENTAL_CROWDING | 1.742 | <0.001 | <0.001 | <0.001 |
| 316 | HP_DILATED_CARDIOMYOPATHY | 1.675 | <0.001 | <0.001 | <0.001 |
| 317 | GOCC_PLASMA_MEMBRANE_RAFT | 1.705 | <0.001 | <0.001 | <0.001 |
| 318 | GOBP_POSITIVE_REGULATION_OF_ENDOTHELIAL_CELL_PROLIFERATION | 1.751 | <0.001 | <0.001 | <0.001 |
| 319 | GOBP_ACTOMYOSIN_STRUCTURE_ORGANIZATION | 1.626 | <0.001 | <0.001 | <0.001 |
| 320 | HP_DYSPNEA | 1.499 | <0.001 | <0.001 | <0.001 |
| 321 | GOBP_ACTIN_FILAMENT_BASED_MOVEMENT | 1.690 | <0.001 | <0.001 | <0.001 |
| 322 | GOBP_REGULATION_OF_CELL_MORPHOGENESIS | 1.533 | <0.001 | <0.001 | <0.001 |
| 323 | GOBP_RESPONSE_TO_TRANSFORMING_GROWTH_FACTOR_BETA | 1.582 | <0.001 | <0.001 | <0.001 |
| 324 | GOBP_CARDIOCYTE_DIFFERENTIATION | 1.672 | <0.001 | <0.001 | <0.001 |
| 325 | GOBP_SMOOTH_MUSCLE_CONTRACTION | 1.736 | <0.001 | <0.001 | <0.001 |
| 326 | HP_VENTRICULAR_ARRHYTHMIA | 1.646 | <0.001 | <0.001 | <0.001 |
| 327 | GOBP_NEGATIVE_REGULATION_OF_CANONICAL_WNT_SIGNALING_PATHWAY | 1.671 | <0.001 | <0.001 | <0.001 |
| 328 | GOBP_POSITIVE_REGULATION_OF_PHOSPHATIDYLINOSITOL_3_KINASE_SIGNALING | 1.790 | <0.001 | <0.001 | <0.001 |
| 329 | GOBP_POSITIVE_REGULATION_OF_PROTEIN_KINASE_ACTIVITY | 1.483 | <0.001 | <0.001 | <0.001 |
| 330 | GOBP_REGULATION_OF_CALCIUM_ION_TRANSMEMBRANE_TRANSPORT | 1.670 | <0.001 | <0.001 | <0.001 |
| 331 | HP_ABNORMAL_CRANIAL_NERVE_PHYSIOLOGY | 1.519 | <0.001 | <0.001 | <0.001 |
| 332 | HP_LIMITED_ELBOW_MOVEMENT | 1.697 | <0.001 | <0.001 | <0.001 |
| 333 | HP_BOWING_OF_THE_LEGS | 1.596 | <0.001 | <0.001 | <0.001 |
| 334 | HP_ELBOW_FLEXION_CONTRACTURE | 1.770 | <0.001 | <0.001 | <0.001 |
| 335 | GOBP_POSITIVE_REGULATION_OF_EPITHELIAL_CELL_PROLIFERATION | 1.593 | <0.001 | <0.001 | <0.001 |
| 336 | HP_ABNORMALITY_OF_PULMONARY_CIRCULATION | 1.667 | <0.001 | <0.001 | <0.001 |
| 337 | GOBP_ADENYLATE_CYCLASE_INHIBITING_G_PROTEIN_COUPLED_RECEPTOR_SIGNALING_PATHWAY | 1.812 | <0.001 | <0.001 | <0.001 |
| 338 | HP_SOMATIC_SENSORY_DYSFUNCTION | 1.505 | <0.001 | <0.001 | <0.001 |
| 339 | HP_ABNORMAL_LYMPHOCYTE_MORPHOLOGY | 1.641 | <0.001 | <0.001 | <0.001 |
| 340 | GOBP_REGULATION_OF_CELL_SHAPE | 1.665 | <0.001 | <0.001 | <0.001 |
| 341 | HP_ABNORMALLY_LAX_OR_HYPEREXTENSIBLE_SKIN | 1.632 | <0.001 | <0.001 | <0.001 |
| 342 | GOBP_LYMPHOCYTE_ACTIVATION_INVOLVED_IN_IMMUNE_RESPONSE | 1.623 | <0.001 | <0.001 | <0.001 |
| 343 | HP_ABNORMAL_RIB_CAGE_MORPHOLOGY | 1.500 | <0.001 | <0.001 | <0.001 |
| 344 | HP_DISPROPORTIONATE_SHORT_STATURE | 1.687 | <0.001 | <0.001 | <0.001 |
| 345 | GOBP_ACTIN_MEDIATED_CELL_CONTRACTION | 1.724 | <0.001 | <0.001 | <0.001 |
| 346 | GOBP_REGULATION_OF_MONONUCLEAR_CELL_MIGRATION | 1.707 | <0.001 | <0.001 | <0.001 |
| 347 | HP_APLASIA_HYPOPLASIA_INVOLVING_BONES_OF_THE_LOWER_LIMBS | 1.487 | <0.001 | <0.001 | <0.001 |
| 348 | GOBP_APOPTOTIC_CELL_CLEARANCE | 1.811 | <0.001 | <0.001 | <0.001 |
| 349 | GOMF_HORMONE_ACTIVITY | 1.692 | <0.001 | <0.001 | <0.001 |
| 350 | HP_PES_VALGUS | 1.815 | <0.001 | <0.001 | <0.001 |
| 351 | GOBP_REGULATION_OF_CANONICAL_WNT_SIGNALING_PATHWAY | 1.574 | <0.001 | <0.001 | <0.001 |
| 352 | GOBP_REGULATION_OF_NEURON_PROJECTION_DEVELOPMENT | 1.456 | <0.001 | <0.001 | <0.001 |
| 353 | GOBP_CANONICAL_WNT_SIGNALING_PATHWAY | 1.510 | <0.001 | <0.001 | <0.001 |
| 354 | HP_STROKE | 1.653 | <0.001 | <0.001 | <0.001 |
| 355 | GOBP_B_CELL_PROLIFERATION | 1.735 | <0.001 | <0.001 | <0.001 |
| 356 | GOBP_BONE_MORPHOGENESIS | 1.727 | <0.001 | <0.001 | <0.001 |
| 357 | HP_KNEE_FLEXION_CONTRACTURE | 1.788 | <0.001 | <0.001 | <0.001 |
| 358 | GOBP_REGULATION_OF_LEUKOCYTE_MEDIATED_IMMUNITY | 1.569 | <0.001 | <0.001 | <0.001 |
| 359 | HP_DESCENDING_AORTIC_DISSECTION | 1.779 | <0.001 | <0.001 | <0.001 |
| 360 | GOBP_REGULATION_OF_NEUTROPHIL_MIGRATION | 1.788 | <0.001 | <0.001 | <0.001 |
| 361 | HP_ABNORMAL_SYSTEMIC_BLOOD_PRESSURE | 1.450 | <0.001 | <0.001 | <0.001 |
| 362 | HP_MYOPATHY | 1.516 | <0.001 | <0.001 | <0.001 |
| 363 | GOBP_MYELOID_CELL_ACTIVATION_INVOLVED_IN_IMMUNE_RESPONSE | 1.714 | <0.001 | <0.001 | <0.001 |
| 364 | HP_ABNORMAL_PERICARDIUM_MORPHOLOGY | 1.787 | <0.001 | <0.001 | <0.001 |
| 365 | HP_INCREASED_SUSCEPTIBILITY_TO_FRACTURES | 1.576 | <0.001 | <0.001 | <0.001 |
| 366 | GOCC_VESICLE_LUMEN | 1.512 | <0.001 | <0.001 | <0.001 |
| 367 | GOBP_ANTIGEN_RECEPTOR_MEDIATED_SIGNALING_PATHWAY | 1.616 | <0.001 | <0.001 | <0.001 |
| 368 | GOBP_NEGATIVE_REGULATION_OF_CELL_GROWTH | 1.610 | <0.001 | <0.001 | <0.001 |
| 369 | HP_FUNCTIONAL_MOTOR_DEFICIT | 1.433 | <0.001 | <0.001 | <0.001 |
| 370 | HP_ABNORMAL_ATRIOVENTRICULAR_CONDUCTION | 1.762 | <0.001 | <0.001 | <0.001 |
| 371 | HP_ABNORMAL_MYELOID_LEUKOCYTE_MORPHOLOGY | 1.509 | <0.001 | <0.001 | <0.001 |
| 372 | HP_ABNORMAL_AORTIC_VALVE_CUSP_MORPHOLOGY | 1.706 | <0.001 | <0.001 | <0.001 |
| 373 | GOBP_POSITIVE_REGULATION_OF_CYTOSKELETON_ORGANIZATION | 1.604 | <0.001 | <0.001 | <0.001 |
| 374 | HP_SUBARACHNOID_HEMORRHAGE | 1.827 | <0.001 | <0.001 | <0.001 |
| 375 | GOCC_BLOOD_MICROPARTICLE | 1.684 | <0.001 | <0.001 | <0.001 |
| 376 | GOBP_REGULATION_OF_PROTEIN_CONTAINING_COMPLEX_ASSEMBLY | 1.457 | <0.001 | <0.001 | <0.001 |
| 377 | GOCC_NEURON_SPINE | 1.638 | <0.001 | <0.001 | <0.001 |
| 378 | GOBP_NEGATIVE_REGULATION_OF_GROWTH | 1.546 | <0.001 | <0.001 | <0.001 |
| 379 | GOBP_REGULATION_OF_PHOSPHATIDYLINOSITOL_3_KINASE_SIGNALING | 1.701 | <0.001 | <0.001 | <0.001 |
| 380 | HP_ABNORMALITY_OF_CRANIAL_SUTURES | 1.498 | <0.001 | <0.001 | <0.001 |
| 381 | GOMF_GROWTH_FACTOR_ACTIVITY | 1.647 | <0.001 | <0.001 | <0.001 |
| 382 | GOBP_EPITHELIAL_CELL_PROLIFERATION | 1.447 | <0.001 | <0.001 | <0.001 |
| 383 | HP_LYMPHOPENIA | 1.656 | <0.001 | <0.001 | <0.001 |
| 384 | HP_LIMB_MUSCLE_WEAKNESS | 1.548 | <0.001 | <0.001 | <0.001 |
| 385 | GOBP_GASTRULATION | 1.603 | <0.001 | <0.001 | <0.001 |
| 386 | GOBP_MICROGLIAL_CELL_ACTIVATION | 1.775 | <0.001 | <0.001 | <0.001 |
| 387 | GOBP_CELLULAR_DEFENSE_RESPONSE | 1.794 | <0.001 | <0.001 | <0.001 |
| 388 | HP_CUTIS_MARMORATA | 1.793 | <0.001 | <0.001 | <0.001 |
| 389 | GOMF_PROTEASE_BINDING | 1.631 | <0.001 | <0.001 | <0.001 |
| 390 | GOBP_ALPHA_BETA_T_CELL_ACTIVATION | 1.637 | <0.001 | <0.001 | <0.001 |
| 391 | GOBP_REGULATION_OF_SYNAPTIC_PLASTICITY | 1.583 | <0.001 | <0.001 | <0.001 |
| 392 | HP_HEART_BLOCK | 1.706 | <0.001 | <0.001 | <0.001 |
| 393 | HP_ISCHEMIC_STROKE | 1.809 | <0.001 | <0.001 | <0.001 |
| 394 | GOBP_TISSUE_REMODELING | 1.619 | <0.001 | <0.001 | <0.001 |
| 395 | HP_SHOULDER_DISLOCATION | 1.805 | <0.001 | <0.001 | <0.001 |
| 396 | GOBP_REGULATION_OF_TUBE_SIZE | 1.653 | <0.001 | <0.001 | <0.001 |
| 397 | HP_ARTHRITIS | 1.589 | <0.001 | <0.001 | <0.001 |
| 398 | GOBP_MUSCLE_ORGAN_MORPHOGENESIS | 1.750 | <0.001 | <0.001 | <0.001 |
| 399 | GOBP_REGULATION_OF_MUSCLE_CELL_DIFFERENTIATION | 1.665 | <0.001 | <0.001 | <0.001 |
| 400 | HP_LIMB_UNDERGROWTH | 1.568 | <0.001 | <0.001 | <0.001 |
| 401 | HP_PERIPHERAL_ARTERIAL_STENOSIS | 1.805 | <0.001 | <0.001 | <0.001 |
| 402 | GOBP_PHOSPHOLIPASE_C_ACTIVATING_G_PROTEIN_COUPLED_RECEPTOR_SIGNALING_PATHWAY | 1.702 | <0.001 | <0.001 | <0.001 |
| 403 | HP_ABNORMALITY_OF_THE_PULMONARY_ARTERY | 1.604 | <0.001 | <0.001 | <0.001 |
| 404 | GOCC_CELL_LEADING_EDGE | 1.444 | <0.001 | <0.001 | <0.001 |
| 405 | GOBP_EOSINOPHIL_MIGRATION | 1.802 | <0.001 | <0.001 | <0.001 |
| 406 | GOCC_DISTAL_AXON | 1.531 | <0.001 | <0.001 | <0.001 |
| 407 | GOBP_RESPONSE_TO_INTERFERON_GAMMA | 1.630 | <0.001 | <0.001 | <0.001 |
| 408 | HP_ABNORMAL_SHOULDER_PHYSIOLOGY | 1.767 | <0.001 | <0.001 | <0.001 |
| 409 | GOBP_PROTEIN_KINASE_B_SIGNALING | 1.563 | <0.001 | <0.001 | <0.001 |
| 410 | HP_ABNORMALITY_OF_THE_MUSCULATURE_OF_THE_LOWER_LIMBS | 1.700 | <0.001 | <0.001 | <0.001 |
| 411 | GOBP_POSITIVE_REGULATION_OF_PEPTIDYL_TYROSINE_PHOSPHORYLATION | 1.593 | <0.001 | <0.001 | <0.001 |
| 412 | GOBP_MULTICELLULAR_ORGANISMAL_SIGNALING | 1.626 | <0.001 | <0.001 | <0.001 |
| 413 | HP_ABNORMAL_RIGHT_VENTRICLE_MORPHOLOGY | 1.790 | <0.001 | <0.001 | <0.001 |
| 414 | GOBP_POSITIVE_REGULATION_OF_ENDOCYTOSIS | 1.708 | <0.001 | <0.001 | <0.001 |
| 415 | HP_WEAKNESS_OF_FACIAL_MUSCULATURE | 1.543 | <0.001 | <0.001 | <0.001 |
| 416 | GOBP_IMMUNOGLOBULIN_PRODUCTION | 1.651 | <0.001 | <0.001 | <0.001 |
| 417 | GOBP_REGULATION_OF_T_CELL_PROLIFERATION | 1.626 | <0.001 | <0.001 | <0.001 |
| 418 | HP_ABNORMAL_PARANASAL_SINUS_MORPHOLOGY | 1.624 | <0.001 | <0.001 | <0.001 |
| 419 | HP_EXERTIONAL_DYSPNEA | 1.693 | <0.001 | <0.001 | <0.001 |
| 420 | GOBP_SKELETAL_MUSCLE_ORGAN_DEVELOPMENT | 1.614 | <0.001 | <0.001 | <0.001 |
| 421 | GOMF_PROTEOGLYCAN_BINDING | 1.824 | <0.001 | <0.001 | <0.001 |
| 422 | HP_AXIAL_MUSCLE_WEAKNESS | 1.791 | <0.001 | <0.001 | <0.001 |
| 423 | GOCC_VACUOLAR_LUMEN | 1.605 | <0.001 | <0.001 | <0.001 |
| 424 | HP_VARIABLE_EXPRESSIVITY | 1.517 | <0.001 | <0.001 | <0.001 |
| 425 | GOBP_CELLULAR_COMPONENT_ASSEMBLY_INVOLVED_IN_MORPHOGENESIS | 1.676 | <0.001 | <0.001 | <0.001 |
| 426 | HP_BROAD_LONG_BONES | 1.615 | <0.001 | <0.001 | <0.001 |
| 427 | GOBP_EOSINOPHIL_CHEMOTAXIS | 1.777 | <0.001 | <0.001 | <0.001 |
| 428 | HP_ABNORMAL_SUBARACHNOID_SPACE_MORPHOLOGY | 1.646 | <0.001 | <0.001 | <0.001 |
| 429 | HP_TALIPES_EQUINOVARUS | 1.495 | <0.001 | <0.001 | <0.001 |
| 430 | HP_SOFT_SKIN | 1.819 | <0.001 | <0.001 | <0.001 |
| 431 | GOCC_COMPLEX_OF_COLLAGEN_TRIMERS | 1.796 | <0.001 | <0.001 | <0.001 |
| 432 | GOBP_NEGATIVE_REGULATION_OF_LEUKOCYTE_MIGRATION | 1.778 | <0.001 | <0.001 | <0.001 |
| 433 | HP_FOOT_DORSIFLEXOR_WEAKNESS | 1.720 | <0.001 | <0.001 | <0.001 |
| 434 | HP_SUDDEN_DEATH | 1.667 | <0.001 | <0.001 | <0.001 |
| 435 | GOBP_LYMPHOCYTE_CHEMOTAXIS | 1.770 | <0.001 | <0.001 | <0.001 |
| 436 | HP_TISSUE_ISCHEMIA | 1.641 | <0.001 | <0.001 | <0.001 |
| 437 | HP_VASCULITIS | 1.774 | <0.001 | <0.001 | <0.001 |
| 438 | GOBP_SYNAPSE_ORGANIZATION | 1.433 | <0.001 | <0.001 | <0.001 |
| 439 | GOBP_ACUTE_INFLAMMATORY_RESPONSE | 1.676 | <0.001 | <0.001 | <0.001 |
| 440 | HP_SYNCOPE | 1.682 | <0.001 | <0.001 | <0.001 |
| 441 | GOBP_CELL_ADHESION_MEDIATED_BY_INTEGRIN | 1.719 | <0.001 | <0.001 | <0.001 |
| 442 | HP_ABNORMALITY_OF_THE_ANKLES | 1.625 | <0.001 | <0.001 | <0.001 |
| 443 | GOBP_REGULATION_OF_EXTRACELLULAR_MATRIX_ORGANIZATION | 1.772 | <0.001 | <0.001 | <0.001 |
| 444 | GOBP_GLIOGENESIS | 1.503 | <0.001 | <0.001 | <0.001 |
| 445 | GOBP_REGULATION_OF_PROTEIN_SERINE_THREONINE_KINASE_ACTIVITY | 1.457 | <0.001 | <0.001 | <0.001 |
| 446 | HP_ADULT_ONSET | 1.533 | <0.001 | <0.001 | <0.001 |
| 447 | GOCC_ACTIN_FILAMENT | 1.667 | <0.001 | <0.001 | <0.001 |
| 448 | GOBP_STRIATED_MUSCLE_CONTRACTION | 1.600 | <0.001 | <0.001 | <0.001 |
| 449 | HP_MUSCULAR_DYSTROPHY | 1.752 | <0.001 | <0.001 | <0.001 |
| 450 | GOBP_NEGATIVE_REGULATION_OF_CELLULAR_RESPONSE_TO_GROWTH_FACTOR_STIMULUS | 1.682 | <0.001 | <0.001 | <0.001 |
| 451 | HP_POOR_WOUND_HEALING | 1.792 | <0.001 | <0.001 | <0.001 |
| 452 | HP_FLEXION_CONTRACTURE_OF_DIGIT | 1.471 | <0.001 | <0.001 | <0.001 |
| 453 | GOBP_NEGATIVE_REGULATION_OF_PEPTIDASE_ACTIVITY | 1.518 | <0.001 | <0.001 | <0.001 |
| 454 | GOBP_NEGATIVE_REGULATION_OF_INFLAMMATORY_RESPONSE | 1.606 | <0.001 | <0.001 | <0.001 |
| 455 | GOBP_REGULATED_EXOCYTOSIS | 1.535 | <0.001 | 0.001 | <0.001 |
| 456 | GOBP_INTERFERON_GAMMA_PRODUCTION | 1.675 | <0.001 | 0.001 | <0.001 |
| 457 | GOBP_NEGATIVE_REGULATION_OF_HYDROLASE_ACTIVITY | 1.463 | <0.001 | 0.001 | <0.001 |
| 458 | GOBP_POSITIVE_REGULATION_OF_LYMPHOCYTE_DIFFERENTIATION | 1.686 | <0.001 | 0.001 | <0.001 |
| 459 | GOBP_NEGATIVE_REGULATION_OF_SMOOTH_MUSCLE_CELL_PROLIFERATION | 1.770 | <0.001 | 0.001 | <0.001 |
| 460 | GOBP_POSITIVE_REGULATION_OF_T_CELL_PROLIFERATION | 1.676 | <0.001 | 0.001 | <0.001 |
| 461 | HP_PURPURA | 1.673 | <0.001 | 0.001 | <0.001 |
| 462 | HP_MIDFACE_RETRUSION | 1.518 | <0.001 | 0.001 | <0.001 |
| 463 | HP_ABNORMALITY_OF_THE_WRIST | 1.627 | <0.001 | 0.001 | <0.001 |
| 464 | HP_AORTIC_DISSECTION | 1.772 | <0.001 | 0.001 | <0.001 |
| 465 | HP_ABNORMAL_FEMORAL_NECK_MORPHOLOGY | 1.615 | <0.001 | 0.001 | <0.001 |
| 466 | HP_DISPROPORTIONATE_SHORT_LIMB_SHORT_STATURE | 1.685 | <0.001 | 0.001 | <0.001 |
| 467 | HP_CONTRACTURES_OF_THE_JOINTS_OF_THE_UPPER_LIMBS | 1.502 | <0.001 | 0.001 | <0.001 |
| 468 | HP_CIGARETTE_PAPER_SCARS | 1.722 | <0.001 | 0.001 | <0.001 |
| 469 | GOBP_HUMORAL_IMMUNE_RESPONSE_MEDIATED_BY_CIRCULATING_IMMUNOGLOBULIN | 1.755 | <0.001 | 0.001 | <0.001 |
| 470 | HP_ABNORMAL_CORONARY_ARTERY_MORPHOLOGY | 1.767 | <0.001 | 0.001 | <0.001 |
| 471 | GOBP_COMPLEMENT_RECEPTOR_MEDIATED_SIGNALING_PATHWAY | 1.776 | <0.001 | 0.001 | <0.001 |
| 472 | GOBP_LEUKOCYTE_DEGRANULATION | 1.733 | <0.001 | 0.001 | <0.001 |
| 473 | HP_DIARRHEA | 1.471 | <0.001 | 0.001 | <0.001 |
| 474 | GOBP_REGULATION_OF_ADAPTIVE_IMMUNE_RESPONSE | 1.570 | <0.001 | 0.001 | <0.001 |
| 475 | GOBP_NEGATIVE_REGULATION_OF_CELL_ADHESION | 1.499 | <0.001 | 0.001 | <0.001 |
| 476 | GOBP_SENSORY_SYSTEM_DEVELOPMENT | 1.438 | <0.001 | 0.001 | <0.001 |
| 477 | HP_JUVENILE_ONSET | 1.612 | <0.001 | 0.001 | <0.001 |
| 478 | GOMF_CALMODULIN_BINDING | 1.571 | <0.001 | 0.001 | <0.001 |
| 479 | GOCC_EARLY_ENDOSOME | 1.436 | <0.001 | 0.001 | <0.001 |
| 480 | GOMF_PEPTIDASE_REGULATOR_ACTIVITY | 1.547 | <0.001 | 0.001 | <0.001 |
| 481 | GOBP_PLATELET_AGGREGATION | 1.740 | <0.001 | 0.001 | <0.001 |
| 482 | GOBP_CELLULAR_RESPONSE_TO_INTERLEUKIN_1 | 1.673 | <0.001 | 0.001 | <0.001 |
| 483 | GOBP_CELL_CELL_ADHESION_VIA_PLASMA_MEMBRANE_ADHESION_MOLECULES | 1.512 | <0.001 | 0.001 | <0.001 |
| 484 | GOBP_RESPONSE_TO_PEPTIDE | 1.400 | <0.001 | 0.001 | <0.001 |
| 485 | HP_ABNORMAL_LEUKOCYTE_PHYSIOLOGY | 1.505 | <0.001 | 0.001 | <0.001 |
| 486 | GOBP_REGULATION_OF_OSTEOBLAST_DIFFERENTIATION | 1.630 | <0.001 | 0.001 | <0.001 |
| 487 | GOCC_LYSOSOMAL_LUMEN | 1.680 | <0.001 | 0.001 | <0.001 |
| 488 | GOBP_MATURE_B_CELL_DIFFERENTIATION | 1.790 | <0.001 | 0.001 | <0.001 |
| 489 | HP_ABNORMALITY_OF_FEMUR_MORPHOLOGY | 1.517 | <0.001 | 0.001 | <0.001 |
| 490 | GOMF_CHEMOKINE_ACTIVITY | 1.766 | <0.001 | 0.001 | <0.001 |
| 491 | GOBP_REGULATION_OF_PRODUCTION_OF_MOLECULAR_MEDIATOR_OF_IMMUNE_RESPONSE | 1.597 | <0.001 | 0.001 | <0.001 |
| 492 | HP_ABNORMAL_EXTEROCEPTIVE_SENSATION | 1.538 | <0.001 | 0.001 | <0.001 |
| 493 | HP_ABDOMINAL_AORTIC_ANEURYSM | 1.777 | <0.001 | 0.001 | <0.001 |
| 494 | GOBP_RESPONSE_TO_BMP | 1.591 | <0.001 | 0.001 | <0.001 |
| 495 | HP_ABNORMAL_ELASTICITY_OF_SKIN | 1.550 | <0.001 | 0.001 | <0.001 |
| 496 | GOCC_POSTSYNAPTIC_MEMBRANE | 1.504 | <0.001 | 0.001 | 0.001 |
| 497 | HP_GENU_VALGUM | 1.613 | <0.001 | 0.001 | 0.001 |
| 498 | GOMF_COMPLEMENT_RECEPTOR_ACTIVITY | 1.761 | <0.001 | 0.001 | 0.001 |
| 499 | HP_ABNORMAL_SPINAL_MENINGEAL_MORPHOLOGY | 1.775 | <0.001 | 0.001 | 0.001 |
| 500 | GOBP_ARTERY_DEVELOPMENT | 1.663 | <0.001 | 0.001 | 0.001 |
| 501 | HP_OSTEOPENIA | 1.509 | <0.001 | 0.001 | 0.001 |
| 502 | GOMF_ACTIN_FILAMENT_BINDING | 1.543 | <0.001 | 0.001 | 0.001 |
| 503 | GOBP_REGULATION_OF_BMP_SIGNALING_PATHWAY | 1.663 | <0.001 | 0.001 | 0.001 |
| 504 | GOBP_EPITHELIAL_TO_MESENCHYMAL_TRANSITION | 1.611 | <0.001 | 0.001 | 0.001 |
| 505 | GOMF_SERINE_HYDROLASE_ACTIVITY | 1.561 | <0.001 | 0.001 | 0.001 |
| 506 | HP_JOINT_CONTRACTURE_OF_THE_HAND | 1.524 | <0.001 | 0.001 | 0.001 |
| 507 | HP_POSITIONAL_FOOT_DEFORMITY | 1.433 | <0.001 | 0.001 | 0.001 |
| 508 | GOBP_COGNITION | 1.468 | <0.001 | 0.001 | 0.001 |
| 509 | HP_SLOWLY_PROGRESSIVE | 1.608 | <0.001 | 0.001 | 0.001 |
| 510 | GOBP_POSITIVE_REGULATION_OF_LEUKOCYTE_CHEMOTAXIS | 1.686 | <0.001 | 0.001 | 0.001 |
| 511 | HP_PROXIMAL_MUSCLE_WEAKNESS | 1.567 | <0.001 | 0.001 | 0.001 |
| 512 | GOBP_NEGATIVE_REGULATION_OF_PROTEOLYSIS | 1.450 | <0.001 | 0.001 | 0.001 |
| 513 | HP_RECURRENT_PNEUMONIA | 1.670 | <0.001 | 0.001 | 0.001 |
| 514 | HP_LIMB_JOINT_CONTRACTURE | 1.444 | <0.001 | 0.001 | 0.001 |
| 515 | HP_ABNORMAL_UMBILICUS_MORPHOLOGY | 1.501 | <0.001 | 0.001 | 0.001 |
| 516 | GOBP_REGULATION_OF_BINDING | 1.439 | <0.001 | 0.001 | 0.001 |
| 517 | HP_CORONARY_ARTERY_ATHEROSCLEROSIS | 1.744 | <0.001 | 0.001 | 0.001 |
| 518 | GOBP_REGULATION_OF_ACUTE_INFLAMMATORY_RESPONSE | 1.744 | <0.001 | 0.001 | 0.001 |
| 519 | HP_QUADRICEPS_MUSCLE_WEAKNESS | 1.739 | <0.001 | 0.001 | 0.001 |
| 520 | HP_ASCENDING_AORTIC_DISSECTION | 1.769 | <0.001 | 0.001 | 0.001 |
| 521 | GOBP_GLOMERULUS_DEVELOPMENT | 1.751 | <0.001 | 0.001 | 0.001 |
| 522 | GOCC_ACTIN_FILAMENT_BUNDLE | 1.714 | <0.001 | 0.001 | 0.001 |
| 523 | GOCC_LAMELLIPODIUM | 1.560 | <0.001 | 0.001 | 0.001 |
| 524 | GOBP_REGULATION_OF_RELEASE_OF_SEQUESTERED_CALCIUM_ION_INTO_CYTOSOL | 1.727 | <0.001 | 0.001 | 0.001 |
| 525 | HP_JOINT_LAXITY | 1.527 | <0.001 | 0.001 | 0.001 |
| 526 | GOMF_METALLOENDOPEPTIDASE_ACTIVITY | 1.646 | <0.001 | 0.001 | 0.001 |
| 527 | GOBP_CARDIAC_MUSCLE_CONTRACTION | 1.597 | <0.001 | 0.001 | 0.001 |
| 528 | HP_TALL_STATURE | 1.537 | <0.001 | 0.001 | 0.001 |
| 529 | GOBP_REGULATION_OF_CATION_CHANNEL_ACTIVITY | 1.579 | <0.001 | 0.001 | 0.001 |
| 530 | HP_SHORT_LONG_BONE | 1.558 | <0.001 | 0.001 | 0.001 |
| 531 | HP_CONGESTIVE_HEART_FAILURE | 1.528 | <0.001 | 0.001 | 0.001 |
| 532 | GOBP_ADENYLATE_CYCLASE_ACTIVATING_G_PROTEIN_COUPLED_RECEPTOR_SIGNALING_PATHWAY | 1.590 | <0.001 | 0.001 | 0.001 |
| 533 | GOBP_POSITIVE_REGULATION_OF_PROTEIN_BINDING | 1.683 | <0.001 | 0.001 | 0.001 |
| 534 | HP_RECURRENT_LOWER_RESPIRATORY_TRACT_INFECTIONS | 1.605 | <0.001 | 0.001 | 0.001 |
| 535 | GOBP_NEGATIVE_REGULATION_OF_BONE_REMODELING | 1.750 | <0.001 | 0.001 | 0.001 |
| 536 | GOBP_REGULATION_OF_ENDOCYTOSIS | 1.535 | <0.001 | 0.001 | 0.001 |
| 537 | GOBP_UROGENITAL_SYSTEM_DEVELOPMENT | 1.447 | <0.001 | 0.001 | 0.001 |
| 538 | HP_ABNORMALITY_OF_THE_METACARPAL_BONES | 1.541 | <0.001 | 0.001 | 0.001 |
| 539 | GOCC_MAIN_AXON | 1.739 | <0.001 | 0.001 | 0.001 |
| 540 | GOBP_POSITIVE_REGULATION_OF_TRANSMEMBRANE_TRANSPORT | 1.534 | <0.001 | 0.001 | 0.001 |
| 541 | GOBP_REGULATION_OF_GTPASE_ACTIVITY | 1.448 | <0.001 | 0.001 | 0.001 |
| 542 | GOBP_NEUROINFLAMMATORY_RESPONSE | 1.761 | <0.001 | 0.001 | 0.001 |
| 543 | GOBP_PHAGOCYTOSIS_RECOGNITION | 1.767 | <0.001 | 0.001 | 0.001 |
| 544 | GOBP_NEGATIVE_REGULATION_OF_INTERLEUKIN_12_PRODUCTION | 1.747 | <0.001 | 0.001 | 0.001 |
| 545 | GOBP_RESPONSE_TO_TUMOR_NECROSIS_FACTOR | 1.509 | <0.001 | 0.001 | 0.001 |
| 546 | GOBP_INOSITOL_LIPID_MEDIATED_SIGNALING | 1.568 | <0.001 | 0.001 | 0.001 |
| 547 | GOBP_REGULATION_OF_GRANULOCYTE_CHEMOTAXIS | 1.729 | <0.001 | 0.001 | 0.001 |
| 548 | HP_CONTRACTURES_OF_THE_JOINTS_OF_THE_LOWER_LIMBS | 1.573 | <0.001 | 0.001 | 0.001 |
| 549 | HP_ABNORMAL_DELIVERY | 1.671 | <0.001 | 0.001 | 0.001 |
| 550 | GOBP_REGULATION_OF_LEUKOCYTE_APOPTOTIC_PROCESS | 1.689 | <0.001 | 0.001 | 0.001 |
| 551 | HP_METAPHYSEAL_WIDENING | 1.672 | <0.001 | 0.001 | 0.001 |
| 552 | GOBP_POSITIVE_REGULATION_OF_CELLULAR_COMPONENT_BIOGENESIS | 1.385 | <0.001 | 0.001 | 0.001 |
| 553 | GOBP_REGENERATION | 1.551 | <0.001 | 0.001 | 0.001 |
| 554 | GOCC_PHAGOCYTIC_VESICLE | 1.620 | <0.001 | 0.001 | 0.001 |
| 555 | GOBP_HEART_GROWTH | 1.658 | <0.001 | 0.001 | 0.001 |
| 556 | HP_APLASIA_HYPOPLASIA_OF_THE_SKIN | 1.560 | <0.001 | 0.001 | 0.001 |
| 557 | HP_HIATUS_HERNIA | 1.762 | <0.001 | 0.001 | 0.001 |
| 558 | HP_RIGIDITY | 1.549 | <0.001 | 0.001 | 0.001 |
| 559 | GOMF_CHEMOKINE_RECEPTOR_BINDING | 1.730 | <0.001 | 0.001 | 0.001 |
| 560 | GOBP_VASOCONSTRICTION | 1.691 | <0.001 | 0.001 | 0.001 |
| 561 | GOBP_REGULATION_OF_MACROPHAGE_ACTIVATION | 1.751 | <0.001 | 0.001 | 0.001 |
| 562 | GOBP_NEGATIVE_REGULATION_OF_OSTEOCLAST_DIFFERENTIATION | 1.748 | <0.001 | 0.001 | 0.001 |
| 563 | HP_CELLULITIS | 1.744 | <0.001 | 0.001 | 0.001 |
| 564 | HP_ABNORMAL_MENINGEAL_MORPHOLOGY | 1.608 | <0.001 | 0.001 | 0.001 |
| 565 | GOBP_REGULATION_OF_MUSCLE_CONTRACTION | 1.583 | <0.001 | 0.001 | 0.001 |
| 566 | GOBP_REGULATION_OF_T_CELL_DIFFERENTIATION | 1.587 | <0.001 | 0.001 | 0.001 |
| 567 | HP_OSTEOARTHRITIS | 1.738 | <0.001 | 0.001 | 0.001 |
| 568 | HP_ABNORMALITY_OF_THE_FONTANELLES_OR_CRANIAL_SUTURES | 1.425 | <0.001 | 0.001 | 0.001 |
| 569 | GOBP_MUSCLE_CELL_MIGRATION | 1.670 | <0.001 | 0.001 | 0.001 |
| 570 | GOBP_REGULATION_OF_CELLULAR_EXTRAVASATION | 1.733 | <0.001 | 0.001 | 0.001 |
| 571 | HP_PES_CAVUS | 1.471 | <0.001 | 0.001 | 0.001 |
| 572 | GOBP_OVULATION_CYCLE_PROCESS | 1.735 | <0.001 | 0.001 | 0.001 |
| 573 | HP_MUTISM | 1.748 | <0.001 | 0.001 | 0.001 |
| 574 | GOCC_FICOLIN_1_RICH_GRANULE_MEMBRANE | 1.726 | <0.001 | 0.001 | 0.001 |
| 575 | HP_ABNORMAL_LARGE_INTESTINE_MORPHOLOGY | 1.477 | <0.001 | 0.001 | 0.001 |
| 576 | GOBP_CARDIAC_MUSCLE_CELL_DIFFERENTIATION | 1.632 | <0.001 | 0.001 | 0.001 |
| 577 | HP_ABNORMAL_ACETABULUM_MORPHOLOGY | 1.704 | <0.001 | 0.001 | 0.001 |
| 578 | HP_ABNORMALITY_OF_THE_MUSCULATURE_OF_THE_UPPER_LIMBS | 1.526 | <0.001 | 0.001 | 0.001 |
| 579 | HP_ABNORMAL_THROMBOCYTE_MORPHOLOGY | 1.406 | <0.001 | 0.001 | 0.001 |
| 580 | GOBP_REGULATION_OF_CARTILAGE_DEVELOPMENT | 1.705 | <0.001 | 0.001 | 0.001 |
| 581 | GOBP_VASODILATION | 1.727 | <0.001 | 0.001 | 0.001 |
| 582 | GOMF_CORECEPTOR_ACTIVITY | 1.746 | <0.001 | 0.001 | 0.001 |
| 583 | GOBP_REGULATION_OF_CELLULAR_COMPONENT_SIZE | 1.442 | <0.001 | 0.001 | 0.001 |
| 584 | HP_ELEVATED_PULMONARY_ARTERY_PRESSURE | 1.616 | <0.001 | 0.001 | 0.001 |
| 585 | GOBP_POSITIVE_REGULATION_OF_TUMOR_NECROSIS_FACTOR_SUPERFAMILY_CYTOKINE_PRODUCTION | 1.649 | <0.001 | 0.001 | 0.001 |
| 586 | GOMF_ANTIGEN_BINDING | 1.732 | <0.001 | 0.001 | 0.001 |
| 587 | GOBP_POSITIVE_REGULATION_OF_ION_TRANSMEMBRANE_TRANSPORT | 1.573 | <0.001 | 0.001 | 0.001 |
| 588 | HP_ABNORMAL_CRANIAL_NERVE_MORPHOLOGY | 1.502 | <0.001 | 0.001 | 0.001 |
| 589 | GOBP_REGULATION_OF_HEART_RATE | 1.644 | <0.001 | 0.001 | 0.001 |
| 590 | HP_LONG_TOE | 1.768 | <0.001 | 0.001 | 0.001 |
| 591 | GOBP_T_CELL_MIGRATION | 1.699 | <0.001 | 0.001 | 0.001 |
| 592 | GOBP_POSITIVE_REGULATION_OF_INFLAMMATORY_RESPONSE | 1.580 | <0.001 | 0.001 | 0.001 |
| 593 | GOBP_REGULATION_OF_NEUTROPHIL_CHEMOTAXIS | 1.744 | <0.001 | 0.001 | 0.001 |
| 594 | GOBP_NEGATIVE_REGULATION_OF_TRANSMEMBRANE_TRANSPORT | 1.611 | <0.001 | 0.001 | 0.001 |
| 595 | HP_ABNORMALITY_OF_THE_BLADDER | 1.373 | <0.001 | 0.001 | 0.001 |
| 596 | GOBP_NON_CANONICAL_WNT_SIGNALING_PATHWAY | 1.694 | <0.001 | 0.001 | 0.001 |
| 597 | GOBP_ACTIN_POLYMERIZATION_OR_DEPOLYMERIZATION | 1.539 | <0.001 | 0.001 | 0.001 |
| 598 | GOCC_NEURON_TO_NEURON_SYNAPSE | 1.434 | <0.001 | 0.001 | 0.001 |
| 599 | GOBP_B_CELL_ACTIVATION_INVOLVED_IN_IMMUNE_RESPONSE | 1.672 | <0.001 | 0.001 | 0.001 |
| 600 | GOBP_NEGATIVE_REGULATION_OF_DEFENSE_RESPONSE | 1.493 | <0.001 | 0.001 | 0.001 |
| 601 | GOBP_VASCULAR_ASSOCIATED_SMOOTH_MUSCLE_CELL_PROLIFERATION | 1.681 | <0.001 | 0.001 | 0.001 |
| 602 | HP_TRICUSPID_VALVE_PROLAPSE | 1.736 | <0.001 | 0.001 | 0.001 |
| 603 | GOBP_RESPONSE_TO_ZINC_ION | 1.727 | <0.001 | 0.001 | 0.001 |
| 604 | GOBP_LEUKOCYTE_APOPTOTIC_PROCESS | 1.641 | <0.001 | 0.001 | 0.001 |
| 605 | GOBP_REGULATION_OF_ACTIN_FILAMENT_ORGANIZATION | 1.492 | <0.001 | 0.001 | 0.001 |
| 606 | HP_ABNORMALITY_OF_THE_CALF_MUSCULATURE | 1.725 | <0.001 | 0.001 | 0.001 |
| 607 | GOBP_NEGATIVE_REGULATION_OF_IMMUNE_EFFECTOR_PROCESS | 1.624 | <0.001 | 0.001 | 0.001 |
| 608 | GOBP_COMPLEMENT_ACTIVATION_ALTERNATIVE_PATHWAY | 1.731 | <0.001 | 0.002 | 0.001 |
| 609 | GOBP_LEUKOCYTE_HOMEOSTASIS | 1.673 | <0.001 | 0.002 | 0.001 |
| 610 | HP_BRACHYDACTYLY | 1.433 | <0.001 | 0.002 | 0.001 |
| 611 | HP_GOWERS_SIGN | 1.714 | <0.001 | 0.002 | 0.001 |
| 612 | GOBP_CARDIAC_MUSCLE_CELL_CONTRACTION | 1.688 | <0.001 | 0.002 | 0.001 |
| 613 | HP_ABNORMALITY_OF_BLOOD_VOLUME_HOMEOSTASIS | 1.741 | <0.001 | 0.002 | 0.001 |
| 614 | GOBP_METANEPHRIC_GLOMERULUS_DEVELOPMENT | 1.726 | <0.001 | 0.002 | 0.001 |
| 615 | HP_INTESTINAL_HYPOPLASIA | 1.726 | <0.001 | 0.002 | 0.001 |
| 616 | GOBP_RESPONSE_TO_AMYLOID_BETA | 1.731 | <0.001 | 0.002 | 0.001 |
| 617 | GOBP_COLLAGEN_BIOSYNTHETIC_PROCESS | 1.738 | <0.001 | 0.002 | 0.001 |
| 618 | GOBP_POSITIVE_REGULATION_OF_PROTEIN_KINASE_B_SIGNALING | 1.636 | <0.001 | 0.002 | 0.001 |
| 619 | GOBP_RENAL_SYSTEM_VASCULATURE_DEVELOPMENT | 1.738 | <0.001 | 0.002 | 0.001 |
| 620 | HP_CHEST_PAIN | 1.628 | <0.001 | 0.002 | 0.001 |
| 621 | GOBP_POSITIVE_REGULATION_OF_ORGAN_GROWTH | 1.710 | <0.001 | 0.002 | 0.001 |
| 622 | GOBP_PHOSPHATIDYLINOSITOL_3_KINASE_SIGNALING | 1.606 | <0.001 | 0.002 | 0.001 |
| 623 | HP_ABNORMAL_CARDIAC_TEST | 1.617 | <0.001 | 0.002 | 0.001 |
| 624 | GOBP_ENDODERMAL_CELL_DIFFERENTIATION | 1.702 | <0.001 | 0.002 | 0.001 |
| 625 | HP_LIMB_GIRDLE_MUSCLE_WEAKNESS | 1.654 | <0.001 | 0.002 | 0.001 |
| 626 | GOBP_NEGATIVE_REGULATION_OF_PHOSPHORUS_METABOLIC_PROCESS | 1.381 | <0.001 | 0.002 | 0.001 |
| 627 | HP_ABNORMAL_SHOULDER_MORPHOLOGY | 1.605 | <0.001 | 0.002 | 0.001 |
| 628 | GOBP_NEGATIVE_REGULATION_OF_VASCULATURE_DEVELOPMENT | 1.590 | <0.001 | 0.002 | 0.001 |
| 629 | HP_ABNORMALITY_OF_THE_CHIN | 1.455 | <0.001 | 0.002 | 0.001 |
| 630 | HP_ABNORMALITY_OF_COMPLEMENT_SYSTEM | 1.736 | <0.001 | 0.002 | 0.001 |
| 631 | GOMF_OPSONIN_BINDING | 1.722 | <0.001 | 0.002 | 0.001 |
| 632 | GOBP_FORMATION_OF_PRIMARY_GERM_LAYER | 1.612 | <0.001 | 0.002 | 0.001 |
| 633 | GOCC_SPECIFIC_GRANULE | 1.557 | <0.001 | 0.002 | 0.001 |
| 634 | HP_JOINT_STIFFNESS | 1.531 | <0.001 | 0.002 | 0.001 |
| 635 | HP_DIFFICULTY_WALKING | 1.472 | <0.001 | 0.002 | 0.001 |
| 636 | HP_ABNORMAL_GASTROINTESTINAL_MOTILITY | 1.687 | <0.001 | 0.002 | 0.001 |
| 637 | HP_BRACHYCEPHALY | 1.501 | <0.001 | 0.002 | 0.001 |
| 638 | GOBP_POSITIVE_REGULATION_OF_PROTEIN_CONTAINING_COMPLEX_ASSEMBLY | 1.528 | <0.001 | 0.002 | 0.001 |
| 639 | GOBP_NEGATIVE_REGULATION_OF_NEURON_DEATH | 1.515 | <0.001 | 0.002 | 0.001 |
| 640 | GOBP_EXTRACELLULAR_MATRIX_ASSEMBLY | 1.699 | <0.001 | 0.002 | 0.001 |
| 641 | GOBP_CELL_KILLING | 1.535 | <0.001 | 0.002 | 0.001 |
| 642 | HP_LUMBAR_HYPERLORDOSIS | 1.675 | <0.001 | 0.002 | 0.001 |
| 643 | HP_INCREASED_BLOOD_PRESSURE | 1.416 | <0.001 | 0.002 | 0.001 |
| 644 | HP_GINGIVAL_OVERGROWTH | 1.599 | <0.001 | 0.002 | 0.001 |
| 645 | GOBP_REGULATION_OF_EXTRINSIC_APOPTOTIC_SIGNALING_PATHWAY_VIA_DEATH_DOMAIN_RECEPTORS | 1.706 | <0.001 | 0.002 | 0.001 |
| 646 | GOBP_RESPONSE_TO_AXON_INJURY | 1.674 | <0.001 | 0.002 | 0.001 |
| 647 | HP_ABNORMAL_SPUTUM | 1.613 | <0.001 | 0.002 | 0.001 |
| 648 | HP_ABNORMAL_MUSCLE_FIBER_PROTEIN_EXPRESSION | 1.748 | <0.001 | 0.002 | 0.001 |
| 649 | HP_PNEUMONIA | 1.470 | <0.001 | 0.002 | 0.001 |
| 650 | HP_ABNORMALITY_OF_THE_FOREARM | 1.498 | <0.001 | 0.002 | 0.001 |
| 651 | HP_HEMIPARESIS | 1.678 | <0.001 | 0.002 | 0.001 |
| 652 | GOBP_TRANSFORMING_GROWTH_FACTOR_BETA_RECEPTOR_SIGNALING_PATHWAY | 1.521 | <0.001 | 0.002 | 0.001 |
| 653 | GOBP_POSITIVE_REGULATION_OF_CELL_PROJECTION_ORGANIZATION | 1.423 | <0.001 | 0.002 | 0.001 |
| 654 | GOMF_EXTRACELLULAR_MATRIX_STRUCTURAL_CONSTITUENT_CONFERRING_COMPRESSION_RESISTANCE | 1.744 | <0.001 | 0.002 | 0.001 |
| 655 | GOBP_CELLULAR_COMPONENT_DISASSEMBLY | 1.374 | <0.001 | 0.002 | 0.001 |
| 656 | HP_APLASIA_HYPOPLASIA_INVOLVING_BONES_OF_THE_FEET | 1.456 | <0.001 | 0.002 | 0.001 |
| 657 | HP_FATIGUE | 1.425 | <0.001 | 0.002 | 0.001 |
| 658 | GOCC_ACTOMYOSIN | 1.674 | <0.001 | 0.002 | 0.001 |
| 659 | GOBP_NEGATIVE_REGULATION_OF_HEMOPOIESIS | 1.622 | <0.001 | 0.002 | 0.001 |
| 660 | HP_ARTERIAL_STENOSIS | 1.629 | <0.001 | 0.002 | 0.001 |
| 661 | GOBP_DEFENSE_RESPONSE_TO_BACTERIUM | 1.462 | <0.001 | 0.002 | 0.001 |
| 662 | GOBP_REGULATION_OF_MYELOID_LEUKOCYTE_DIFFERENTIATION | 1.625 | <0.001 | 0.002 | 0.002 |
| 663 | GOBP_REGULATION_OF_MAP_KINASE_ACTIVITY | 1.501 | <0.001 | 0.002 | 0.002 |
| 664 | GOBP_POSITIVE_REGULATION_OF_ORGANELLE_ORGANIZATION | 1.365 | <0.001 | 0.002 | 0.002 |
| 665 | HP_DERMAL_ATROPHY | 1.656 | <0.001 | 0.002 | 0.002 |
| 666 | GOBP_CD8_POSITIVE_ALPHA_BETA_T_CELL_ACTIVATION | 1.740 | <0.001 | 0.002 | 0.002 |
| 667 | HP_ABNORMAL_DENTIN_MORPHOLOGY | 1.717 | <0.001 | 0.002 | 0.002 |
| 668 | GOBP_REGULATION_OF_CELLULAR_RESPONSE_TO_TRANSFORMING_GROWTH_FACTOR_BETA_STIMULUS | 1.581 | <0.001 | 0.002 | 0.002 |
| 669 | HP_SPINAL_RIGIDITY | 1.711 | <0.001 | 0.002 | 0.002 |
| 670 | HP_PROGRESSIVE_MUSCLE_WEAKNESS | 1.703 | <0.001 | 0.002 | 0.002 |
| 671 | GOBP_ENDODERM_FORMATION | 1.708 | <0.001 | 0.002 | 0.002 |
| 672 | HP_ABNORMAL_PERISTALSIS | 1.730 | <0.001 | 0.002 | 0.002 |
| 673 | HP_HEMIPLEGIA_HEMIPARESIS | 1.495 | <0.001 | 0.002 | 0.002 |
| 674 | HP_WRIST_FLEXION_CONTRACTURE | 1.738 | <0.001 | 0.002 | 0.002 |
| 675 | HP_ABNORMALITY_IRIS_MORPHOLOGY | 1.381 | <0.001 | 0.002 | 0.002 |
| 676 | GOBP_CELLULAR_RESPONSE_TO_VASCULAR_ENDOTHELIAL_GROWTH_FACTOR_STIMULUS | 1.689 | <0.001 | 0.002 | 0.002 |
| 677 | GOBP_REGULATION_OF_ANIMAL_ORGAN_MORPHOGENESIS | 1.577 | <0.001 | 0.002 | 0.002 |
| 678 | HP_ABNORMALITY_OF_THE_PERIPHERAL_NERVOUS_SYSTEM | 1.408 | <0.001 | 0.002 | 0.002 |
| 679 | GOBP_CELLULAR_RESPONSE_TO_ZINC_ION | 1.741 | <0.001 | 0.002 | 0.002 |
| 680 | GOBP_REGULATION_OF_TRANSPORTER_ACTIVITY | 1.438 | <0.001 | 0.002 | 0.002 |
| 681 | GOBP_POSITIVE_REGULATION_OF_CALCIUM_ION_TRANSPORT_INTO_CYTOSOL | 1.703 | <0.001 | 0.002 | 0.002 |
| 682 | GOBP_CHONDROCYTE_DEVELOPMENT | 1.717 | <0.001 | 0.002 | 0.002 |
| 683 | GOBP_REGULATION_OF_PHOSPHOLIPASE_C_ACTIVITY | 1.710 | <0.001 | 0.002 | 0.002 |
| 684 | GOBP_REGULATION_OF_ORGAN_GROWTH | 1.640 | <0.001 | 0.002 | 0.002 |
| 685 | GOMF_ENDOPEPTIDASE_REGULATOR_ACTIVITY | 1.515 | <0.001 | 0.002 | 0.002 |
| 686 | HP_ABSCESS | 1.708 | <0.001 | 0.002 | 0.002 |
| 687 | GOBP_INTERLEUKIN_12_PRODUCTION | 1.700 | <0.001 | 0.002 | 0.002 |
| 688 | GOCC_VACUOLAR_MEMBRANE | 1.372 | <0.001 | 0.002 | 0.002 |
| 689 | GOBP_VASCULAR_ASSOCIATED_SMOOTH_MUSCLE_CELL_MIGRATION | 1.739 | <0.001 | 0.003 | 0.002 |
| 690 | GOBP_NEURON_DEATH | 1.405 | <0.001 | 0.003 | 0.002 |
| 691 | HP_ABNORMALITY_OF_FACIAL_MUSCULATURE | 1.418 | <0.001 | 0.003 | 0.002 |
| 692 | HP_HEMOLYTIC_ANEMIA | 1.570 | <0.001 | 0.003 | 0.002 |
| 693 | GOBP_REGULATION_OF_PROTEIN_POLYMERIZATION | 1.504 | <0.001 | 0.003 | 0.002 |
| 694 | GOCC_FILOPODIUM | 1.622 | <0.001 | 0.003 | 0.002 |
| 695 | HP_ABNORMAL_CAROTID_ARTERY_MORPHOLOGY | 1.649 | <0.001 | 0.003 | 0.002 |
| 696 | HP_JOINT_HYPERFLEXIBILITY | 1.508 | <0.001 | 0.003 | 0.002 |
| 697 | GOBP_SMOOTH_MUSCLE_CELL_MIGRATION | 1.686 | <0.001 | 0.003 | 0.002 |
| 698 | HP_ABNORMALITY_OF_THE_HALLUX | 1.504 | <0.001 | 0.003 | 0.002 |
| 699 | GOBP_ANTIGEN_PROCESSING_AND_PRESENTATION | 1.611 | <0.001 | 0.003 | 0.002 |
| 700 | GOBP_NEGATIVE_REGULATION_OF_ION_TRANSPORT | 1.554 | <0.001 | 0.003 | 0.002 |
| 701 | HP_DECREASED_CIRCULATING_ANTIBODY_LEVEL | 1.535 | <0.001 | 0.003 | 0.002 |
| 702 | GOMF_EXTRACELLULAR_MATRIX_BINDING | 1.697 | <0.001 | 0.003 | 0.002 |
| 703 | GOBP_POSITIVE_REGULATION_OF_NERVOUS_SYSTEM_DEVELOPMENT | 1.453 | <0.001 | 0.003 | 0.002 |
| 704 | HP_CONGENITAL_MUSCULAR_DYSTROPHY | 1.684 | <0.001 | 0.003 | 0.002 |
| 705 | HP_LIMITED_HIP_MOVEMENT | 1.697 | <0.001 | 0.003 | 0.002 |
| 706 | GOBP_INTERLEUKIN_1_PRODUCTION | 1.574 | <0.001 | 0.003 | 0.002 |
| 707 | HP_SYSTEMIC_LUPUS_ERYTHEMATOSUS | 1.735 | <0.001 | 0.003 | 0.002 |
| 708 | GOBP_GLOMERULAR_MESANGIUM_DEVELOPMENT | 1.703 | <0.001 | 0.003 | 0.002 |
| 709 | HP_SKIN_RASH | 1.578 | <0.001 | 0.003 | 0.002 |
| 710 | GOCC_TERTIARY_GRANULE | 1.538 | <0.001 | 0.003 | 0.002 |
| 711 | GOBP_REGULATION_OF_NERVOUS_SYSTEM_DEVELOPMENT | 1.367 | <0.001 | 0.003 | 0.002 |
| 712 | GOBP_CELLULAR_RESPONSE_TO_INTERFERON_GAMMA | 1.616 | <0.001 | 0.003 | 0.002 |
| 713 | GOBP_FOREBRAIN_DEVELOPMENT | 1.394 | <0.001 | 0.003 | 0.002 |
| 714 | HP_ABNORMAL_METATARSAL_MORPHOLOGY | 1.579 | <0.001 | 0.003 | 0.002 |
| 715 | GOBP_MESENCHYME_MORPHOGENESIS | 1.700 | <0.001 | 0.003 | 0.002 |
| 716 | GOMF_SIALIC_ACID_BINDING | 1.727 | <0.001 | 0.003 | 0.002 |
| 717 | HP_REDUCED_SYSTOLIC_FUNCTION | 1.731 | <0.001 | 0.003 | 0.002 |
| 718 | GOBP_BONE_REMODELING | 1.644 | <0.001 | 0.003 | 0.002 |
| 719 | GOBP_POSITIVE_REGULATION_OF_WNT_SIGNALING_PATHWAY | 1.579 | <0.001 | 0.003 | 0.002 |
| 720 | GOCC_PRESYNAPTIC_MEMBRANE | 1.578 | <0.001 | 0.003 | 0.002 |
| 721 | HP_ABNORMALITY_OF_THE_CERVICAL_SPINE | 1.388 | <0.001 | 0.003 | 0.002 |
| 722 | GOBP_NEGATIVE_REGULATION_OF_ION_TRANSMEMBRANE_TRANSPORT | 1.615 | <0.001 | 0.003 | 0.002 |
| 723 | HP_NARROW_PALATE | 1.547 | <0.001 | 0.003 | 0.002 |
| 724 | GOBP_NEGATIVE_REGULATION_OF_CELL_CELL_ADHESION | 1.486 | <0.001 | 0.003 | 0.002 |
| 725 | HP_DISTAL_MUSCLE_WEAKNESS | 1.517 | <0.001 | 0.003 | 0.002 |
| 726 | GOBP_CARDIAC_CONDUCTION | 1.618 | <0.001 | 0.003 | 0.002 |
| 727 | GOBP_ENDODERM_DEVELOPMENT | 1.676 | <0.001 | 0.003 | 0.002 |
| 728 | HP_WEAKNESS_OF_MUSCLES_OF_RESPIRATION | 1.613 | <0.001 | 0.003 | 0.002 |
| 729 | GOBP_INTEGRIN_MEDIATED_SIGNALING_PATHWAY | 1.611 | <0.001 | 0.003 | 0.002 |
| 730 | GOBP_POSITIVE_REGULATION_OF_NEUROGENESIS | 1.491 | <0.001 | 0.003 | 0.002 |
| 731 | HP_PAROXYSMAL_ATRIAL_FIBRILLATION | 1.679 | <0.001 | 0.003 | 0.002 |
| 732 | GOBP_ENDOTHELIAL_CELL_MIGRATION | 1.466 | <0.001 | 0.003 | 0.002 |
| 733 | HP_UPPER_EXTREMITY_JOINT_DISLOCATION | 1.642 | <0.001 | 0.003 | 0.002 |
| 734 | GOBP_HEART_VALVE_DEVELOPMENT | 1.674 | <0.001 | 0.003 | 0.002 |
| 735 | HP_NEONATAL_HYPOTONIA | 1.494 | <0.001 | 0.003 | 0.002 |
| 736 | HP_ABNORMALITY_OF_NEUTROPHILS | 1.441 | <0.001 | 0.003 | 0.002 |
| 737 | GOBP_NEGATIVE_REGULATION_OF_LEUKOCYTE_PROLIFERATION | 1.627 | <0.001 | 0.003 | 0.002 |
| 738 | GOBP_REGULATION_OF_EPITHELIAL_CELL_APOPTOTIC_PROCESS | 1.605 | <0.001 | 0.003 | 0.002 |
| 739 | GOBP_NEGATIVE_REGULATION_OF_LEUKOCYTE_CELL_CELL_ADHESION | 1.551 | <0.001 | 0.003 | 0.002 |
| 740 | GOBP_PROTEIN_POLYMERIZATION | 1.445 | <0.001 | 0.003 | 0.002 |
| 741 | GOBP_CARDIAC_CELL_DEVELOPMENT | 1.634 | <0.001 | 0.003 | 0.002 |
| 742 | HP_GENERALIZED_MUSCLE_WEAKNESS | 1.569 | <0.001 | 0.003 | 0.002 |
| 743 | HP_VENTRICULAR_HYPERTROPHY | 1.581 | <0.001 | 0.003 | 0.002 |
| 744 | GOBP_ALPHA_BETA_T_CELL_DIFFERENTIATION | 1.581 | <0.001 | 0.003 | 0.002 |
| 745 | HP_THORACOLUMBAR_SCOLIOSIS | 1.700 | <0.001 | 0.003 | 0.002 |
| 746 | GOBP_POSITIVE_REGULATION_OF_CELLULAR_EXTRAVASATION | 1.697 | <0.001 | 0.003 | 0.002 |
| 747 | HP_ABNORMAL_DISTAL_PHALANX_MORPHOLOGY_OF_FINGER | 1.513 | <0.001 | 0.003 | 0.003 |
| 748 | HP_ABNORMAL_FEMORAL_NECK_HEAD_MORPHOLOGY | 1.522 | <0.001 | 0.003 | 0.003 |
| 749 | HP_PROTRUDING_EAR | 1.493 | <0.001 | 0.003 | 0.003 |
| 750 | GOBP_SYNAPTIC_TRANSMISSION_GLUTAMATERGIC | 1.603 | <0.001 | 0.003 | 0.003 |
| 751 | HP_THORACIC_HYPOPLASIA | 1.537 | <0.001 | 0.003 | 0.003 |
| 752 | GOBP_DEFENSE_RESPONSE_TO_GRAM_NEGATIVE_BACTERIUM | 1.637 | <0.001 | 0.003 | 0.003 |
| 753 | GOBP_IMMUNOLOGICAL_SYNAPSE_FORMATION | 1.721 | <0.001 | 0.003 | 0.003 |
| 754 | GOBP_RESPONSE_TO_ALCOHOL | 1.458 | <0.001 | 0.003 | 0.003 |
| 755 | GOBP_MYELOID_LEUKOCYTE_DIFFERENTIATION | 1.478 | <0.001 | 0.004 | 0.003 |
| 756 | GOBP_POSITIVE_REGULATION_OF_SMOOTH_MUSCLE_CELL_PROLIFERATION | 1.622 | <0.001 | 0.004 | 0.003 |
| 757 | GOBP_NEURON_PROJECTION_GUIDANCE | 1.468 | <0.001 | 0.004 | 0.003 |
| 758 | GOBP_STRESS_RESPONSE_TO_METAL_ION | 1.674 | <0.001 | 0.004 | 0.003 |
| 759 | GOMF_IMMUNOGLOBULIN_BINDING | 1.726 | <0.001 | 0.004 | 0.003 |
| 760 | GOBP_PIGMENT_CELL_DIFFERENTIATION | 1.716 | <0.001 | 0.004 | 0.003 |
| 761 | GOBP_REGULATION_OF_MICROGLIAL_CELL_ACTIVATION | 1.691 | <0.001 | 0.004 | 0.003 |
| 762 | GOBP_STRIATED_MUSCLE_CELL_DEVELOPMENT | 1.673 | <0.001 | 0.004 | 0.003 |
| 763 | HP_RETINAL_DETACHMENT | 1.611 | <0.001 | 0.004 | 0.003 |
| 764 | HP_ANKLE_FLEXION_CONTRACTURE | 1.662 | <0.001 | 0.004 | 0.003 |
| 765 | GOBP_SIGNALING_RECEPTOR_LIGAND_PRECURSOR_PROCESSING | 1.723 | <0.001 | 0.004 | 0.003 |
| 766 | HP_CONGENITAL_HIP_DISLOCATION | 1.665 | <0.001 | 0.004 | 0.003 |
| 767 | GOBP_NEGATIVE_REGULATION_OF_INTERLEUKIN_6_PRODUCTION | 1.680 | <0.001 | 0.004 | 0.003 |
| 768 | HP_ABNORMALITY_OF_THE_CHEEK | 1.487 | <0.001 | 0.004 | 0.003 |
| 769 | GOBP_REGULATION_OF_SYSTEMIC_ARTERIAL_BLOOD_PRESSURE_MEDIATED_BY_A_CHEMICAL_SIGNAL | 1.675 | <0.001 | 0.004 | 0.003 |
| 770 | GOMF_FIBRONECTIN_BINDING | 1.696 | <0.001 | 0.004 | 0.003 |
| 771 | GOBP_ENDOTHELIUM_DEVELOPMENT | 1.526 | <0.001 | 0.004 | 0.003 |
| 772 | GOBP_COLLAGEN_CATABOLIC_PROCESS | 1.700 | <0.001 | 0.004 | 0.003 |
| 773 | GOBP_REGULATION_OF_HUMORAL_IMMUNE_RESPONSE | 1.675 | <0.001 | 0.004 | 0.003 |
| 774 | GOMF_ENDOPEPTIDASE_ACTIVITY | 1.363 | <0.001 | 0.004 | 0.003 |
| 775 | GOMF_ENZYME_INHIBITOR_ACTIVITY | 1.389 | <0.001 | 0.004 | 0.003 |
| 776 | HP_ABNORMALITY_OF_EPIPHYSIS_MORPHOLOGY | 1.455 | <0.001 | 0.004 | 0.003 |
| 777 | HP_PAPULE | 1.626 | <0.001 | 0.004 | 0.003 |
| 778 | HP_ABNORMALITY_OF_FACIAL_SOFT_TISSUE | 1.342 | <0.001 | 0.004 | 0.003 |
| 779 | GOBP_DEVELOPMENTAL_CELL_GROWTH | 1.484 | <0.001 | 0.004 | 0.003 |
| 780 | GOBP_REGULATION_OF_CHONDROCYTE_DIFFERENTIATION | 1.672 | <0.001 | 0.004 | 0.003 |
| 781 | GOCC_TERTIARY_GRANULE_MEMBRANE | 1.644 | <0.001 | 0.004 | 0.003 |
| 782 | HP_DYSPHAGIA | 1.341 | <0.001 | 0.004 | 0.003 |
| 783 | GOBP_FIBROBLAST_PROLIFERATION | 1.632 | <0.001 | 0.004 | 0.003 |
| 784 | HP_ABNORMAL_AUTONOMIC_NERVOUS_SYSTEM_PHYSIOLOGY | 1.559 | <0.001 | 0.004 | 0.003 |
| 785 | HP_IMMUNODEFICIENCY | 1.479 | <0.001 | 0.004 | 0.003 |
| 786 | GOBP_POSITIVE_REGULATION_OF_LEUKOCYTE_MEDIATED_IMMUNITY | 1.528 | <0.001 | 0.004 | 0.003 |
| 787 | HP_TOOTH_MALPOSITION | 1.394 | <0.001 | 0.004 | 0.003 |
| 788 | GOCC_FICOLIN_1_RICH_GRANULE | 1.490 | <0.001 | 0.004 | 0.003 |
| 789 | GOMF_STRUCTURAL_MOLECULE_ACTIVITY_CONFERRING_ELASTICITY | 1.685 | <0.001 | 0.004 | 0.003 |
| 790 | HP_CHILDHOOD_ONSET | 1.512 | <0.001 | 0.004 | 0.003 |
| 791 | HP_ABNORMAL_AORTIC_VALVE_MORPHOLOGY | 1.525 | <0.001 | 0.004 | 0.003 |
| 792 | GOBP_POSITIVE_REGULATION_OF_GTPASE_ACTIVITY | 1.440 | <0.001 | 0.005 | 0.003 |
| 793 | HP_ABNORMAL_TRICUSPID_VALVE_PHYSIOLOGY | 1.663 | <0.001 | 0.005 | 0.003 |
| 794 | GOBP_RESPONSE_TO_KETONE | 1.486 | <0.001 | 0.005 | 0.003 |
| 795 | GOBP_CELL_JUNCTION_ASSEMBLY | 1.357 | <0.001 | 0.005 | 0.003 |
| 796 | GOCC_ANCHORED_COMPONENT_OF_MEMBRANE | 1.517 | <0.001 | 0.005 | 0.003 |
| 797 | GOBP_ACTIN_FILAMENT_POLYMERIZATION | 1.536 | <0.001 | 0.005 | 0.003 |
| 798 | GOBP_NEGATIVE_REGULATION_OF_BIOMINERALIZATION | 1.689 | <0.001 | 0.005 | 0.003 |
| 799 | GOBP_AGING | 1.424 | <0.001 | 0.005 | 0.003 |
| 800 | HP_PACE_OF_PROGRESSION | 1.354 | <0.001 | 0.005 | 0.003 |
| 801 | GOBP_REGULATION_OF_OSTEOCLAST_DIFFERENTIATION | 1.659 | <0.001 | 0.005 | 0.003 |
| 802 | GOBP_RESPONSE_TO_REACTIVE_OXYGEN_SPECIES | 1.490 | <0.001 | 0.005 | 0.004 |
| 803 | GOBP_REGULATION_OF_RESPONSE_TO_BIOTIC_STIMULUS | 1.396 | <0.001 | 0.005 | 0.004 |
| 804 | GOBP_RETINA_VASCULATURE_DEVELOPMENT_IN_CAMERA_TYPE_EYE | 1.663 | <0.001 | 0.005 | 0.004 |
| 805 | GOMF_PLATELET_DERIVED_GROWTH_FACTOR_BINDING | 1.679 | <0.001 | 0.005 | 0.004 |
| 806 | HP_ECTOPIA_LENTIS | 1.674 | <0.001 | 0.005 | 0.004 |
| 807 | GOCC_SPECIFIC_GRANULE_MEMBRANE | 1.609 | <0.001 | 0.005 | 0.004 |
| 808 | HP_NEOPLASM_OF_THE_HEART | 1.710 | <0.001 | 0.005 | 0.004 |
| 809 | GOBP_MELANOCYTE_DIFFERENTIATION | 1.701 | <0.001 | 0.005 | 0.004 |
| 810 | GOMF_BMP_BINDING | 1.679 | <0.001 | 0.005 | 0.004 |
| 811 | GOBP_CELL_MIGRATION_INVOLVED_IN_SPROUTING_ANGIOGENESIS | 1.624 | <0.001 | 0.005 | 0.004 |
| 812 | HP_COXA_VARA | 1.657 | <0.001 | 0.005 | 0.004 |
| 813 | GOBP_REGULATION_OF_IMMUNOGLOBULIN_PRODUCTION | 1.640 | <0.001 | 0.005 | 0.004 |
| 814 | GOBP_INFLAMMATORY_RESPONSE_TO_ANTIGENIC_STIMULUS | 1.645 | <0.001 | 0.005 | 0.004 |
| 815 | HP_ABNORMAL_LEUKOCYTE_COUNT | 1.380 | <0.001 | 0.005 | 0.004 |
| 816 | GOBP_POSITIVE_REGULATION_OF_ALPHA_BETA_T_CELL_ACTIVATION | 1.660 | <0.001 | 0.005 | 0.004 |
| 817 | GOMF_WNT_PROTEIN_BINDING | 1.684 | <0.001 | 0.005 | 0.004 |
| 818 | GOMF_CARGO_RECEPTOR_ACTIVITY | 1.651 | <0.001 | 0.005 | 0.004 |
| 819 | GOCC_NEUROMUSCULAR_JUNCTION | 1.659 | <0.001 | 0.005 | 0.004 |
| 820 | GOBP_ZINC_ION_HOMEOSTASIS | 1.674 | <0.001 | 0.005 | 0.004 |
| 821 | GOBP_POSITIVE_REGULATION_OF_FIBROBLAST_PROLIFERATION | 1.674 | <0.001 | 0.005 | 0.004 |
| 822 | GOBP_NEUROPEPTIDE_SIGNALING_PATHWAY | 1.581 | <0.001 | 0.005 | 0.004 |
| 823 | GOBP_PEPTIDE_ANTIGEN_ASSEMBLY_WITH_MHC_CLASS_II_PROTEIN_COMPLEX | 1.675 | <0.001 | 0.005 | 0.004 |
| 824 | GOBP_REGULATION_OF_EPITHELIAL_CELL_MIGRATION | 1.418 | <0.001 | 0.005 | 0.004 |
| 825 | GOBP_RESPONSE_TO_FIBROBLAST_GROWTH_FACTOR | 1.586 | <0.001 | 0.005 | 0.004 |
| 826 | HP_MACULAR_PURPURA | 1.656 | <0.001 | 0.005 | 0.004 |
| 827 | GOBP_REGULATION_OF_ACTIN_FILAMENT_LENGTH | 1.517 | <0.001 | 0.005 | 0.004 |
| 828 | GOBP_DEVELOPMENTAL_GROWTH_INVOLVED_IN_MORPHOGENESIS | 1.451 | <0.001 | 0.005 | 0.004 |
| 829 | GOBP_REGULATION_OF_PEPTIDASE_ACTIVITY | 1.343 | <0.001 | 0.005 | 0.004 |
| 830 | GOBP_POSITIVE_REGULATION_OF_SYNAPTIC_TRANSMISSION | 1.533 | <0.001 | 0.005 | 0.004 |
| 831 | GOBP_GLIAL_CELL_DIFFERENTIATION | 1.467 | <0.001 | 0.005 | 0.004 |
| 832 | GOBP_PROTEIN_PROCESSING | 1.448 | <0.001 | 0.005 | 0.004 |
| 833 | GOCC_PRESYNAPSE | 1.336 | <0.001 | 0.005 | 0.004 |
| 834 | GOBP_OSTEOCLAST_DIFFERENTIATION | 1.582 | <0.001 | 0.005 | 0.004 |
| 835 | GOBP_RECEPTOR_INTERNALIZATION | 1.564 | <0.001 | 0.005 | 0.004 |
| 836 | HP_LOWER_LIMB_MUSCLE_WEAKNESS | 1.530 | <0.001 | 0.005 | 0.004 |
| 837 | GOMF_GTPASE_ACTIVITY | 1.393 | <0.001 | 0.005 | 0.004 |
| 838 | GOBP_REGULATION_OF_SYSTEMIC_ARTERIAL_BLOOD_PRESSURE_BY_HORMONE | 1.685 | <0.001 | 0.005 | 0.004 |
| 839 | GOBP_REGULATION_OF_VASOCONSTRICTION | 1.661 | <0.001 | 0.005 | 0.004 |
| 840 | GOBP_POSITIVE_REGULATION_OF_CALCIUM_ION_TRANSMEMBRANE_TRANSPORT | 1.645 | <0.001 | 0.005 | 0.004 |
| 841 | HP_CARDIOMEGALY | 1.548 | <0.001 | 0.005 | 0.004 |
| 842 | HP_COMBINED_IMMUNODEFICIENCY | 1.692 | <0.001 | 0.005 | 0.004 |
| 843 | GOCC_PROTEIN_COMPLEX_INVOLVED_IN_CELL_ADHESION | 1.668 | <0.001 | 0.005 | 0.004 |
| 844 | HP_ABNORMAL_MACROPHAGE_MORPHOLOGY | 1.700 | <0.001 | 0.006 | 0.004 |
| 845 | HP_ATROPHIC_SCARS | 1.704 | <0.001 | 0.006 | 0.004 |
| 846 | GOBP_OPSONIZATION | 1.672 | <0.001 | 0.006 | 0.004 |
| 847 | GOBP_DENDRITIC_CELL_CHEMOTAXIS | 1.695 | <0.001 | 0.006 | 0.004 |
| 848 | GOBP_REGULATION_OF_HEART_GROWTH | 1.626 | <0.001 | 0.006 | 0.004 |
| 849 | HP_CONTRACTURES_INVOLVING_THE_JOINTS_OF_THE_FEET | 1.605 | <0.001 | 0.006 | 0.004 |
| 850 | HP_ABNORMALITY_OF_THE_ANTERIOR_FONTANELLE | 1.586 | <0.001 | 0.006 | 0.004 |
| 851 | HP_ABNORMALITY_OF_CORNEAL_SIZE | 1.494 | <0.001 | 0.006 | 0.004 |
| 852 | GOBP_POSITIVE_REGULATION_OF_GROWTH | 1.444 | <0.001 | 0.006 | 0.004 |
| 853 | GOCC_GLUTAMATERGIC_SYNAPSE | 1.392 | <0.001 | 0.006 | 0.004 |
| 854 | HP_FACIAL_ASYMMETRY | 1.530 | <0.001 | 0.006 | 0.004 |
| 855 | GOBP_DENDRITE_ARBORIZATION | 1.659 | <0.001 | 0.006 | 0.004 |
| 856 | GOBP_NEUTROPHIL_HOMEOSTASIS | 1.669 | <0.001 | 0.006 | 0.004 |
| 857 | GOBP_RESPONSE_TO_PROSTAGLANDIN_E | 1.683 | <0.001 | 0.006 | 0.004 |
| 858 | HP_TACHYCARDIA | 1.528 | <0.001 | 0.006 | 0.004 |
| 859 | HP_ABNORMAL_METACARPAL_MORPHOLOGY | 1.506 | <0.001 | 0.006 | 0.004 |
| 860 | GOBP_NEGATIVE_REGULATION_OF_PHOSPHORYLATION | 1.367 | <0.001 | 0.006 | 0.004 |
| 861 | GOBP_NEGATIVE_REGULATION_OF_SMOOTH_MUSCLE_CELL_MIGRATION | 1.677 | <0.001 | 0.006 | 0.004 |
| 862 | GOBP_ACTIN_FILAMENT_BUNDLE_ORGANIZATION | 1.521 | <0.001 | 0.006 | 0.004 |
| 863 | GOBP_ASTROCYTE_DEVELOPMENT | 1.679 | <0.001 | 0.006 | 0.004 |
| 864 | HP_ABNORMAL_CELL_PROLIFERATION | 1.676 | <0.001 | 0.006 | 0.004 |
| 865 | HP_ABNORMAL_FEMALE_REPRODUCTIVE_SYSTEM_PHYSIOLOGY | 1.392 | <0.001 | 0.006 | 0.005 |
| 866 | GOBP_TRANSITION_METAL_ION_HOMEOSTASIS | 1.543 | <0.001 | 0.006 | 0.005 |
| 867 | GOBP_POSTSYNAPTIC_SPECIALIZATION_ORGANIZATION | 1.697 | 0.001 | 0.006 | 0.005 |
| 868 | GOBP_LIPOPOLYSACCHARIDE_MEDIATED_SIGNALING_PATHWAY | 1.646 | 0.001 | 0.006 | 0.005 |
| 869 | GOMF_TUBULIN_BINDING | 1.360 | 0.001 | 0.006 | 0.005 |
| 870 | GOBP_REGULATION_OF_MYELOID_CELL_DIFFERENTIATION | 1.478 | 0.001 | 0.006 | 0.005 |
| 871 | HP_APLASIA_HYPOPLASIA_OF_THE_DISTAL_PHALANGES_OF_THE_HAND | 1.534 | 0.001 | 0.006 | 0.005 |
| 872 | GOBP_ARTERY_MORPHOGENESIS | 1.601 | 0.001 | 0.006 | 0.005 |
| 873 | GOBP_CARDIAC_LEFT_VENTRICLE_MORPHOGENESIS | 1.695 | 0.001 | 0.006 | 0.005 |
| 874 | HP_NEPHRITIS | 1.576 | 0.001 | 0.006 | 0.005 |
| 875 | GOBP_REGULATION_OF_SYNAPTIC_TRANSMISSION_GLUTAMATERGIC | 1.632 | 0.001 | 0.006 | 0.005 |
| 876 | GOBP_AXON_EXTENSION | 1.550 | 0.001 | 0.006 | 0.005 |
| 877 | GOBP_NEGATIVE_REGULATION_OF_VASCULAR_ASSOCIATED_SMOOTH_MUSCLE_CELL_PROLIFERATION | 1.692 | 0.001 | 0.006 | 0.005 |
| 878 | GOBP_INTERLEUKIN_8_PRODUCTION | 1.585 | 0.001 | 0.006 | 0.005 |
| 879 | GOBP_HOMOTYPIC_CELL_CELL_ADHESION | 1.580 | 0.001 | 0.006 | 0.005 |
| 880 | GOBP_REGULATION_OF_MYELOID_LEUKOCYTE_MEDIATED_IMMUNITY | 1.640 | 0.001 | 0.006 | 0.005 |
| 881 | GOBP_RESPONSE_TO_OXYGEN_LEVELS | 1.395 | 0.001 | 0.006 | 0.005 |
| 882 | GOBP_MUCOPOLYSACCHARIDE_METABOLIC_PROCESS | 1.588 | 0.001 | 0.006 | 0.005 |
| 883 | HP_HIP_CONTRACTURE | 1.655 | 0.001 | 0.006 | 0.005 |
| 884 | GOBP_MUSCLE_HYPERTROPHY | 1.568 | 0.001 | 0.006 | 0.005 |
| 885 | GOBP_CELLULAR_TRANSITION_METAL_ION_HOMEOSTASIS | 1.551 | 0.001 | 0.006 | 0.005 |
| 886 | HP_EMG_MYOPATHIC_ABNORMALITIES | 1.550 | 0.001 | 0.007 | 0.005 |
| 887 | HP_PROXIMAL_MUSCLE_WEAKNESS_IN_LOWER_LIMBS | 1.653 | 0.001 | 0.007 | 0.005 |
| 888 | GOBP_DENDRITIC_CELL_ANTIGEN_PROCESSING_AND_PRESENTATION | 1.665 | 0.001 | 0.007 | 0.005 |
| 889 | GOBP_NEGATIVE_REGULATION_OF_IMMUNE_RESPONSE | 1.500 | 0.001 | 0.007 | 0.005 |
| 890 | GOBP_NEURON_MIGRATION | 1.508 | 0.001 | 0.007 | 0.005 |
| 891 | GOBP_ACTIN_CYTOSKELETON_REORGANIZATION | 1.564 | 0.001 | 0.007 | 0.005 |
| 892 | HP_WORMIAN_BONES | 1.654 | 0.001 | 0.007 | 0.005 |
| 893 | GOBP_REGULATION_OF_ANTIGEN_PROCESSING_AND_PRESENTATION | 1.651 | 0.001 | 0.007 | 0.005 |
| 894 | GOBP_DEVELOPMENTAL_MATURATION | 1.394 | 0.001 | 0.007 | 0.005 |
| 895 | GOBP_REGULATION_OF_CELL_KILLING | 1.576 | 0.001 | 0.007 | 0.005 |
| 896 | HP_ABNORMALITY_OF_THE_PALM | 1.321 | 0.001 | 0.007 | 0.005 |
| 897 | GOBP_CARDIAC_MUSCLE_TISSUE_MORPHOGENESIS | 1.636 | 0.001 | 0.007 | 0.005 |
| 898 | GOBP_REGULATION_OF_PROTEIN_MATURATION | 1.643 | 0.001 | 0.007 | 0.005 |
| 899 | GOBP_NEGATIVE_REGULATION_OF_BINDING | 1.488 | 0.001 | 0.007 | 0.005 |
| 900 | GOCC_NEURON_PROJECTION_TERMINUS | 1.528 | 0.001 | 0.007 | 0.005 |
| 901 | GOBP_NEGATIVE_REGULATION_OF_NEURON_APOPTOTIC_PROCESS | 1.515 | 0.001 | 0.007 | 0.005 |
| 902 | GOBP_AORTA_DEVELOPMENT | 1.664 | 0.001 | 0.007 | 0.005 |
| 903 | GOCC_INTRINSIC_COMPONENT_OF_SYNAPTIC_MEMBRANE | 1.496 | 0.001 | 0.007 | 0.005 |
| 904 | GOBP_POSITIVE_REGULATION_OF_PROTEIN_SERINE_THREONINE_KINASE_ACTIVITY | 1.453 | 0.001 | 0.007 | 0.005 |
| 905 | HP_FEVER | 1.393 | 0.001 | 0.007 | 0.005 |
| 906 | GOBP_POSITIVE_REGULATION_OF_EPITHELIAL_TO_MESENCHYMAL_TRANSITION | 1.647 | 0.001 | 0.007 | 0.005 |
| 907 | GOBP_MYELOID_CELL_APOPTOTIC_PROCESS | 1.660 | 0.001 | 0.007 | 0.005 |
| 908 | GOBP_REGULATION_OF_NEUROGENESIS | 1.373 | 0.001 | 0.007 | 0.005 |
| 909 | HP_VERTEBRAL_COMPRESSION_FRACTURE | 1.687 | 0.001 | 0.007 | 0.005 |
| 910 | HP_INTESTINAL_PSEUDO_OBSTRUCTION | 1.662 | 0.001 | 0.007 | 0.005 |
| 911 | GOBP_LEARNING | 1.515 | 0.001 | 0.007 | 0.005 |
| 912 | HP_PERICARDITIS | 1.661 | 0.001 | 0.007 | 0.005 |
| 913 | HP_RECURRENT_BACTERIAL_INFECTIONS | 1.497 | 0.001 | 0.007 | 0.005 |
| 914 | GOBP_POSITIVE_REGULATION_OF_INTERFERON_GAMMA_PRODUCTION | 1.610 | 0.001 | 0.007 | 0.005 |
| 915 | HP_PECTUS_CARINATUM | 1.513 | 0.001 | 0.007 | 0.005 |
| 916 | HP_ABNORMAL_CIRCULATING_IGG_LEVEL | 1.589 | 0.001 | 0.007 | 0.005 |
| 917 | GOBP_AMYLOID_BETA_CLEARANCE | 1.692 | 0.001 | 0.007 | 0.006 |
| 918 | GOBP_REGULATION_OF_SYSTEMIC_ARTERIAL_BLOOD_PRESSURE | 1.579 | 0.001 | 0.007 | 0.006 |
| 919 | GOBP_RENAL_TUBULAR_SECRETION | 1.686 | 0.001 | 0.007 | 0.006 |
| 920 | GOBP_REGULATION_OF_NON_CANONICAL_WNT_SIGNALING_PATHWAY | 1.684 | 0.001 | 0.007 | 0.006 |
| 921 | GOBP_REGULATION_OF_INFLAMMATORY_RESPONSE_TO_ANTIGENIC_STIMULUS | 1.650 | 0.001 | 0.008 | 0.006 |
| 922 | HP_COXA_VALGA | 1.630 | 0.001 | 0.008 | 0.006 |
| 923 | GOBP_DEVELOPMENT_OF_PRIMARY_FEMALE_SEXUAL_CHARACTERISTICS | 1.567 | 0.001 | 0.008 | 0.006 |
| 924 | HP_ABNORMALITY_OF_BLADDER_MORPHOLOGY | 1.620 | 0.001 | 0.008 | 0.006 |
| 925 | GOBP_REGULATION_OF_SMOOTH_MUSCLE_CONTRACTION | 1.635 | 0.001 | 0.008 | 0.006 |
| 926 | GOBP_REGULATION_OF_RENAL_SYSTEM_PROCESS | 1.683 | 0.001 | 0.008 | 0.006 |
| 927 | GOBP_REGULATION_OF_COLLAGEN_METABOLIC_PROCESS | 1.670 | 0.001 | 0.008 | 0.006 |
| 928 | HP_PALPEBRAL_EDEMA | 1.668 | 0.001 | 0.008 | 0.006 |
| 929 | GOBP_POSITIVE_REGULATION_OF_RECEPTOR_MEDIATED_ENDOCYTOSIS | 1.646 | 0.001 | 0.008 | 0.006 |
| 930 | GOBP_NEGATIVE_REGULATION_OF_BMP_SIGNALING_PATHWAY | 1.641 | 0.001 | 0.008 | 0.006 |
| 931 | GOBP_CELL_JUNCTION_DISASSEMBLY | 1.665 | 0.001 | 0.008 | 0.006 |
| 932 | HP_ABNORMAL_MUSCLE_FIBER_MORPHOLOGY | 1.463 | 0.001 | 0.008 | 0.006 |
| 933 | GOBP_NEGATIVE_REGULATION_OF_CARTILAGE_DEVELOPMENT | 1.682 | 0.001 | 0.008 | 0.006 |
| 934 | HP_AREFLEXIA | 1.432 | 0.001 | 0.008 | 0.006 |
| 935 | GOBP_MAST_CELL_ACTIVATION | 1.622 | 0.001 | 0.008 | 0.006 |
| 936 | GOBP_REGULATION_OF_EXTRINSIC_APOPTOTIC_SIGNALING_PATHWAY | 1.497 | 0.001 | 0.008 | 0.006 |
| 937 | GOBP_POSITIVE_REGULATION_OF_RESPONSE_TO_BIOTIC_STIMULUS | 1.496 | 0.001 | 0.008 | 0.006 |
| 938 | HP_ABNORMALITY_OF_SKELETAL_MATURATION | 1.352 | 0.001 | 0.008 | 0.006 |
| 939 | HP_ABNORMAL_TRICUSPID_VALVE_MORPHOLOGY | 1.638 | 0.001 | 0.008 | 0.006 |
| 940 | HP_APLASIA_HYPOPLASIA_OF_THE_PHALANGES_OF_THE_HAND | 1.425 | 0.001 | 0.008 | 0.006 |
| 941 | GOBP_NEGATIVE_REGULATION_OF_TRANSFORMING_GROWTH_FACTOR_BETA_RECEPTOR_SIGNALING_PATHWAY | 1.596 | 0.001 | 0.008 | 0.006 |
| 942 | GOBP_CELL_SUBSTRATE_JUNCTION_ORGANIZATION | 1.572 | 0.001 | 0.008 | 0.006 |
| 943 | GOBP_DETECTION_OF_BIOTIC_STIMULUS | 1.686 | 0.001 | 0.008 | 0.006 |
| 944 | GOBP_POSITIVE_REGULATION_OF_INTERLEUKIN_6_PRODUCTION | 1.569 | 0.001 | 0.008 | 0.006 |
| 945 | GOBP_REGULATION_OF_LYMPHOCYTE_MEDIATED_IMMUNITY | 1.478 | 0.001 | 0.008 | 0.006 |
| 946 | GOBP_CELLULAR_RESPONSE_TO_CADMIUM_ION | 1.684 | 0.001 | 0.008 | 0.006 |
| 947 | HP_ABNORMALITY_OF_THE_UPPER_RESPIRATORY_TRACT | 1.337 | 0.001 | 0.008 | 0.006 |
| 948 | HP_PULMONARY_HEMORRHAGE | 1.648 | 0.001 | 0.008 | 0.006 |
| 949 | GOCC_DYNACTIN_COMPLEX | 1.654 | 0.001 | 0.009 | 0.006 |
| 950 | GOBP_REGULATION_OF_ALPHA_BETA_T_CELL_ACTIVATION | 1.569 | 0.001 | 0.009 | 0.007 |
| 951 | GOBP_POSITIVE_REGULATION_OF_OSTEOBLAST_DIFFERENTIATION | 1.623 | 0.001 | 0.009 | 0.007 |
| 952 | GOBP_NEGATIVE_REGULATION_OF_G_PROTEIN_COUPLED_RECEPTOR_SIGNALING_PATHWAY | 1.623 | 0.001 | 0.009 | 0.007 |
| 953 | GOBP_REGULATION_OF_BODY_FLUID_LEVELS | 1.363 | 0.001 | 0.009 | 0.007 |
| 954 | GOBP_NEGATIVE_REGULATION_OF_LEUKOCYTE_APOPTOTIC_PROCESS | 1.631 | 0.001 | 0.009 | 0.007 |
| 955 | HP_DIFFICULTY_RUNNING | 1.633 | 0.001 | 0.009 | 0.007 |
| 956 | HP_ABNORMAL_PULMONARY_VALVE_PHYSIOLOGY | 1.503 | 0.001 | 0.009 | 0.007 |
| 957 | GOBP_CELLULAR_RESPONSE_TO_COPPER_ION | 1.672 | 0.001 | 0.009 | 0.007 |
| 958 | HP_EPISTAXIS | 1.559 | 0.001 | 0.009 | 0.007 |
| 959 | GOBP_ENDOCHONDRAL_BONE_MORPHOGENESIS | 1.630 | 0.001 | 0.009 | 0.007 |
| 960 | HP_PEDAL_EDEMA | 1.679 | 0.001 | 0.009 | 0.007 |
| 961 | HP_ABNORMAL_LEFT_VENTRICLE_MORPHOLOGY | 1.566 | 0.001 | 0.009 | 0.007 |
| 962 | GOBP_NEGATIVE_REGULATION_OF_MYELOID_LEUKOCYTE_DIFFERENTIATION | 1.634 | 0.001 | 0.009 | 0.007 |
| 963 | GOBP_POSITIVE_REGULATION_OF_NEURON_PROJECTION_DEVELOPMENT | 1.494 | 0.001 | 0.009 | 0.007 |
| 964 | GOCC_MICROFIBRIL | 1.658 | 0.001 | 0.009 | 0.007 |
| 965 | GOBP_SENSORY_PERCEPTION_OF_PAIN | 1.559 | 0.001 | 0.009 | 0.007 |
| 966 | GOBP_POSITIVE_REGULATION_OF_PROTEIN_POLYMERIZATION | 1.560 | 0.001 | 0.009 | 0.007 |
| 967 | GOBP_POSITIVE_REGULATION_OF_SMALL_GTPASE_MEDIATED_SIGNAL_TRANSDUCTION | 1.615 | 0.001 | 0.009 | 0.007 |
| 968 | GOBP_RESPONSE_TO_MECHANICAL_STIMULUS | 1.465 | 0.001 | 0.009 | 0.007 |
| 969 | HP_MUSCLE_HYPERTROPHY_OF_THE_LOWER_EXTREMITIES | 1.624 | 0.001 | 0.009 | 0.007 |
| 970 | GOBP_RECEPTOR_METABOLIC_PROCESS | 1.484 | 0.001 | 0.009 | 0.007 |
| 971 | GOBP_NEGATIVE_REGULATION_OF_PHAGOCYTOSIS | 1.673 | 0.001 | 0.009 | 0.007 |
| 972 | GOBP_AMINOGLYCAN_METABOLIC_PROCESS | 1.518 | 0.001 | 0.009 | 0.007 |
| 973 | GOCC_EXTRINSIC_COMPONENT_OF_MEMBRANE | 1.377 | 0.001 | 0.009 | 0.007 |
| 974 | HP_ABNORMALITY_OF_THE_TEMPOROMANDIBULAR_JOINT | 1.651 | 0.001 | 0.010 | 0.007 |
| 975 | HP_ABNORMAL_THUMB_MORPHOLOGY | 1.338 | 0.001 | 0.010 | 0.007 |
| 976 | HP_DIAPHRAGMATIC_WEAKNESS | 1.674 | 0.001 | 0.010 | 0.007 |
| 977 | GOBP_RESPONSE_TO_COPPER_ION | 1.629 | 0.001 | 0.010 | 0.007 |
| 978 | HP_ABNORMAL_PYRAMIDAL_SIGN | 1.355 | 0.001 | 0.010 | 0.007 |
| 979 | GOBP_AMINO_SUGAR_METABOLIC_PROCESS | 1.633 | 0.001 | 0.010 | 0.007 |
| 980 | HP_APLASIA_HYPOPLASIA_INVOLVING_THE_METACARPAL_BONES | 1.506 | 0.001 | 0.010 | 0.007 |
| 981 | GOBP_MYELOID_CELL_DIFFERENTIATION | 1.363 | 0.001 | 0.010 | 0.007 |
| 982 | GOBP_RHYTHMIC_PROCESS | 1.387 | 0.001 | 0.010 | 0.007 |
| 983 | GOCC_MHC_CLASS_II_PROTEIN_COMPLEX | 1.671 | 0.001 | 0.010 | 0.007 |
| 984 | HP_MYALGIA | 1.502 | 0.001 | 0.010 | 0.007 |
| 985 | GOBP_ANTIMICROBIAL_HUMORAL_RESPONSE | 1.541 | 0.001 | 0.010 | 0.007 |
| 986 | HP_CYANOSIS | 1.545 | 0.001 | 0.010 | 0.007 |
| 987 | GOBP_MYOTUBE_DIFFERENTIATION | 1.520 | 0.001 | 0.010 | 0.007 |
| 988 | HP_ABNORMALITY_OF_THE_PERIORBITAL_REGION | 1.416 | 0.001 | 0.010 | 0.007 |
| 989 | GOBP_DETOXIFICATION_OF_COPPER_ION | 1.670 | 0.001 | 0.010 | 0.007 |
| 990 | HP_PATENT_DUCTUS_ARTERIOSUS | 1.371 | 0.001 | 0.010 | 0.007 |
| 991 | GOBP_POSITIVE_REGULATION_OF_CANONICAL_WNT_SIGNALING_PATHWAY | 1.543 | 0.001 | 0.010 | 0.007 |
| 992 | GOBP_REGULATION_OF_EXCRETION | 1.678 | 0.001 | 0.010 | 0.007 |
| 993 | HP_PROTRUSIO_ACETABULI | 1.666 | 0.001 | 0.010 | 0.007 |
| 994 | GOBP_ENERGY_RESERVE_METABOLIC_PROCESS | 1.583 | 0.001 | 0.010 | 0.007 |
| 995 | HP_CHOROIDAL_NEOVASCULARIZATION | 1.628 | 0.001 | 0.010 | 0.008 |
| 996 | GOCC_LEADING_EDGE_MEMBRANE | 1.481 | 0.001 | 0.010 | 0.008 |
| 997 | HP_ABNORMAL_PHALANGEAL_JOINT_MORPHOLOGY_OF_THE_HAND | 1.450 | 0.001 | 0.010 | 0.008 |
| 998 | GOBP_SUBSTRATE_ADHESION_DEPENDENT_CELL_SPREADING | 1.550 | 0.001 | 0.010 | 0.008 |
| 999 | GOBP_REGULATION_OF_CELL_JUNCTION_ASSEMBLY | 1.458 | 0.001 | 0.010 | 0.008 |
| 1000 | GOBP_REGULATION_OF_LIPASE_ACTIVITY | 1.542 | 0.001 | 0.010 | 0.008 |
| 1001 | HP_SOFT_DOUGHY_SKIN | 1.643 | 0.001 | 0.010 | 0.008 |
| 1002 | GOBP_CELLULAR_RESPONSE_TO_PEPTIDE | 1.354 | 0.001 | 0.010 | 0.008 |
| 1003 | GOBP_MACROPHAGE_MIGRATION | 1.628 | 0.001 | 0.010 | 0.008 |
| 1004 | GOBP_CARDIAC_CHAMBER_DEVELOPMENT | 1.487 | 0.001 | 0.010 | 0.008 |
| 1005 | GOBP_POTASSIUM_ION_TRANSPORT | 1.420 | 0.001 | 0.010 | 0.008 |
| 1006 | HP_CONGENITAL_MALFORMATION_OF_THE_GREAT_ARTERIES | 1.316 | 0.001 | 0.010 | 0.008 |
| 1007 | GOBP_MEMBRANE_FUSION | 1.485 | 0.001 | 0.010 | 0.008 |
| 1008 | HP_ABNORMALITY_OF_SKELETAL_MUSCLE_FIBER_SIZE | 1.595 | 0.001 | 0.010 | 0.008 |
| 1009 | GOBP_ASTROCYTE_DIFFERENTIATION | 1.591 | 0.001 | 0.011 | 0.008 |
| 1010 | GOBP_POSITIVE_REGULATION_OF_B_CELL_ACTIVATION | 1.577 | 0.001 | 0.011 | 0.008 |
| 1011 | HP_ANKLE_SWELLING | 1.649 | 0.001 | 0.011 | 0.008 |
| 1012 | GOBP_POSITIVE_REGULATION_OF_MACROPHAGE_DERIVED_FOAM_CELL_DIFFERENTIATION | 1.625 | 0.001 | 0.011 | 0.008 |
| 1013 | GOBP_EPITHELIAL_CELL_APOPTOTIC_PROCESS | 1.524 | 0.001 | 0.011 | 0.008 |
| 1014 | HP_SKIN_ULCER | 1.513 | 0.001 | 0.011 | 0.008 |
| 1015 | GOBP_POSITIVE_REGULATION_OF_HUMORAL_IMMUNE_RESPONSE | 1.676 | 0.001 | 0.011 | 0.008 |
| 1016 | HP_OSTEOPOROSIS | 1.393 | 0.001 | 0.011 | 0.008 |
| 1017 | HP_INCREASED_BONE_MINERAL_DENSITY | 1.538 | 0.001 | 0.011 | 0.008 |
| 1018 | HP_ABNORMAL_ELECTROPHYSIOLOGY_OF_SINOATRIAL_NODE_ORIGIN | 1.591 | 0.001 | 0.011 | 0.008 |
| 1019 | GOBP_SMOOTH_MUSCLE_CELL_DIFFERENTIATION | 1.611 | 0.001 | 0.011 | 0.008 |
| 1020 | HP_ARTERIOVENOUS_FISTULA | 1.646 | 0.001 | 0.011 | 0.008 |
| 1021 | GOCC_CLATHRIN_COATED_ENDOCYTIC_VESICLE_MEMBRANE | 1.588 | 0.001 | 0.011 | 0.008 |
| 1022 | HP_SCARRING | 1.471 | 0.001 | 0.011 | 0.008 |
| 1023 | GOBP_POSITIVE_CHEMOTAXIS | 1.611 | 0.001 | 0.011 | 0.008 |
| 1024 | GOBP_NEGATIVE_REGULATION_OF_EXTRINSIC_APOPTOTIC_SIGNALING_PATHWAY_VIA_DEATH_DOMAIN_RECEPTORS | 1.650 | 0.001 | 0.011 | 0.008 |
| 1025 | GOBP_REGULATION_OF_RESPONSE_TO_WOUNDING | 1.495 | 0.001 | 0.011 | 0.008 |
| 1026 | GOBP_REGULATION_OF_ESTABLISHMENT_OF_PLANAR_POLARITY | 1.623 | 0.001 | 0.011 | 0.008 |
| 1027 | GOBP_POSITIVE_REGULATION_OF_DEVELOPMENTAL_GROWTH | 1.473 | 0.001 | 0.011 | 0.008 |
| 1028 | HP_ABDOMINAL_DISTENTION | 1.508 | 0.001 | 0.011 | 0.009 |
| 1029 | HP_HYPOREFLEXIA_OF_LOWER_LIMBS | 1.633 | 0.001 | 0.011 | 0.009 |
| 1030 | GOBP_REGULATION_OF_WNT_SIGNALING_PATHWAY_PLANAR_CELL_POLARITY_PATHWAY | 1.642 | 0.001 | 0.011 | 0.009 |
| 1031 | GOBP_COMPLEMENT_ACTIVATION_LECTIN_PATHWAY | 1.645 | 0.001 | 0.011 | 0.009 |
| 1032 | GOBP_PHAGOSOME_MATURATION | 1.657 | 0.001 | 0.011 | 0.009 |
| 1033 | GOBP_NEGATIVE_REGULATION_OF_MONONUCLEAR_CELL_MIGRATION | 1.659 | 0.001 | 0.012 | 0.009 |
| 1034 | HP_ABNORMALITY_OF_THE_HUMERORADIAL_JOINT | 1.625 | 0.001 | 0.012 | 0.009 |
| 1035 | GOBP_HEART_VALVE_MORPHOGENESIS | 1.605 | 0.001 | 0.012 | 0.009 |
| 1036 | HP_JOINT_SUBLUXATION | 1.658 | 0.001 | 0.012 | 0.009 |
| 1037 | GOBP_CORONARY_VASCULATURE_DEVELOPMENT | 1.624 | 0.001 | 0.012 | 0.009 |
| 1038 | GOBP_POSITIVE_REGULATION_OF_SMOOTH_MUSCLE_CELL_MIGRATION | 1.664 | 0.001 | 0.012 | 0.009 |
| 1039 | GOMF_PATTERN_RECOGNITION_RECEPTOR_ACTIVITY | 1.654 | 0.001 | 0.012 | 0.009 |
| 1040 | GOMF_CHEMOKINE_BINDING | 1.652 | 0.001 | 0.012 | 0.009 |
| 1041 | GOBP_REGULATION_OF_STRIATED_MUSCLE_CELL_DIFFERENTIATION | 1.536 | 0.001 | 0.012 | 0.009 |
| 1042 | HP_OMPHALOCELE | 1.565 | 0.001 | 0.012 | 0.009 |
| 1043 | GOBP_OSTEOBLAST_PROLIFERATION | 1.629 | 0.001 | 0.012 | 0.009 |
| 1044 | HP_HIGH_NARROW_PALATE | 1.488 | 0.001 | 0.012 | 0.009 |
| 1045 | HP_DECREASED_CIRCULATING_IGA_LEVEL | 1.621 | 0.001 | 0.012 | 0.009 |
| 1046 | HP_INFLAMMATORY_ABNORMALITY_OF_THE_EYE | 1.416 | 0.001 | 0.012 | 0.009 |
| 1047 | HP_ABNORMAL_T_CELL_MORPHOLOGY | 1.598 | 0.001 | 0.012 | 0.009 |
| 1048 | HP_HYDROURETER | 1.590 | 0.001 | 0.012 | 0.009 |
| 1049 | HP_AUTOIMMUNE_ANTIBODY_POSITIVITY | 1.582 | 0.001 | 0.012 | 0.009 |
| 1050 | GOBP_POSITIVE_REGULATION_OF_EPITHELIAL_CELL_APOPTOTIC_PROCESS | 1.649 | 0.001 | 0.012 | 0.009 |
| 1051 | GOBP_METANEPHRIC_NEPHRON_DEVELOPMENT | 1.616 | 0.001 | 0.012 | 0.009 |
| 1052 | GOCC_INTERCALATED_DISC | 1.611 | 0.001 | 0.012 | 0.009 |
| 1053 | GOBP_REGULATION_OF_EPITHELIAL_TO_MESENCHYMAL_TRANSITION | 1.568 | 0.001 | 0.012 | 0.009 |
| 1054 | HP_GASTROINTESTINAL_INFLAMMATION | 1.516 | 0.001 | 0.012 | 0.009 |
| 1055 | HP_EMPHYSEMA | 1.612 | 0.001 | 0.012 | 0.009 |
| 1056 | GOBP_NEPHRON_DEVELOPMENT | 1.473 | 0.001 | 0.012 | 0.009 |
| 1057 | GOBP_POSITIVE_REGULATION_OF_MUSCLE_CELL_DIFFERENTIATION | 1.600 | 0.001 | 0.013 | 0.010 |
| 1058 | GOBP_CARDIAC_VENTRICLE_DEVELOPMENT | 1.494 | 0.001 | 0.013 | 0.010 |
| 1059 | GOBP_DEVELOPMENTAL_PIGMENTATION | 1.609 | 0.001 | 0.013 | 0.010 |
| 1060 | GOMF_PROTEIN_LIPID_COMPLEX_BINDING | 1.643 | 0.001 | 0.013 | 0.010 |
| 1061 | GOBP_RHO_PROTEIN_SIGNAL_TRANSDUCTION | 1.502 | 0.001 | 0.013 | 0.010 |
| 1062 | GOBP_POSITIVE_REGULATION_OF_HEART_GROWTH | 1.614 | 0.001 | 0.013 | 0.010 |
| 1063 | GOBP_POSITIVE_REGULATION_OF_NEUTROPHIL_MIGRATION | 1.623 | 0.001 | 0.013 | 0.010 |
| 1064 | GOCC_SITE_OF_POLARIZED_GROWTH | 1.477 | 0.001 | 0.013 | 0.010 |
| 1065 | GOBP_POSITIVE_REGULATION_OF_EPITHELIAL_CELL_MIGRATION | 1.464 | 0.001 | 0.013 | 0.010 |
| 1066 | GOBP_EXOCYTOSIS | 1.350 | 0.001 | 0.013 | 0.010 |
| 1067 | GOBP_SIGNAL_RELEASE | 1.325 | 0.001 | 0.013 | 0.010 |
| 1068 | HP_ABNORMALITY_OF_METACARPOPHALANGEAL_JOINT | 1.653 | 0.001 | 0.013 | 0.010 |
| 1069 | GOBP_POSITIVE_REGULATION_OF_EXTRACELLULAR_MATRIX_ORGANIZATION | 1.651 | 0.001 | 0.013 | 0.010 |
| 1070 | GOBP_REGULATION_OF_ACUTE_INFLAMMATORY_RESPONSE_TO_ANTIGENIC_STIMULUS | 1.634 | 0.001 | 0.013 | 0.010 |
| 1071 | GOBP_NEGATIVE_REGULATION_OF_NEUROINFLAMMATORY_RESPONSE | 1.633 | 0.001 | 0.013 | 0.010 |
| 1072 | HP_ECCHYMOSIS | 1.604 | 0.001 | 0.013 | 0.010 |
| 1073 | GOBP_REGULATION_OF_NEURONAL_SYNAPTIC_PLASTICITY | 1.610 | 0.001 | 0.013 | 0.010 |
| 1074 | GOBP_ACTION_POTENTIAL | 1.487 | 0.001 | 0.013 | 0.010 |
| 1075 | GOBP_LEUKOCYTE_TETHERING_OR_ROLLING | 1.647 | 0.001 | 0.013 | 0.010 |
| 1076 | HP_PERICARDIAL_EFFUSION | 1.657 | 0.001 | 0.013 | 0.010 |
| 1077 | HP_RESTRICTIVE_VENTILATORY_DEFECT | 1.560 | 0.001 | 0.013 | 0.010 |
| 1078 | GOBP_REGULATION_OF_CELL_CYCLE_G1_S_PHASE_TRANSITION | 1.469 | 0.001 | 0.013 | 0.010 |
| 1079 | GOBP_CAMERA_TYPE_EYE_DEVELOPMENT | 1.360 | 0.001 | 0.013 | 0.010 |
| 1080 | HP_DEVIATION_OF_THE_HAND_OR_OF_FINGERS_OF_THE_HAND | 1.323 | 0.001 | 0.013 | 0.010 |
| 1081 | HP_ABNORMALITY_OF_DENTAL_ERUPTION | 1.400 | 0.001 | 0.014 | 0.010 |
| 1082 | HP_ACCELERATED_SKELETAL_MATURATION | 1.604 | 0.001 | 0.014 | 0.010 |
| 1083 | HP_SEPSIS | 1.549 | 0.001 | 0.014 | 0.010 |
| 1084 | HP_HEMATOLOGICAL_NEOPLASM | 1.392 | 0.001 | 0.014 | 0.010 |
| 1085 | HP_HYPEREXTENSIBILITY_OF_THE_FINGER_JOINTS | 1.610 | 0.001 | 0.014 | 0.010 |
| 1086 | HP_AVASCULAR_NECROSIS_OF_THE_CAPITAL_FEMORAL_EPIPHYSIS | 1.645 | 0.001 | 0.014 | 0.010 |
| 1087 | GOBP_REGULATION_OF_DEVELOPMENTAL_GROWTH | 1.351 | 0.001 | 0.014 | 0.010 |
| 1088 | GOMF_GUANYL_NUCLEOTIDE_BINDING | 1.347 | 0.001 | 0.014 | 0.010 |
| 1089 | GOBP_CYTOKINE_PRODUCTION_INVOLVED_IN_IMMUNE_RESPONSE | 1.533 | 0.001 | 0.014 | 0.010 |
| 1090 | HP_ABNORMALITY_OF_THE_PULMONARY_VASCULATURE | 1.416 | 0.001 | 0.014 | 0.011 |
| 1091 | HP_ABNORMAL_SHAPE_OF_THE_FRONTAL_REGION | 1.344 | 0.001 | 0.014 | 0.011 |
| 1092 | HP_INCOMPLETE_PENETRANCE | 1.464 | 0.001 | 0.014 | 0.011 |
| 1093 | HP_ABNORMALITY_OF_THE_SPINAL_CORD | 1.364 | 0.001 | 0.014 | 0.011 |
| 1094 | GOBP_MEMBRANE_INVAGINATION | 1.587 | 0.002 | 0.014 | 0.011 |
| 1095 | GOBP_REGULATION_OF_ACTIN_FILAMENT_BASED_MOVEMENT | 1.605 | 0.002 | 0.014 | 0.011 |
| 1096 | HP_ROUND_FACE | 1.519 | 0.002 | 0.014 | 0.011 |
| 1097 | HP_GINGIVAL_BLEEDING | 1.618 | 0.002 | 0.014 | 0.011 |
| 1098 | GOBP_EMBRYONIC_ORGAN_DEVELOPMENT | 1.308 | 0.002 | 0.014 | 0.011 |
| 1099 | HP_ABNORMALITY_OF_THE_CARPAL_BONES | 1.565 | 0.002 | 0.015 | 0.011 |
| 1100 | GOMF_PEPTIDE_HORMONE_BINDING | 1.612 | 0.002 | 0.015 | 0.011 |
| 1101 | HP_ABNORMALITY_OF_THE_THORACIC_CAVITY | 1.603 | 0.002 | 0.015 | 0.011 |
| 1102 | GOBP_NATURAL_KILLER_CELL_ACTIVATION | 1.517 | 0.002 | 0.015 | 0.011 |
| 1103 | GOBP_TELENCEPHALON_DEVELOPMENT | 1.392 | 0.002 | 0.015 | 0.011 |
| 1104 | GOBP_HOMEOSTASIS_OF_NUMBER_OF_CELLS | 1.380 | 0.002 | 0.015 | 0.011 |
| 1105 | HP_ABNORMALITY_OF_THE_MAXILLA | 1.476 | 0.002 | 0.015 | 0.011 |
| 1106 | HP_LYMPHOMA | 1.513 | 0.002 | 0.015 | 0.011 |
| 1107 | GOBP_NEUROMUSCULAR_PROCESS_CONTROLLING_BALANCE | 1.600 | 0.002 | 0.015 | 0.011 |
| 1108 | GOBP_REGULATION_OF_SYNAPSE_STRUCTURE_OR_ACTIVITY | 1.424 | 0.002 | 0.015 | 0.011 |
| 1109 | GOBP_POSITIVE_REGULATION_OF_LYMPHOCYTE_MIGRATION | 1.646 | 0.002 | 0.015 | 0.011 |
| 1110 | GOCC_GOLGI_LUMEN | 1.546 | 0.002 | 0.015 | 0.011 |
| 1111 | GOBP_RETINA_DEVELOPMENT_IN_CAMERA_TYPE_EYE | 1.473 | 0.002 | 0.015 | 0.011 |
| 1112 | HP_BABINSKI_SIGN | 1.398 | 0.002 | 0.015 | 0.011 |
| 1113 | GOBP_TOLL_LIKE_RECEPTOR_SIGNALING_PATHWAY | 1.490 | 0.002 | 0.015 | 0.011 |
| 1114 | GOCC_INTRINSIC_COMPONENT_OF_POSTSYNAPTIC_MEMBRANE | 1.490 | 0.002 | 0.015 | 0.011 |
| 1115 | GOBP_CELLULAR_RESPONSE_TO_AMYLOID_BETA | 1.609 | 0.002 | 0.015 | 0.011 |
| 1116 | HP_DELAYED_ERUPTION_OF_TEETH | 1.404 | 0.002 | 0.015 | 0.011 |
| 1117 | GOBP_REGULATION_OF_PHOSPHOLIPASE_ACTIVITY | 1.590 | 0.002 | 0.015 | 0.011 |
| 1118 | GOBP_STRESS_ACTIVATED_PROTEIN_KINASE_SIGNALING_CASCADE | 1.396 | 0.002 | 0.015 | 0.011 |
| 1119 | HP_INCOORDINATION | 1.419 | 0.002 | 0.015 | 0.011 |
| 1120 | HP_CARDIAC_SHUNT | 1.628 | 0.002 | 0.015 | 0.011 |
| 1121 | GOMF_CHEMOATTRACTANT_ACTIVITY | 1.626 | 0.002 | 0.015 | 0.011 |
| 1122 | HP_ABNORMAL_PLATELET_FUNCTION | 1.600 | 0.002 | 0.015 | 0.011 |
| 1123 | GOBP_POSITIVE_REGULATION_OF_ADAPTIVE_IMMUNE_RESPONSE | 1.504 | 0.002 | 0.015 | 0.011 |
| 1124 | HP_ARTERIOVENOUS_MALFORMATION | 1.590 | 0.002 | 0.015 | 0.012 |
| 1125 | GOBP_REGULATION_OF_PROTEIN_DEPHOSPHORYLATION | 1.520 | 0.002 | 0.015 | 0.012 |
| 1126 | HP_FRONTAL_LOBE_DEMENTIA | 1.603 | 0.002 | 0.015 | 0.012 |
| 1127 | GOBP_REGULATION_OF_MUSCLE_ADAPTATION | 1.539 | 0.002 | 0.015 | 0.012 |
| 1128 | HP_RESTRICTIVE_CARDIOMYOPATHY | 1.658 | 0.002 | 0.015 | 0.012 |
| 1129 | GOBP_EPITHELIAL_TUBE_MORPHOGENESIS | 1.354 | 0.002 | 0.016 | 0.012 |
| 1130 | GOBP_REGULATION_OF_APOPTOTIC_SIGNALING_PATHWAY | 1.329 | 0.002 | 0.016 | 0.012 |
| 1131 | GOBP_PEPTIDE_ANTIGEN_ASSEMBLY_WITH_MHC_PROTEIN_COMPLEX | 1.624 | 0.002 | 0.016 | 0.012 |
| 1132 | GOBP_DENDRITIC_CELL_MIGRATION | 1.642 | 0.002 | 0.016 | 0.012 |
| 1133 | HP_ABNORMALITY_OF_LIMB_EPIPHYSIS_MORPHOLOGY | 1.489 | 0.002 | 0.016 | 0.012 |
| 1134 | HP_HEMIPLEGIA | 1.609 | 0.002 | 0.016 | 0.012 |
| 1135 | GOBP_CD8_POSITIVE_ALPHA_BETA_T_CELL_DIFFERENTIATION | 1.625 | 0.002 | 0.016 | 0.012 |
| 1136 | HP_BROAD_FOREHEAD | 1.441 | 0.002 | 0.016 | 0.012 |
| 1137 | HP_VERTIGO | 1.506 | 0.002 | 0.016 | 0.012 |
| 1138 | HP_FIRST_DEGREE_ATRIOVENTRICULAR_BLOCK | 1.642 | 0.002 | 0.016 | 0.012 |
| 1139 | HP_FIBULAR_HYPOPLASIA | 1.622 | 0.002 | 0.016 | 0.012 |
| 1140 | GOBP_REGULATION_OF_B_CELL_RECEPTOR_SIGNALING_PATHWAY | 1.625 | 0.002 | 0.016 | 0.012 |
| 1141 | GOBP_NEGATIVE_REGULATION_OF_BONE_MINERALIZATION | 1.612 | 0.002 | 0.016 | 0.012 |
| 1142 | HP_GASTROPARESIS | 1.630 | 0.002 | 0.016 | 0.012 |
| 1143 | GOBP_REGULATION_OF_RECEPTOR_BINDING | 1.619 | 0.002 | 0.016 | 0.012 |
| 1144 | HP_BROAD_FINGER | 1.448 | 0.002 | 0.016 | 0.012 |
| 1145 | HP_AORTIC_VALVE_STENOSIS | 1.600 | 0.002 | 0.016 | 0.012 |
| 1146 | HP_ABNORMAL_MORPHOLOGY_OF_THE_CHOROIDAL_VASCULATURE | 1.619 | 0.002 | 0.016 | 0.012 |
| 1147 | HP_STRIAE_DISTENSAE | 1.638 | 0.002 | 0.016 | 0.012 |
| 1148 | GOBP_NEURON_PROJECTION_REGENERATION | 1.594 | 0.002 | 0.016 | 0.012 |
| 1149 | GOBP_POSITIVE_REGULATION_OF_PHOSPHOLIPASE_ACTIVITY | 1.608 | 0.002 | 0.016 | 0.012 |
| 1150 | GOBP_CARDIAC_CHAMBER_MORPHOGENESIS | 1.477 | 0.002 | 0.016 | 0.012 |
| 1151 | GOCC_CAVEOLA | 1.545 | 0.002 | 0.016 | 0.012 |
| 1152 | HP_PULMONARY_INFILTRATES | 1.598 | 0.002 | 0.017 | 0.012 |
| 1153 | GOBP_REGULATION_OF_CELL_SIZE | 1.424 | 0.002 | 0.017 | 0.012 |
| 1154 | GOBP_POSITIVE_REGULATION_OF_PROTEIN_DEPHOSPHORYLATION | 1.584 | 0.002 | 0.017 | 0.013 |
| 1155 | HP_POSTERIORLY_ROTATED_EARS | 1.307 | 0.002 | 0.017 | 0.013 |
| 1156 | HP_AVASCULAR_NECROSIS | 1.565 | 0.002 | 0.017 | 0.013 |
| 1157 | GOMF_CARBOXYPEPTIDASE_ACTIVITY | 1.583 | 0.002 | 0.017 | 0.013 |
| 1158 | GOBP_CYCLIC_NUCLEOTIDE_MEDIATED_SIGNALING | 1.548 | 0.002 | 0.017 | 0.013 |
| 1159 | GOBP_POSITIVE_REGULATION_OF_CALCIUM_ION_IMPORT | 1.609 | 0.002 | 0.017 | 0.013 |
| 1160 | GOBP_STEM_CELL_DIFFERENTIATION | 1.412 | 0.002 | 0.017 | 0.013 |
| 1161 | GOBP_CELL_FATE_COMMITMENT | 1.388 | 0.002 | 0.017 | 0.013 |
| 1162 | GOBP_GLYCOPROTEIN_METABOLIC_PROCESS | 1.336 | 0.002 | 0.017 | 0.013 |
| 1163 | GOBP_RESPONSE_TO_PEPTIDE_HORMONE | 1.333 | 0.002 | 0.017 | 0.013 |
| 1164 | HP_WEAKNESS_DUE_TO_UPPER_MOTOR_NEURON_DYSFUNCTION | 1.289 | 0.002 | 0.017 | 0.013 |
| 1165 | GOBP_REGULATION_OF_LYMPHOCYTE_MIGRATION | 1.591 | 0.002 | 0.017 | 0.013 |
| 1166 | GOBP_RENAL_SODIUM_EXCRETION | 1.635 | 0.002 | 0.017 | 0.013 |
| 1167 | GOBP_CHEMOKINE_PRODUCTION | 1.526 | 0.002 | 0.017 | 0.013 |
| 1168 | GOBP_NEUROTRANSMITTER_TRANSPORT | 1.405 | 0.002 | 0.017 | 0.013 |
| 1169 | GOMF_VINCULIN_BINDING | 1.626 | 0.002 | 0.017 | 0.013 |
| 1170 | GOMF_HORMONE_BINDING | 1.531 | 0.002 | 0.017 | 0.013 |
| 1171 | HP_ABNORMALITY_OF_THE_SUPRAORBITAL_RIDGES | 1.517 | 0.002 | 0.017 | 0.013 |
| 1172 | GOBP_INTEGRIN_ACTIVATION | 1.625 | 0.002 | 0.017 | 0.013 |
| 1173 | GOBP_REGULATION_OF_CELL_ADHESION_MEDIATED_BY_INTEGRIN | 1.589 | 0.002 | 0.017 | 0.013 |
| 1174 | GOBP_INTERLEUKIN_2_PRODUCTION | 1.591 | 0.002 | 0.017 | 0.013 |
| 1175 | HP_PERIPHERAL_NEUROPATHY | 1.312 | 0.002 | 0.017 | 0.013 |
| 1176 | HP_DECREASED_CIRCULATING_IGG_LEVEL | 1.590 | 0.002 | 0.017 | 0.013 |
| 1177 | HP_ABNORMAL_STOMACH_MORPHOLOGY | 1.370 | 0.002 | 0.017 | 0.013 |
| 1178 | HP_ABNORMALITY_OF_MALAR_BONES | 1.402 | 0.002 | 0.018 | 0.013 |
| 1179 | GOBP_PHYSIOLOGICAL_CARDIAC_MUSCLE_HYPERTROPHY | 1.627 | 0.002 | 0.018 | 0.013 |
| 1180 | GOCC_INTERSTITIAL_MATRIX | 1.625 | 0.002 | 0.018 | 0.013 |
| 1181 | GOBP_MEMBRANE_REPOLARIZATION | 1.587 | 0.002 | 0.018 | 0.013 |
| 1182 | GOBP_MAST_CELL_ACTIVATION_INVOLVED_IN_IMMUNE_RESPONSE | 1.603 | 0.002 | 0.018 | 0.013 |
| 1183 | GOBP_RESPIRATORY_BURST | 1.634 | 0.002 | 0.018 | 0.013 |
| 1184 | HP_ABNORMALITY_OF_THE_ACHILLES_TENDON | 1.578 | 0.002 | 0.018 | 0.013 |
| 1185 | GOBP_REGULATION_OF_SMALL_GTPASE_MEDIATED_SIGNAL_TRANSDUCTION | 1.357 | 0.002 | 0.018 | 0.013 |
| 1186 | GOBP_POSTSYNAPSE_ORGANIZATION | 1.457 | 0.002 | 0.018 | 0.013 |
| 1187 | GOCC_SARCOPLASM | 1.567 | 0.002 | 0.018 | 0.014 |
| 1188 | GOBP_PROTEIN_AUTOPHOSPHORYLATION | 1.418 | 0.002 | 0.018 | 0.014 |
| 1189 | GOBP_T_CELL_ACTIVATION_INVOLVED_IN_IMMUNE_RESPONSE | 1.505 | 0.002 | 0.018 | 0.014 |
| 1190 | GOBP_PRODUCTION_OF_MOLECULAR_MEDIATOR_INVOLVED_IN_INFLAMMATORY_RESPONSE | 1.527 | 0.002 | 0.018 | 0.014 |
| 1191 | HP_ABNORMALITY_OF_THE_UTERINE_CERVIX | 1.616 | 0.002 | 0.018 | 0.014 |
| 1192 | HP_PARESTHESIA | 1.495 | 0.002 | 0.018 | 0.014 |
| 1193 | GOBP_CHONDROITIN_SULFATE_PROTEOGLYCAN_METABOLIC_PROCESS | 1.613 | 0.002 | 0.018 | 0.014 |
| 1194 | GOBP_HOMEOSTASIS_OF_NUMBER_OF_CELLS_WITHIN_A_TISSUE | 1.609 | 0.002 | 0.018 | 0.014 |
| 1195 | GOBP_AMINO_ACID_IMPORT | 1.578 | 0.002 | 0.018 | 0.014 |
| 1196 | GOBP_MUSCLE_ADAPTATION | 1.496 | 0.002 | 0.018 | 0.014 |
| 1197 | GOCC_CELL_CELL_JUNCTION | 1.294 | 0.002 | 0.018 | 0.014 |
| 1198 | GOBP_PROTEIN_LOCALIZATION_TO_EXTRACELLULAR_REGION | 1.338 | 0.002 | 0.018 | 0.014 |
| 1199 | HP_MUSCLE_STIFFNESS | 1.562 | 0.002 | 0.018 | 0.014 |
| 1200 | HP_ABNORMALITY_ON_PULMONARY_FUNCTION_TESTING | 1.473 | 0.002 | 0.018 | 0.014 |
| 1201 | GOBP_REGULATION_OF_CELL_SUBSTRATE_JUNCTION_ORGANIZATION | 1.580 | 0.002 | 0.018 | 0.014 |
| 1202 | HP_PULMONARY_ARTERY_STENOSIS | 1.552 | 0.002 | 0.018 | 0.014 |
| 1203 | GOBP_RESPONSE_TO_ACID_CHEMICAL | 1.477 | 0.002 | 0.019 | 0.014 |
| 1204 | GOBP_FEMALE_SEX_DIFFERENTIATION | 1.464 | 0.002 | 0.019 | 0.014 |
| 1205 | GOMF_TRANSFORMING_GROWTH_FACTOR_BETA_BINDING | 1.640 | 0.002 | 0.019 | 0.014 |
| 1206 | GOBP_CELLULAR_RESPONSE_TO_ACID_CHEMICAL | 1.547 | 0.002 | 0.019 | 0.014 |
| 1207 | GOBP_POSITIVE_REGULATION_OF_CELL_ADHESION_MEDIATED_BY_INTEGRIN | 1.615 | 0.002 | 0.019 | 0.014 |
| 1208 | GOBP_NEURON_PROJECTION_EXTENSION | 1.457 | 0.002 | 0.019 | 0.014 |
| 1209 | HP_WIDE_ANTERIOR_FONTANEL | 1.550 | 0.002 | 0.019 | 0.014 |
| 1210 | GOBP_NEGATIVE_REGULATION_OF_SECRETION | 1.464 | 0.002 | 0.019 | 0.014 |
| 1211 | GOBP_AMINO_ACID_TRANSPORT | 1.472 | 0.002 | 0.019 | 0.014 |
| 1212 | HP_BROAD_RIBS | 1.613 | 0.002 | 0.019 | 0.014 |
| 1213 | GOBP_REGULATION_OF_NEUROTRANSMITTER_TRANSPORT | 1.509 | 0.002 | 0.019 | 0.014 |
| 1214 | HP_DERMAL_TRANSLUCENCY | 1.626 | 0.002 | 0.019 | 0.014 |
| 1215 | HP_HYPOREFLEXIA | 1.345 | 0.002 | 0.019 | 0.014 |
| 1216 | HP_SPLENOMEGALY | 1.333 | 0.002 | 0.019 | 0.014 |
| 1217 | GOBP_POSITIVE_REGULATION_OF_MONONUCLEAR_CELL_MIGRATION | 1.576 | 0.002 | 0.019 | 0.014 |
| 1218 | HP_SHALLOW_ORBITS | 1.618 | 0.002 | 0.019 | 0.015 |
| 1219 | GOBP_NEGATIVE_REGULATION_OF_ION_TRANSMEMBRANE_TRANSPORTER_ACTIVITY | 1.538 | 0.002 | 0.019 | 0.015 |
| 1220 | HP_HEMATOCHEZIA | 1.614 | 0.002 | 0.019 | 0.015 |
| 1221 | GOBP_CELLULAR_RESPONSE_TO_REACTIVE_OXYGEN_SPECIES | 1.476 | 0.002 | 0.019 | 0.015 |
| 1222 | GOBP_NEGATIVE_REGULATION_OF_VASCULAR_ASSOCIATED_SMOOTH_MUSCLE_CELL_MIGRATION | 1.611 | 0.002 | 0.019 | 0.015 |
| 1223 | HP_JOINT_SWELLING | 1.579 | 0.002 | 0.020 | 0.015 |
| 1224 | GOBP_SYNCYTIUM_FORMATION | 1.579 | 0.002 | 0.020 | 0.015 |
| 1225 | GOBP_T_HELPER_1_TYPE_IMMUNE_RESPONSE | 1.572 | 0.002 | 0.020 | 0.015 |
| 1226 | GOBP_REGULATION_OF_PHOSPHOPROTEIN_PHOSPHATASE_ACTIVITY | 1.569 | 0.002 | 0.020 | 0.015 |
| 1227 | GOBP_REGULATION_OF_POTASSIUM_ION_TRANSPORT | 1.504 | 0.002 | 0.020 | 0.015 |
| 1228 | GOMF_SCAVENGER_RECEPTOR_ACTIVITY | 1.593 | 0.002 | 0.020 | 0.015 |
| 1229 | GOBP_IMPORT_INTO_CELL | 1.392 | 0.002 | 0.020 | 0.015 |
| 1230 | GOBP_REGULATION_OF_MITOTIC_CELL_CYCLE_PHASE_TRANSITION | 1.343 | 0.002 | 0.020 | 0.015 |
| 1231 | HP_PROGRESSIVE_PROXIMAL_MUSCLE_WEAKNESS | 1.645 | 0.002 | 0.020 | 0.015 |
| 1232 | GOMF_VOLTAGE_GATED_CHANNEL_ACTIVITY | 1.426 | 0.002 | 0.020 | 0.015 |
| 1233 | HP_OCULAR_ANTERIOR_SEGMENT_DYSGENESIS | 1.350 | 0.002 | 0.020 | 0.015 |
| 1234 | GOBP_NEGATIVE_REGULATION_OF_PROTEIN_MATURATION | 1.605 | 0.002 | 0.020 | 0.015 |
| 1235 | GOBP_REGULATION_OF_PEPTIDYL_SERINE_PHOSPHORYLATION | 1.450 | 0.002 | 0.020 | 0.015 |
| 1236 | GOBP_HETEROTYPIC_CELL_CELL_ADHESION | 1.574 | 0.002 | 0.020 | 0.015 |
| 1237 | HP_NARROW_MOUTH | 1.373 | 0.002 | 0.020 | 0.015 |
| 1238 | GOBP_CHONDROITIN_SULFATE_BIOSYNTHETIC_PROCESS | 1.606 | 0.002 | 0.020 | 0.015 |
| 1239 | HP_OPPORTUNISTIC_INFECTION | 1.607 | 0.002 | 0.020 | 0.015 |
| 1240 | GOBP_RESPONSE_TO_HYDROGEN_PEROXIDE | 1.475 | 0.002 | 0.020 | 0.015 |
| 1241 | GOMF_GLYCOLIPID_BINDING | 1.594 | 0.002 | 0.020 | 0.015 |
| 1242 | GOCC_IMMUNOLOGICAL_SYNAPSE | 1.570 | 0.002 | 0.020 | 0.015 |
| 1243 | GOBP_ENDOCRINE_PROCESS | 1.535 | 0.002 | 0.020 | 0.015 |
| 1244 | GOBP_FOCAL_ADHESION_ASSEMBLY | 1.567 | 0.002 | 0.020 | 0.015 |
| 1245 | GOBP_POSITIVE_REGULATION_OF_ACUTE_INFLAMMATORY_RESPONSE | 1.611 | 0.002 | 0.020 | 0.015 |
| 1246 | GOBP_REGULATION_OF_OSTEOBLAST_PROLIFERATION | 1.613 | 0.002 | 0.020 | 0.015 |
| 1247 | GOBP_SMOOTHENED_SIGNALING_PATHWAY | 1.440 | 0.002 | 0.020 | 0.015 |
| 1248 | GOBP_MULTI_MULTICELLULAR_ORGANISM_PROCESS | 1.404 | 0.002 | 0.021 | 0.015 |
| 1249 | HP_OVOID_VERTEBRAL_BODIES | 1.592 | 0.003 | 0.021 | 0.016 |
| 1250 | HP_DEVIATION_OF_TOES | 1.430 | 0.003 | 0.021 | 0.016 |
| 1251 | GOBP_NEGATIVE_REGULATION_OF_CYSTEINE_TYPE_ENDOPEPTIDASE_ACTIVITY | 1.565 | 0.003 | 0.021 | 0.016 |
| 1252 | HP_ABNORMAL_RIGHT_ATRIUM_MORPHOLOGY | 1.607 | 0.003 | 0.021 | 0.016 |
| 1253 | GOBP_ACTIVATION_OF_PHOSPHOLIPASE_C_ACTIVITY | 1.611 | 0.003 | 0.021 | 0.016 |
| 1254 | GOBP_POSITIVE_REGULATION_OF_LIPASE_ACTIVITY | 1.561 | 0.003 | 0.021 | 0.016 |
| 1255 | GOMF_LOW_DENSITY_LIPOPROTEIN_PARTICLE_BINDING | 1.602 | 0.003 | 0.021 | 0.016 |
| 1256 | GOBP_REGULATION_OF_ENDOTHELIAL_CELL_MIGRATION | 1.402 | 0.003 | 0.021 | 0.016 |
| 1257 | HP_JUVENILE_ASEPTIC_NECROSIS | 1.621 | 0.003 | 0.021 | 0.016 |
| 1258 | GOMF_MICROTUBULE_BINDING | 1.364 | 0.003 | 0.021 | 0.016 |
| 1259 | GOBP_VACUOLAR_LOCALIZATION | 1.522 | 0.003 | 0.021 | 0.016 |
| 1260 | GOBP_PROTEIN_LOCALIZATION_TO_CELL_SURFACE | 1.555 | 0.003 | 0.021 | 0.016 |
| 1261 | GOBP_PROTEOGLYCAN_METABOLIC_PROCESS | 1.563 | 0.003 | 0.021 | 0.016 |
| 1262 | GOBP_POSITIVE_REGULATION_OF_PRODUCTION_OF_MOLECULAR_MEDIATOR_OF_IMMUNE_RESPONSE | 1.479 | 0.003 | 0.021 | 0.016 |
| 1263 | HP_NOCTURNAL_HYPOVENTILATION | 1.639 | 0.003 | 0.021 | 0.016 |
| 1264 | GOBP_CONVERGENT_EXTENSION | 1.615 | 0.003 | 0.022 | 0.016 |
| 1265 | HP_PLEURITIS | 1.630 | 0.003 | 0.022 | 0.016 |
| 1266 | GOBP_VASCULAR_ASSOCIATED_SMOOTH_MUSCLE_CELL_DIFFERENTIATION | 1.603 | 0.003 | 0.022 | 0.016 |
| 1267 | GOBP_NEGATIVE_REGULATION_OF_TISSUE_REMODELING | 1.617 | 0.003 | 0.022 | 0.016 |
| 1268 | GOBP_CD4_POSITIVE_ALPHA_BETA_T_CELL_DIFFERENTIATION | 1.537 | 0.003 | 0.022 | 0.016 |
| 1269 | HP_MILD_SHORT_STATURE | 1.600 | 0.003 | 0.022 | 0.016 |
| 1270 | GOBP_RESPONSE_TO_CADMIUM_ION | 1.562 | 0.003 | 0.022 | 0.016 |
| 1271 | HP_ABSENT_TOE | 1.499 | 0.003 | 0.022 | 0.017 |
| 1272 | HP_MYOPIA | 1.322 | 0.003 | 0.022 | 0.017 |
| 1273 | GOBP_POSITIVE_REGULATION_OF_NEUROINFLAMMATORY_RESPONSE | 1.604 | 0.003 | 0.022 | 0.017 |
| 1274 | GOBP_REGULATION_OF_RAS_PROTEIN_SIGNAL_TRANSDUCTION | 1.400 | 0.003 | 0.022 | 0.017 |
| 1275 | HP_OVERGROWTH | 1.625 | 0.003 | 0.022 | 0.017 |
| 1276 | HP_FREQUENT_FALLS | 1.525 | 0.003 | 0.022 | 0.017 |
| 1277 | GOBP_CARDIAC_VENTRICLE_MORPHOGENESIS | 1.548 | 0.003 | 0.022 | 0.017 |
| 1278 | HP_PREMATURE_BIRTH | 1.442 | 0.003 | 0.022 | 0.017 |
| 1279 | GOBP_SEMI_LUNAR_VALVE_DEVELOPMENT | 1.580 | 0.003 | 0.023 | 0.017 |
| 1280 | HP_ARTHROPATHY | 1.580 | 0.003 | 0.023 | 0.017 |
| 1281 | HP_DEEPLY_SET_EYE | 1.392 | 0.003 | 0.023 | 0.017 |
| 1282 | GOBP_REGULATION_OF_NITRIC_OXIDE_METABOLIC_PROCESS | 1.571 | 0.003 | 0.023 | 0.017 |
| 1283 | GOMF_MHC_CLASS_II_PROTEIN_COMPLEX_BINDING | 1.610 | 0.003 | 0.023 | 0.017 |
| 1284 | GOMF_METALLOCARBOXYPEPTIDASE_ACTIVITY | 1.598 | 0.003 | 0.023 | 0.017 |
| 1285 | GOBP_CD4_POSITIVE_ALPHA_BETA_T_CELL_ACTIVATION | 1.520 | 0.003 | 0.023 | 0.017 |
| 1286 | GOMF_LIPOPOLYSACCHARIDE_BINDING | 1.601 | 0.003 | 0.023 | 0.017 |
| 1287 | GOBP_MORPHOGENESIS_OF_A_BRANCHING_STRUCTURE | 1.417 | 0.003 | 0.023 | 0.017 |
| 1288 | GOMF_STRUCTURAL_CONSTITUENT_OF_EYE_LENS | 1.605 | 0.003 | 0.023 | 0.017 |
| 1289 | GOBP_REGULATION_OF_STRESS_ACTIVATED_PROTEIN_KINASE_SIGNALING_CASCADE | 1.423 | 0.003 | 0.023 | 0.017 |
| 1290 | HP_ASCENDING_TUBULAR_AORTA_ANEURYSM | 1.590 | 0.003 | 0.023 | 0.017 |
| 1291 | GOMF_MHC_PROTEIN_COMPLEX_BINDING | 1.620 | 0.003 | 0.023 | 0.017 |
| 1292 | GOCC_CLATHRIN_COATED_ENDOCYTIC_VESICLE | 1.522 | 0.003 | 0.023 | 0.017 |
| 1293 | GOBP_POSITIVE_REGULATION_OF_OSSIFICATION | 1.563 | 0.003 | 0.023 | 0.017 |
| 1294 | HP_GLOMERULOPATHY | 1.575 | 0.003 | 0.023 | 0.017 |
| 1295 | HP_ABNORMAL_ST_SEGMENT | 1.620 | 0.003 | 0.023 | 0.018 |
| 1296 | HP_PULMONARY_ARTERY_DILATATION | 1.580 | 0.003 | 0.023 | 0.018 |
| 1297 | GOCC_ANCHORED_COMPONENT_OF_PLASMA_MEMBRANE | 1.566 | 0.003 | 0.023 | 0.018 |
| 1298 | GOBP_MODULATION_BY_HOST_OF_SYMBIONT_PROCESS | 1.523 | 0.003 | 0.024 | 0.018 |
| 1299 | HP_ABNORMALITY_OF_PAIN_SENSATION | 1.486 | 0.003 | 0.024 | 0.018 |
| 1300 | GOCC_GLYCOPROTEIN_COMPLEX | 1.587 | 0.003 | 0.024 | 0.018 |
| 1301 | HP_SPONTANEOUS_HEMATOMAS | 1.587 | 0.003 | 0.024 | 0.018 |
| 1302 | GOBP_REACTIVE_NITROGEN_SPECIES_METABOLIC_PROCESS | 1.553 | 0.003 | 0.024 | 0.018 |
| 1303 | GOBP_CELL_CELL_RECOGNITION | 1.538 | 0.003 | 0.024 | 0.018 |
| 1304 | HP_ABNORMAL_CIRCULATING_IGA_LEVEL | 1.537 | 0.003 | 0.024 | 0.018 |
| 1305 | GOBP_REGULATION_OF_CARDIAC_MUSCLE_CONTRACTION_BY_REGULATION_OF_THE_RELEASE_OF_SEQUESTERED_CALCIUM_ION | 1.600 | 0.003 | 0.024 | 0.018 |
| 1306 | HP_ABNORMALITY_OF_THE_AUTONOMIC_NERVOUS_SYSTEM | 1.378 | 0.003 | 0.024 | 0.018 |
| 1307 | GOBP_REGULATION_OF_SMOOTH_MUSCLE_CELL_DIFFERENTIATION | 1.613 | 0.003 | 0.024 | 0.018 |
| 1308 | GOBP_POSITIVE_REGULATION_OF_SECRETION | 1.329 | 0.003 | 0.024 | 0.018 |
| 1309 | GOBP_POSITIVE_REGULATION_OF_MACROPHAGE_ACTIVATION | 1.604 | 0.003 | 0.024 | 0.018 |
| 1310 | HP_ABNORMAL_CHOROID_MORPHOLOGY | 1.395 | 0.003 | 0.024 | 0.018 |
| 1311 | GOMF_HYALURONIC_ACID_BINDING | 1.604 | 0.003 | 0.025 | 0.018 |
| 1312 | HP_ABNORMALITY_OF_THE_CALCANEUS | 1.593 | 0.003 | 0.025 | 0.019 |
| 1313 | HP_CALCIFICATION_OF_CARTILAGE | 1.612 | 0.003 | 0.025 | 0.019 |
| 1314 | GOBP_RESPONSE_TO_OXIDATIVE_STRESS | 1.300 | 0.003 | 0.025 | 0.019 |
| 1315 | GOBP_POSITIVE_REGULATION_OF_BINDING | 1.427 | 0.003 | 0.025 | 0.019 |
| 1316 | GOBP_REGULATION_OF_MEMBRANE_PROTEIN_ECTODOMAIN_PROTEOLYSIS | 1.620 | 0.003 | 0.025 | 0.019 |
| 1317 | GOBP_ELASTIC_FIBER_ASSEMBLY | 1.611 | 0.003 | 0.025 | 0.019 |
| 1318 | GOBP_POSITIVE_REGULATION_OF_FILOPODIUM_ASSEMBLY | 1.580 | 0.003 | 0.025 | 0.019 |
| 1319 | GOBP_RESPONSE_TO_STEROID_HORMONE | 1.337 | 0.003 | 0.025 | 0.019 |
| 1320 | GOBP_FIBROBLAST_MIGRATION | 1.552 | 0.003 | 0.025 | 0.019 |
| 1321 | GOBP_NEGATIVE_REGULATION_OF_B_CELL_ACTIVATION | 1.608 | 0.003 | 0.025 | 0.019 |
| 1322 | GOBP_NEGATIVE_REGULATION_OF_OSSIFICATION | 1.609 | 0.003 | 0.025 | 0.019 |
| 1323 | HP_TIBIAL_BOWING | 1.616 | 0.003 | 0.025 | 0.019 |
| 1324 | HP_DECREASED_LYMPHOCYTE_PROLIFERATION_IN_RESPONSE_TO_MITOGEN | 1.597 | 0.003 | 0.025 | 0.019 |
| 1325 | GOBP_REGULATION_OF_DNA_BINDING_TRANSCRIPTION_FACTOR_ACTIVITY | 1.282 | 0.003 | 0.025 | 0.019 |
| 1326 | GOBP_LEUKOCYTE_ADHESION_TO_VASCULAR_ENDOTHELIAL_CELL | 1.560 | 0.003 | 0.025 | 0.019 |
| 1327 | HP_ABNORMAL_MUSCLE_FIBER_TYPE_DISTRIBUTION | 1.560 | 0.003 | 0.025 | 0.019 |
| 1328 | GOBP_MEMBRANE_DEPOLARIZATION | 1.521 | 0.003 | 0.025 | 0.019 |
| 1329 | HP_LIMITATION_OF_KNEE_MOBILITY | 1.606 | 0.003 | 0.025 | 0.019 |
| 1330 | GOBP_NEUTROPHIL_EXTRAVASATION | 1.606 | 0.003 | 0.025 | 0.019 |
| 1331 | GOBP_POSITIVE_REGULATION_OF_RELEASE_OF_SEQUESTERED_CALCIUM_ION_INTO_CYTOSOL | 1.566 | 0.003 | 0.026 | 0.019 |
| 1332 | GOBP_NEGATIVE_REGULATION_OF_PROTEIN_BINDING | 1.490 | 0.003 | 0.026 | 0.019 |
| 1333 | HP_ABNORMAL_MORPHOLOGY_OF_THE_MUSCULATURE_OF_THE_NECK | 1.547 | 0.003 | 0.026 | 0.019 |
| 1334 | HP_LIMITED_ELBOW_EXTENSION | 1.575 | 0.003 | 0.026 | 0.019 |
| 1335 | HP_RECURRENT_ABSCESS_FORMATION | 1.605 | 0.003 | 0.026 | 0.019 |
| 1336 | GOBP_REGULATION_OF_WOUND_HEALING | 1.454 | 0.003 | 0.026 | 0.019 |
| 1337 | HP_RECURRENT_FRACTURES | 1.458 | 0.003 | 0.026 | 0.020 |
| 1338 | GOBP_GLIAL_CELL_DEVELOPMENT | 1.457 | 0.003 | 0.026 | 0.020 |
| 1339 | GOBP_CELLULAR_RESPONSE_TO_CHEMICAL_STRESS | 1.328 | 0.003 | 0.026 | 0.020 |
| 1340 | GOBP_POSTSYNAPTIC_SPECIALIZATION_ASSEMBLY | 1.581 | 0.003 | 0.026 | 0.020 |
| 1341 | HP_LIMB_PAIN | 1.572 | 0.003 | 0.026 | 0.020 |
| 1342 | HP_ASTIGMATISM | 1.397 | 0.003 | 0.026 | 0.020 |
| 1343 | GOBP_POSITIVE_REGULATION_OF_ENDOTHELIAL_CELL_MIGRATION | 1.442 | 0.003 | 0.026 | 0.020 |
| 1344 | GOBP_PEPTIDYL_SERINE_MODIFICATION | 1.322 | 0.003 | 0.026 | 0.020 |
| 1345 | GOBP_REGULATION_OF_BONE_REMODELING | 1.565 | 0.003 | 0.027 | 0.020 |
| 1346 | HP_POLYPHAGIA | 1.552 | 0.003 | 0.027 | 0.020 |
| 1347 | GOBP_LYMPHOCYTE_HOMEOSTASIS | 1.515 | 0.003 | 0.027 | 0.020 |
| 1348 | GOBP_HETEROPHILIC_CELL_CELL_ADHESION_VIA_PLASMA_MEMBRANE_CELL_ADHESION_MOLECULES | 1.575 | 0.003 | 0.027 | 0.020 |
| 1349 | HP_ABNORMALITY_OF_THE_SMALL_INTESTINE | 1.365 | 0.004 | 0.027 | 0.020 |
| 1350 | GOBP_CALCIUM_DEPENDENT_CELL_CELL_ADHESION_VIA_PLASMA_MEMBRANE_CELL_ADHESION_MOLECULES | 1.570 | 0.004 | 0.027 | 0.020 |
| 1351 | HP_GASTROESOPHAGEAL_REFLUX | 1.297 | 0.004 | 0.027 | 0.020 |
| 1352 | GOBP_RESPONSE_TO_NITRIC_OXIDE | 1.587 | 0.004 | 0.027 | 0.020 |
| 1353 | GOBP_ENTERIC_NERVOUS_SYSTEM_DEVELOPMENT | 1.595 | 0.004 | 0.027 | 0.020 |
| 1354 | GOBP_CARDIAC_MUSCLE_CELL_ACTION_POTENTIAL_INVOLVED_IN_CONTRACTION | 1.567 | 0.004 | 0.027 | 0.020 |
| 1355 | GOCC_PRESYNAPTIC_ACTIVE_ZONE | 1.501 | 0.004 | 0.027 | 0.020 |
| 1356 | HP_PATELLAR_DISLOCATION | 1.515 | 0.004 | 0.027 | 0.020 |
| 1357 | GOBP_CYCLIC_NUCLEOTIDE_BIOSYNTHETIC_PROCESS | 1.602 | 0.004 | 0.027 | 0.021 |
| 1358 | GOBP_CELL_CELL_ADHESION_MEDIATED_BY_INTEGRIN | 1.624 | 0.004 | 0.027 | 0.021 |
| 1359 | HP_PRIMITIVE_REFLEX | 1.585 | 0.004 | 0.027 | 0.021 |
| 1360 | GOBP_REGULATION_OF_COAGULATION | 1.527 | 0.004 | 0.027 | 0.021 |
| 1361 | GOBP_REGULATION_OF_NEUTROPHIL_ACTIVATION | 1.595 | 0.004 | 0.027 | 0.021 |
| 1362 | GOBP_RESPIRATORY_SYSTEM_DEVELOPMENT | 1.407 | 0.004 | 0.028 | 0.021 |
| 1363 | GOMF_TRANSMEMBRANE_TRANSPORTER_BINDING | 1.442 | 0.004 | 0.028 | 0.021 |
| 1364 | GOBP_DETECTION_OF_MOLECULE_OF_BACTERIAL_ORIGIN | 1.591 | 0.004 | 0.028 | 0.021 |
| 1365 | HP_LONG_PHILTRUM | 1.307 | 0.004 | 0.028 | 0.021 |
| 1366 | HP_ORAL_ULCER | 1.559 | 0.004 | 0.028 | 0.021 |
| 1367 | HP_ABNORMAL_SHARPEY_FIBER_MORPHOLOGY | 1.607 | 0.004 | 0.028 | 0.021 |
| 1368 | HP_CUTIS_LAXA | 1.459 | 0.004 | 0.028 | 0.021 |
| 1369 | HP_ABNORMAL_ERYTHROCYTE_SEDIMENTATION_RATE | 1.559 | 0.004 | 0.028 | 0.021 |
| 1370 | GOBP_DENDRITIC_CELL_APOPTOTIC_PROCESS | 1.605 | 0.004 | 0.028 | 0.021 |
| 1371 | GOBP_CHEMICAL_SYNAPTIC_TRANSMISSION_POSTSYNAPTIC | 1.502 | 0.004 | 0.028 | 0.021 |
| 1372 | GOBP_RECEPTOR_SIGNALING_PATHWAY_VIA_STAT | 1.401 | 0.004 | 0.028 | 0.021 |
| 1373 | HP_BROAD_METATARSAL | 1.590 | 0.004 | 0.028 | 0.021 |
| 1374 | GOBP_PLATELET_DERIVED_GROWTH_FACTOR_RECEPTOR_SIGNALING_PATHWAY | 1.544 | 0.004 | 0.028 | 0.021 |
| 1375 | HP_ABNORMAL_T_CELL_SUBSET_DISTRIBUTION | 1.553 | 0.004 | 0.028 | 0.021 |
| 1376 | HP_CHRONIC_PULMONARY_OBSTRUCTION | 1.587 | 0.004 | 0.028 | 0.021 |
| 1377 | GOBP_ANATOMICAL_STRUCTURE_HOMEOSTASIS | 1.328 | 0.004 | 0.028 | 0.021 |
| 1378 | HP_PROLONGED_BLEEDING_TIME | 1.564 | 0.004 | 0.028 | 0.021 |
| 1379 | HP_ANTINUCLEAR_ANTIBODY_POSITIVITY | 1.598 | 0.004 | 0.028 | 0.021 |
| 1380 | GOBP_NEGATIVE_REGULATION_OF_MACROPHAGE_ACTIVATION | 1.579 | 0.004 | 0.028 | 0.021 |
| 1381 | GOBP_NEGATIVE_REGULATION_OF_MUSCLE_CONTRACTION | 1.594 | 0.004 | 0.028 | 0.021 |
| 1382 | GOBP_POSITIVE_REGULATION_OF_MYELOID_LEUKOCYTE_DIFFERENTIATION | 1.543 | 0.004 | 0.029 | 0.021 |
| 1383 | GOBP_REGULATION_OF_LIPID_METABOLIC_PROCESS | 1.318 | 0.004 | 0.029 | 0.021 |
| 1384 | HP_ABNORMALITY_OF_TEMPERATURE_REGULATION | 1.271 | 0.004 | 0.029 | 0.021 |
| 1385 | HP_FINGER_CLINODACTYLY | 1.317 | 0.004 | 0.029 | 0.021 |
| 1386 | HP_LEFT_VENTRICULAR_HYPERTROPHY | 1.540 | 0.004 | 0.029 | 0.022 |
| 1387 | HP_HYPERLIPOPROTEINEMIA | 1.597 | 0.004 | 0.029 | 0.022 |
| 1388 | GOBP_REGULATION_OF_MUSCLE_HYPERTROPHY | 1.547 | 0.004 | 0.029 | 0.022 |
| 1389 | GOBP_INFLAMMATORY_CELL_APOPTOTIC_PROCESS | 1.596 | 0.004 | 0.029 | 0.022 |
| 1390 | HP_LONG_EYELASHES | 1.499 | 0.004 | 0.029 | 0.022 |
| 1391 | GOBP_CARDIAC_MUSCLE_CELL_ACTION_POTENTIAL | 1.509 | 0.004 | 0.029 | 0.022 |
| 1392 | GOBP_CELLULAR_RESPONSE_TO_ABIOTIC_STIMULUS | 1.325 | 0.004 | 0.029 | 0.022 |
| 1393 | GOBP_FAT_CELL_DIFFERENTIATION | 1.358 | 0.004 | 0.029 | 0.022 |
| 1394 | HP_INCREASED_HEAD_CIRCUMFERENCE | 1.271 | 0.004 | 0.029 | 0.022 |
| 1395 | HP_BRADYKINESIA | 1.492 | 0.004 | 0.029 | 0.022 |
| 1396 | GOBP_NEGATIVE_REGULATION_OF_CELL_GROWTH_INVOLVED_IN_CARDIAC_MUSCLE_CELL_DEVELOPMENT | 1.586 | 0.004 | 0.029 | 0.022 |
| 1397 | GOBP_CARDIAC_EPITHELIAL_TO_MESENCHYMAL_TRANSITION | 1.603 | 0.004 | 0.030 | 0.022 |
| 1398 | HP_HEPATOSPLENOMEGALY | 1.412 | 0.004 | 0.030 | 0.022 |
| 1399 | GOBP_MEMBRANE_PROTEIN_ECTODOMAIN_PROTEOLYSIS | 1.568 | 0.004 | 0.030 | 0.022 |
| 1400 | GOBP_REGULATION_OF_PLATELET_ACTIVATION | 1.568 | 0.004 | 0.030 | 0.022 |
| 1401 | HP_PERIVENTRICULAR_HETEROTOPIA | 1.582 | 0.004 | 0.030 | 0.022 |
| 1402 | GOBP_ADULT_HEART_DEVELOPMENT | 1.589 | 0.004 | 0.030 | 0.022 |
| 1403 | GOBP_MACROPHAGE_DIFFERENTIATION | 1.566 | 0.004 | 0.030 | 0.022 |
| 1404 | GOBP_STRESS_FIBER_ASSEMBLY | 1.473 | 0.004 | 0.030 | 0.023 |
| 1405 | GOCC_T_CELL_RECEPTOR_COMPLEX | 1.606 | 0.004 | 0.030 | 0.023 |
| 1406 | GOBP_POSITIVE_REGULATION_OF_INNATE_IMMUNE_RESPONSE | 1.413 | 0.004 | 0.030 | 0.023 |
| 1407 | GOBP_EXTRACELLULAR_REGULATION_OF_SIGNAL_TRANSDUCTION | 1.586 | 0.004 | 0.030 | 0.023 |
| 1408 | GOBP_G_PROTEIN_COUPLED_RECEPTOR_SIGNALING_PATHWAY_INVOLVED_IN_HEART_PROCESS | 1.577 | 0.004 | 0.030 | 0.023 |
| 1409 | GOBP_DETECTION_OF_EXTERNAL_BIOTIC_STIMULUS | 1.590 | 0.004 | 0.030 | 0.023 |
| 1410 | GOBP_POSITIVE_REGULATION_OF_IMMUNOGLOBULIN_PRODUCTION | 1.543 | 0.004 | 0.030 | 0.023 |
| 1411 | GOCC_NEURON_PROJECTION_MEMBRANE | 1.563 | 0.004 | 0.030 | 0.023 |
| 1412 | GOCC_ION_CHANNEL_COMPLEX | 1.348 | 0.004 | 0.030 | 0.023 |
| 1413 | HP_ABNORMAL_BLOOD_GAS_LEVEL | 1.602 | 0.004 | 0.030 | 0.023 |
| 1414 | HP_TYPE_1_MUSCLE_FIBER_ATROPHY | 1.575 | 0.004 | 0.030 | 0.023 |
| 1415 | GOBP_REGULATION_OF_FIBROBLAST_MIGRATION | 1.596 | 0.004 | 0.030 | 0.023 |
| 1416 | HP_ABNORMAL_HEART_SOUND | 1.601 | 0.004 | 0.030 | 0.023 |
| 1417 | GOBP_PROTEIN_HOMOOLIGOMERIZATION | 1.366 | 0.004 | 0.031 | 0.023 |
| 1418 | HP_ESODEVIATION | 1.423 | 0.004 | 0.031 | 0.023 |
| 1419 | GOBP_POSITIVE_REGULATION_OF_NEURON_DIFFERENTIATION | 1.505 | 0.004 | 0.031 | 0.023 |
| 1420 | GOBP_REGULATION_OF_B_CELL_PROLIFERATION | 1.553 | 0.004 | 0.031 | 0.023 |
| 1421 | GOBP_POSITIVE_REGULATION_OF_MAP_KINASE_ACTIVITY | 1.453 | 0.004 | 0.031 | 0.023 |
| 1422 | HP_RIGHT_VENTRICULAR_DILATATION | 1.583 | 0.004 | 0.031 | 0.024 |
| 1423 | GOBP_REGULATION_OF_CELL_GROWTH_INVOLVED_IN_CARDIAC_MUSCLE_CELL_DEVELOPMENT | 1.574 | 0.004 | 0.031 | 0.024 |
| 1424 | GOBP_REGULATION_OF_CARDIAC_MUSCLE_CONTRACTION | 1.486 | 0.004 | 0.031 | 0.024 |
| 1425 | HP_PREMATURELY_AGED_APPEARANCE | 1.412 | 0.004 | 0.031 | 0.024 |
| 1426 | HP_MUSCLE_FIBER_SPLITTING | 1.585 | 0.004 | 0.032 | 0.024 |
| 1427 | GOBP_REGULATION_OF_T_HELPER_1_TYPE_IMMUNE_RESPONSE | 1.564 | 0.004 | 0.032 | 0.024 |
| 1428 | GOBP_REGULATION_OF_ANTIGEN_RECEPTOR_MEDIATED_SIGNALING_PATHWAY | 1.539 | 0.004 | 0.032 | 0.024 |
| 1429 | HP_ABNORMAL_CONSUMPTION_BEHAVIOR | 1.441 | 0.004 | 0.032 | 0.024 |
| 1430 | GOBP_EPITHELIAL_TO_MESENCHYMAL_TRANSITION_INVOLVED_IN_ENDOCARDIAL_CUSHION_FORMATION | 1.566 | 0.004 | 0.032 | 0.024 |
| 1431 | GOBP_CELLULAR_RESPONSE_TO_EXTERNAL_STIMULUS | 1.334 | 0.004 | 0.032 | 0.024 |
| 1432 | HP_ABNORMALITY_OF_THE_PHALANGES_OF_THE_HALLUX | 1.579 | 0.004 | 0.032 | 0.024 |
| 1433 | GOBP_DIGESTIVE_SYSTEM_DEVELOPMENT | 1.429 | 0.004 | 0.032 | 0.024 |
| 1434 | HP_LYMPHADENITIS | 1.585 | 0.004 | 0.032 | 0.024 |
| 1435 | HP_THICKENED_SKIN | 1.290 | 0.004 | 0.032 | 0.024 |
| 1436 | HP_ABNORMAL_LEFT_ATRIUM_MORPHOLOGY | 1.602 | 0.004 | 0.032 | 0.024 |
| 1437 | HP_ANTENATAL_ONSET | 1.577 | 0.004 | 0.032 | 0.024 |
| 1438 | HP_POSTURAL_INSTABILITY | 1.521 | 0.004 | 0.032 | 0.024 |
| 1439 | GOBP_REGULATION_OF_SYSTEMIC_ARTERIAL_BLOOD_PRESSURE_BY_RENIN_ANGIOTENSIN | 1.598 | 0.004 | 0.032 | 0.024 |
| 1440 | GOBP_POSITIVE_REGULATION_OF_BLOOD_PRESSURE | 1.591 | 0.005 | 0.032 | 0.024 |
| 1441 | GOBP_ENDOCARDIAL_CUSHION_MORPHOGENESIS | 1.543 | 0.005 | 0.033 | 0.025 |
| 1442 | GOBP_REGULATION_OF_MACROPHAGE_MIGRATION | 1.543 | 0.005 | 0.033 | 0.025 |
| 1443 | HP_ABNORMAL_MORPHOLOGY_OF_THE_ABDOMINAL_MUSCULATURE | 1.533 | 0.005 | 0.033 | 0.025 |
| 1444 | HP_ABNORMAL_PATELLA_MORPHOLOGY | 1.499 | 0.005 | 0.033 | 0.025 |
| 1445 | HP_ABNORMAL_RECTUM_MORPHOLOGY | 1.443 | 0.005 | 0.033 | 0.025 |
| 1446 | GOBP_NEGATIVE_REGULATION_OF_SMOOTH_MUSCLE_CONTRACTION | 1.572 | 0.005 | 0.033 | 0.025 |
| 1447 | GOBP_REGULATION_OF_CALCIUM_ION_TRANSMEMBRANE_TRANSPORTER_ACTIVITY | 1.491 | 0.005 | 0.033 | 0.025 |
| 1448 | HP_ABNORMAL_ILIUM_MORPHOLOGY | 1.457 | 0.005 | 0.033 | 0.025 |
| 1449 | HP_HYPOPLASIA_OF_THE_MUSCULATURE | 1.574 | 0.005 | 0.033 | 0.025 |
| 1450 | GOBP_REGULATION_OF_VASCULAR_ASSOCIATED_SMOOTH_MUSCLE_CELL_DIFFERENTIATION | 1.583 | 0.005 | 0.033 | 0.025 |
| 1451 | HP_ATROPHY_DEGENERATION_INVOLVING_THE_CAUDATE_NUCLEUS | 1.581 | 0.005 | 0.033 | 0.025 |
| 1452 | HP_ABNORMAL_CAUDATE_NUCLEUS_MORPHOLOGY | 1.596 | 0.005 | 0.033 | 0.025 |
| 1453 | GOBP_SARCOPLASMIC_RETICULUM_CALCIUM_ION_TRANSPORT | 1.542 | 0.005 | 0.033 | 0.025 |
| 1454 | GOBP_CHRONIC_INFLAMMATORY_RESPONSE | 1.614 | 0.005 | 0.033 | 0.025 |
| 1455 | GOBP_KILLING_OF_CELLS_OF_ANOTHER_ORGANISM | 1.586 | 0.005 | 0.033 | 0.025 |
| 1456 | GOBP_CELLULAR_RESPONSE_TO_NUTRIENT | 1.554 | 0.005 | 0.033 | 0.025 |
| 1457 | HP_AUTOIMMUNE_THROMBOCYTOPENIA | 1.553 | 0.005 | 0.033 | 0.025 |
| 1458 | HP_MYOTONIA | 1.597 | 0.005 | 0.033 | 0.025 |
| 1459 | HP_ANORECTAL_ANOMALY | 1.326 | 0.005 | 0.033 | 0.025 |
| 1460 | GOBP_POSITIVE_REGULATION_OF_EXTRINSIC_APOPTOTIC_SIGNALING_PATHWAY | 1.541 | 0.005 | 0.033 | 0.025 |
| 1461 | HP_DOLICHOCEPHALY | 1.391 | 0.005 | 0.033 | 0.025 |
| 1462 | GOBP_SUPEROXIDE_METABOLIC_PROCESS | 1.520 | 0.005 | 0.033 | 0.025 |
| 1463 | GOBP_CGMP_MEDIATED_SIGNALING | 1.589 | 0.005 | 0.033 | 0.025 |
| 1464 | GOBP_NEGATIVE_REGULATION_OF_PROTEIN_SERINE_THREONINE_KINASE_ACTIVITY | 1.453 | 0.005 | 0.033 | 0.025 |
| 1465 | HP_ABNORMAL_EATING_BEHAVIOR | 1.470 | 0.005 | 0.033 | 0.025 |
| 1466 | HP_INFLAMMATION_OF_THE_LARGE_INTESTINE | 1.504 | 0.005 | 0.033 | 0.025 |
| 1467 | HP_BELL_SHAPED_THORAX | 1.573 | 0.005 | 0.033 | 0.025 |
| 1468 | GOBP_REGULATION_OF_DENDRITIC_CELL_ANTIGEN_PROCESSING_AND_PRESENTATION | 1.596 | 0.005 | 0.033 | 0.025 |
| 1469 | GOBP_REGULATION_OF_CELL_MATRIX_ADHESION | 1.433 | 0.005 | 0.033 | 0.025 |
| 1470 | GOBP_RENAL_SYSTEM_PROCESS | 1.455 | 0.005 | 0.033 | 0.025 |
| 1471 | GOBP_POSITIVE_REGULATION_OF_NEURON_DEATH | 1.481 | 0.005 | 0.034 | 0.025 |
| 1472 | GOBP_CELLULAR_RESPONSE_TO_CAMP | 1.542 | 0.005 | 0.034 | 0.025 |
| 1473 | HP_SLENDER_BUILD | 1.563 | 0.005 | 0.034 | 0.025 |
| 1474 | GOBP_REGULATION_OF_APOPTOTIC_CELL_CLEARANCE | 1.583 | 0.005 | 0.034 | 0.025 |
| 1475 | GOBP_RECEPTOR_CLUSTERING | 1.553 | 0.005 | 0.034 | 0.025 |
| 1476 | GOBP_NEGATIVE_REGULATION_OF_TRANSPORTER_ACTIVITY | 1.478 | 0.005 | 0.034 | 0.025 |
| 1477 | GOBP_EPITHELIAL_CELL_DIFFERENTIATION_INVOLVED_IN_KIDNEY_DEVELOPMENT | 1.541 | 0.005 | 0.034 | 0.025 |
| 1478 | GOBP_RESPIRATORY_BURST_INVOLVED_IN_DEFENSE_RESPONSE | 1.582 | 0.005 | 0.034 | 0.026 |
| 1479 | GOBP_REGULATION_OF_CALCIUM_MEDIATED_SIGNALING | 1.499 | 0.005 | 0.034 | 0.026 |
| 1480 | HP_GASTROINTESTINAL_HEMORRHAGE | 1.425 | 0.005 | 0.034 | 0.026 |
| 1481 | GOBP_CELL_MIGRATION_INVOLVED_IN_HEART_DEVELOPMENT | 1.571 | 0.005 | 0.034 | 0.026 |
| 1482 | GOBP_AMINO_ACID_IMPORT_ACROSS_PLASMA_MEMBRANE | 1.543 | 0.005 | 0.034 | 0.026 |
| 1483 | GOCC_PRESYNAPTIC_ACTIVE_ZONE_MEMBRANE | 1.560 | 0.005 | 0.034 | 0.026 |
| 1484 | HP_ERUPTION_FAILURE | 1.611 | 0.005 | 0.034 | 0.026 |
| 1485 | HP_PROMINENT_SUPRAORBITAL_RIDGES | 1.551 | 0.005 | 0.035 | 0.026 |
| 1486 | GOBP_POSITIVE_REGULATION_OF_CELL_SUBSTRATE_JUNCTION_ORGANIZATION | 1.581 | 0.005 | 0.035 | 0.026 |
| 1487 | HP_ABNORMAL_ANTERIOR_CHAMBER_MORPHOLOGY | 1.532 | 0.005 | 0.035 | 0.026 |
| 1488 | HP_SHORT_PALM | 1.481 | 0.005 | 0.035 | 0.026 |
| 1489 | GOBP_BODY_MORPHOGENESIS | 1.542 | 0.005 | 0.035 | 0.026 |
| 1490 | HP_ABNORMALITY_OF_MOUTH_SIZE | 1.292 | 0.005 | 0.035 | 0.026 |
| 1491 | HP_RECURRENT_BRONCHITIS | 1.577 | 0.005 | 0.035 | 0.027 |
| 1492 | HP_CEREBRAL_CALCIFICATION | 1.438 | 0.005 | 0.035 | 0.027 |
| 1493 | HP_VENTRICULAR_TACHYCARDIA | 1.534 | 0.005 | 0.035 | 0.027 |
| 1494 | GOMF_PHOSPHORUS_OXYGEN_LYASE_ACTIVITY | 1.570 | 0.005 | 0.035 | 0.027 |
| 1495 | HP_EXERCISE_INTOLERANCE | 1.474 | 0.005 | 0.035 | 0.027 |
| 1496 | GOBP_NEGATIVE_REGULATION_OF_INTERFERON_GAMMA_PRODUCTION | 1.549 | 0.005 | 0.036 | 0.027 |
| 1497 | GOBP_SUBSTRATE_DEPENDENT_CELL_MIGRATION | 1.574 | 0.005 | 0.036 | 0.027 |
| 1498 | HP_HEADACHE | 1.343 | 0.005 | 0.036 | 0.027 |
| 1499 | GOBP_CELL_CYCLE_G1_S_PHASE_TRANSITION | 1.363 | 0.005 | 0.036 | 0.027 |
| 1500 | HP_ABNORMALITY_OF_SKIN_PHYSIOLOGY | 1.254 | 0.005 | 0.036 | 0.027 |
| 1501 | GOBP_NITRIC_OXIDE_SYNTHASE_BIOSYNTHETIC_PROCESS | 1.568 | 0.005 | 0.036 | 0.027 |
| 1502 | GOBP_POSITIVE_REGULATION_OF_MYELOID_LEUKOCYTE_MEDIATED_IMMUNITY | 1.556 | 0.005 | 0.036 | 0.027 |
| 1503 | HP_APLASIA_HYPOPLASIA_OF_THE_FIBULA | 1.570 | 0.005 | 0.036 | 0.027 |
| 1504 | HP_ABNORMAL_PLATELET_VOLUME | 1.565 | 0.005 | 0.036 | 0.027 |
| 1505 | GOBP_BLOOD_VESSEL_ENDOTHELIAL_CELL_MIGRATION | 1.395 | 0.005 | 0.036 | 0.027 |
| 1506 | HP_BROAD_METACARPALS | 1.608 | 0.005 | 0.036 | 0.027 |
| 1507 | HP_AMBLYOPIA | 1.495 | 0.005 | 0.036 | 0.027 |
| 1508 | GOBP_REGULATION_OF_CD8_POSITIVE_ALPHA_BETA_T_CELL_ACTIVATION | 1.534 | 0.005 | 0.036 | 0.027 |
| 1509 | GOBP_NEURON_APOPTOTIC_PROCESS | 1.352 | 0.005 | 0.036 | 0.027 |
| 1510 | GOMF_METALLOEXOPEPTIDASE_ACTIVITY | 1.518 | 0.005 | 0.036 | 0.027 |
| 1511 | HP_ECHOLALIA | 1.568 | 0.005 | 0.036 | 0.027 |
| 1512 | GOBP_PURINERGIC_NUCLEOTIDE_RECEPTOR_SIGNALING_PATHWAY | 1.557 | 0.005 | 0.036 | 0.027 |
| 1513 | GOBP_REGULATION_OF_MYELOID_CELL_APOPTOTIC_PROCESS | 1.587 | 0.005 | 0.036 | 0.027 |
| 1514 | GOBP_RELEASE_OF_SEQUESTERED_CALCIUM_ION_INTO_CYTOSOL_BY_ENDOPLASMIC_RETICULUM | 1.581 | 0.005 | 0.036 | 0.027 |
| 1515 | GOBP_NEUROMUSCULAR_PROCESS | 1.420 | 0.005 | 0.037 | 0.027 |
| 1516 | GOBP_EXTRINSIC_APOPTOTIC_SIGNALING_PATHWAY_VIA_DEATH_DOMAIN_RECEPTORS | 1.496 | 0.005 | 0.037 | 0.028 |
| 1517 | HP_MUSCLE_FIBER_ATROPHY | 1.511 | 0.005 | 0.037 | 0.028 |
| 1518 | GOCC_CYTOPLASMIC_SIDE_OF_MEMBRANE | 1.376 | 0.005 | 0.037 | 0.028 |
| 1519 | HP_CRANIOSYNOSTOSIS | 1.423 | 0.005 | 0.037 | 0.028 |
| 1520 | GOBP_FIBROBLAST_GROWTH_FACTOR_RECEPTOR_SIGNALING_PATHWAY | 1.478 | 0.005 | 0.037 | 0.028 |
| 1521 | GOBP_VASCULAR_ENDOTHELIAL_GROWTH_FACTOR_RECEPTOR_SIGNALING_PATHWAY | 1.541 | 0.005 | 0.037 | 0.028 |
| 1522 | HP_RECURRENT_SINUSITIS | 1.546 | 0.005 | 0.037 | 0.028 |
| 1523 | GOBP_SEQUESTERING_OF_EXTRACELLULAR_LIGAND_FROM_RECEPTOR | 1.578 | 0.005 | 0.037 | 0.028 |
| 1524 | GOBP_CENTRAL_NERVOUS_SYSTEM_NEURON_DIFFERENTIATION | 1.393 | 0.005 | 0.037 | 0.028 |
| 1525 | GOCC_SECONDARY_LYSOSOME | 1.607 | 0.005 | 0.037 | 0.028 |
| 1526 | GOCC_TRANSPORT_VESICLE | 1.285 | 0.005 | 0.037 | 0.028 |
| 1527 | GOMF_MUSCLE_ALPHA_ACTININ_BINDING | 1.575 | 0.006 | 0.037 | 0.028 |
| 1528 | GOBP_MAINTENANCE_OF_PROTEIN_LOCATION | 1.453 | 0.006 | 0.037 | 0.028 |
| 1529 | GOBP_ROOF_OF_MOUTH_DEVELOPMENT | 1.452 | 0.006 | 0.037 | 0.028 |
| 1530 | HP_CAUDATE_ATROPHY | 1.567 | 0.006 | 0.037 | 0.028 |
| 1531 | HP_COR_PULMONALE | 1.574 | 0.006 | 0.037 | 0.028 |
| 1532 | GOBP_POSITIVE_REGULATION_OF_NIK_NF_KAPPAB_SIGNALING | 1.508 | 0.006 | 0.037 | 0.028 |
| 1533 | HP_BROAD_PHALANX_OF_THE_TOES | 1.533 | 0.006 | 0.037 | 0.028 |
| 1534 | GOBP_POSITIVE_REGULATION_OF_NITRIC_OXIDE_SYNTHASE_BIOSYNTHETIC_PROCESS | 1.605 | 0.006 | 0.038 | 0.028 |
| 1535 | GOCC_EXTRINSIC_COMPONENT_OF_CYTOPLASMIC_SIDE_OF_PLASMA_MEMBRANE | 1.456 | 0.006 | 0.038 | 0.028 |
| 1536 | HP_APLASIA_HYPOPLASIA_OF_FINGERS | 1.324 | 0.006 | 0.038 | 0.028 |
| 1537 | GOBP_REGULATION_OF_NEURON_DIFFERENTIATION | 1.373 | 0.006 | 0.038 | 0.029 |
| 1538 | GOBP_RELAXATION_OF_MUSCLE | 1.577 | 0.006 | 0.038 | 0.029 |
| 1539 | HP_ABNORMALITY_OF_SUBCUTANEOUS_FAT_TISSUE | 1.530 | 0.006 | 0.038 | 0.029 |
| 1540 | GOBP_IMMUNOGLOBULIN_PRODUCTION_INVOLVED_IN_IMMUNOGLOBULIN_MEDIATED_IMMUNE_RESPONSE | 1.515 | 0.006 | 0.038 | 0.029 |
| 1541 | GOMF_GROWTH_FACTOR_RECEPTOR_BINDING | 1.418 | 0.006 | 0.038 | 0.029 |
| 1542 | GOBP_REGULATION_OF_INNATE_IMMUNE_RESPONSE | 1.366 | 0.006 | 0.038 | 0.029 |
| 1543 | HP_TELECANTHUS | 1.427 | 0.006 | 0.038 | 0.029 |
| 1544 | GOBP_POSITIVE_REGULATION_OF_SUBSTRATE_ADHESION_DEPENDENT_CELL_SPREADING | 1.567 | 0.006 | 0.038 | 0.029 |
| 1545 | GOBP_NEGATIVE_REGULATION_OF_MYELOID_LEUKOCYTE_MEDIATED_IMMUNITY | 1.564 | 0.006 | 0.038 | 0.029 |
| 1546 | HP_ST_SEGMENT_DEPRESSION | 1.564 | 0.006 | 0.038 | 0.029 |
| 1547 | GOBP_REGULATION_OF_RECEPTOR_MEDIATED_ENDOCYTOSIS | 1.440 | 0.006 | 0.038 | 0.029 |
| 1548 | HP_FLAT_FACE | 1.452 | 0.006 | 0.038 | 0.029 |
| 1549 | GOBP_VENTRICULAR_CARDIAC_MUSCLE_TISSUE_MORPHOGENESIS | 1.554 | 0.006 | 0.038 | 0.029 |
| 1550 | GOBP_ENDOCARDIAL_CUSHION_DEVELOPMENT | 1.529 | 0.006 | 0.038 | 0.029 |
| 1551 | GOBP_NATURAL_KILLER_CELL_CHEMOTAXIS | 1.576 | 0.006 | 0.039 | 0.029 |
| 1552 | GOBP_NEGATIVE_REGULATION_OF_MUSCLE_CELL_DIFFERENTIATION | 1.503 | 0.006 | 0.039 | 0.029 |
| 1553 | GOBP_NEUROTRANSMITTER_SECRETION | 1.418 | 0.006 | 0.039 | 0.029 |
| 1554 | HP_NAUSEA | 1.490 | 0.006 | 0.039 | 0.029 |
| 1555 | GOBP_ANIMAL_ORGAN_REGENERATION | 1.492 | 0.006 | 0.039 | 0.029 |
| 1556 | GOBP_DETECTION_OF_ABIOTIC_STIMULUS | 1.393 | 0.006 | 0.039 | 0.029 |
| 1557 | HP_ABNORMALITY_OF_THE_MENSTRUAL_CYCLE | 1.353 | 0.006 | 0.039 | 0.029 |
| 1558 | HP_ABNORMALITY_OF_THE_ZYGOMATIC_BONE | 1.349 | 0.006 | 0.039 | 0.029 |
| 1559 | GOBP_T_CELL_CHEMOTAXIS | 1.573 | 0.006 | 0.039 | 0.029 |
| 1560 | HP_RESTLESS_LEGS | 1.573 | 0.006 | 0.039 | 0.029 |
| 1561 | HP_HALLUX_VALGUS | 1.489 | 0.006 | 0.039 | 0.029 |
| 1562 | GOBP_REGULATION_OF_TISSUE_REMODELING | 1.491 | 0.006 | 0.039 | 0.029 |
| 1563 | HP_NECK_MUSCLE_WEAKNESS | 1.517 | 0.006 | 0.039 | 0.030 |
| 1564 | GOBP_VASCULAR_TRANSPORT | 1.465 | 0.006 | 0.039 | 0.030 |
| 1565 | GOBP_REGULATION_OF_EXTRACELLULAR_MATRIX_DISASSEMBLY | 1.560 | 0.006 | 0.039 | 0.030 |
| 1566 | HP_ABNORMALITY_OF_NEUTROPHIL_PHYSIOLOGY | 1.557 | 0.006 | 0.039 | 0.030 |
| 1567 | GOCC_RUFFLE | 1.385 | 0.006 | 0.039 | 0.030 |
| 1568 | GOBP_MACROPHAGE_ACTIVATION_INVOLVED_IN_IMMUNE_RESPONSE | 1.601 | 0.006 | 0.039 | 0.030 |
| 1569 | GOBP_POSITIVE_REGULATION_OF_CARDIOCYTE_DIFFERENTIATION | 1.529 | 0.006 | 0.039 | 0.030 |
| 1570 | GOBP_REGULATION_OF_PLASMINOGEN_ACTIVATION | 1.570 | 0.006 | 0.039 | 0.030 |
| 1571 | GOBP_NEGATIVE_REGULATION_OF_DNA_BINDING_TRANSCRIPTION_FACTOR_ACTIVITY | 1.371 | 0.006 | 0.039 | 0.030 |
| 1572 | GOBP_CARDIAC_ATRIUM_DEVELOPMENT | 1.582 | 0.006 | 0.040 | 0.030 |
| 1573 | GOBP_REGULATION_OF_NERVOUS_SYSTEM_PROCESS | 1.441 | 0.006 | 0.040 | 0.030 |
| 1574 | HP_ADDUCTED_THUMB | 1.461 | 0.006 | 0.040 | 0.030 |
| 1575 | HP_LONG_HALLUX | 1.568 | 0.006 | 0.040 | 0.030 |
| 1576 | GOBP_REGULATION_OF_POSTSYNAPTIC_MEMBRANE_POTENTIAL | 1.413 | 0.006 | 0.040 | 0.030 |
| 1577 | HP_INCREASED_CONNECTIVE_TISSUE | 1.561 | 0.006 | 0.040 | 0.030 |
| 1578 | HP_INCREASED_LDL_CHOLESTEROL_CONCENTRATION | 1.559 | 0.006 | 0.040 | 0.030 |
| 1579 | HP_EPIPHYSEAL_DYSPLASIA | 1.557 | 0.006 | 0.040 | 0.030 |
| 1580 | GOBP_B_CELL_HOMEOSTASIS | 1.567 | 0.006 | 0.040 | 0.030 |
| 1581 | GOBP_LYMPHOCYTE_COSTIMULATION | 1.550 | 0.006 | 0.040 | 0.030 |
| 1582 | HP_DIFFICULTY_CLIMBING_STAIRS | 1.549 | 0.006 | 0.040 | 0.030 |
| 1583 | GOBP_POSITIVE_REGULATION_OF_ALPHA_BETA_T_CELL_DIFFERENTIATION | 1.524 | 0.006 | 0.040 | 0.030 |
| 1584 | HP_MEGALOCORNEA | 1.531 | 0.006 | 0.040 | 0.030 |
| 1585 | HP_APLASIA_HYPOPLASIA_OF_THE_LUNGS | 1.396 | 0.006 | 0.040 | 0.030 |
| 1586 | GOBP_RESPONSE_TO_METAL_ION | 1.308 | 0.006 | 0.040 | 0.030 |
| 1587 | GOCC_CATION_CHANNEL_COMPLEX | 1.355 | 0.006 | 0.040 | 0.030 |
| 1588 | HP_SYNOSTOSIS_OF_JOINTS | 1.369 | 0.006 | 0.040 | 0.030 |
| 1589 | GOBP_LEUKOCYTE_MEDIATED_CYTOTOXICITY | 1.411 | 0.006 | 0.040 | 0.030 |
| 1590 | HP_DEVIATION_OF_THE_HALLUX | 1.406 | 0.006 | 0.040 | 0.030 |
| 1591 | GOBP_RELAXATION_OF_SMOOTH_MUSCLE | 1.580 | 0.006 | 0.040 | 0.030 |
| 1592 | GOBP_NEGATIVE_REGULATION_OF_MITOTIC_CELL_CYCLE_PHASE_TRANSITION | 1.405 | 0.006 | 0.040 | 0.030 |
| 1593 | GOBP_REGULATION_OF_LEUKOCYTE_MEDIATED_CYTOTOXICITY | 1.487 | 0.006 | 0.040 | 0.030 |
| 1594 | GOBP_LUTEINIZATION | 1.557 | 0.006 | 0.041 | 0.031 |
| 1595 | HP_ABNORMAL_PLATELET_AGGREGATION | 1.556 | 0.006 | 0.041 | 0.031 |
| 1596 | HP_SEPTO_OPTIC_DYSPLASIA | 1.528 | 0.006 | 0.041 | 0.031 |
| 1597 | HP_LOWER_LIMB_AMYOTROPHY | 1.536 | 0.006 | 0.041 | 0.031 |
| 1598 | GOBP_ASTROCYTE_ACTIVATION | 1.561 | 0.006 | 0.041 | 0.031 |
| 1599 | GOMF_CYCLASE_ACTIVITY | 1.568 | 0.006 | 0.041 | 0.031 |
| 1600 | GOBP_DENDRITE_DEVELOPMENT | 1.345 | 0.006 | 0.041 | 0.031 |
| 1601 | HP_ABNORMAL_VERTEBRAL_SEGMENTATION_AND_FUSION | 1.576 | 0.006 | 0.041 | 0.031 |
| 1602 | HP_POINTED_CHIN | 1.438 | 0.006 | 0.041 | 0.031 |
| 1603 | GOBP_NEGATIVE_REGULATION_OF_ACUTE_INFLAMMATORY_RESPONSE | 1.554 | 0.006 | 0.041 | 0.031 |
| 1604 | GOBP_RENAL_FILTRATION | 1.579 | 0.006 | 0.041 | 0.031 |
| 1605 | HP_LARGE_EARLOBE | 1.578 | 0.006 | 0.041 | 0.031 |
| 1606 | GOBP_RESPONSE_TO_ISOQUINOLINE_ALKALOID | 1.555 | 0.006 | 0.041 | 0.031 |
| 1607 | HP_MIDDLE_AGE_ONSET | 1.545 | 0.006 | 0.041 | 0.031 |
| 1608 | GOBP_MYOTUBE_CELL_DEVELOPMENT | 1.557 | 0.006 | 0.041 | 0.031 |
| 1609 | GOBP_DEVELOPMENT_OF_PRIMARY_SEXUAL_CHARACTERISTICS | 1.354 | 0.006 | 0.041 | 0.031 |
| 1610 | HP_ABNORMAL_SYNAPTIC_TRANSMISSION_AT_THE_NEUROMUSCULAR_JUNCTION | 1.440 | 0.006 | 0.041 | 0.031 |
| 1611 | GOBP_BIOLOGICAL_PROCESS_INVOLVED_IN_SYMBIOTIC_INTERACTION | 1.336 | 0.006 | 0.041 | 0.031 |
| 1612 | GOBP_REGULATION_OF_HORMONE_SECRETION | 1.344 | 0.006 | 0.041 | 0.031 |
| 1613 | HP_ABNORMALITY_OF_THE_ODONTOID_PROCESS | 1.565 | 0.007 | 0.041 | 0.031 |
| 1614 | GOBP_NEGATIVE_REGULATION_OF_LEUKOCYTE_MEDIATED_IMMUNITY | 1.496 | 0.007 | 0.041 | 0.031 |
| 1615 | GOCC_CELL_CELL_CONTACT_ZONE | 1.494 | 0.007 | 0.041 | 0.031 |
| 1616 | HP_ACHILLES_TENDON_CONTRACTURE | 1.570 | 0.007 | 0.041 | 0.031 |
| 1617 | GOBP_NEGATIVE_REGULATION_OF_OXIDOREDUCTASE_ACTIVITY | 1.544 | 0.007 | 0.042 | 0.031 |
| 1618 | GOBP_NEGATIVE_REGULATION_OF_PRODUCTION_OF_MOLECULAR_MEDIATOR_OF_IMMUNE_RESPONSE | 1.555 | 0.007 | 0.042 | 0.031 |
| 1619 | HP_ABNORMAL_5TH_FINGER_MORPHOLOGY | 1.250 | 0.007 | 0.042 | 0.031 |
| 1620 | GOBP_NEGATIVE_REGULATION_OF_LEUKOCYTE_CHEMOTAXIS | 1.542 | 0.007 | 0.042 | 0.031 |
| 1621 | HP_BUNDLE_BRANCH_BLOCK | 1.535 | 0.007 | 0.042 | 0.031 |
| 1622 | HP_GLOSSOPTOSIS | 1.567 | 0.007 | 0.042 | 0.032 |
| 1623 | HP_PROLONGED_BLEEDING_AFTER_DENTAL_EXTRACTION | 1.569 | 0.007 | 0.042 | 0.032 |
| 1624 | GOBP_REGULATION_OF_SMOOTHENED_SIGNALING_PATHWAY | 1.477 | 0.007 | 0.042 | 0.032 |
| 1625 | GOBP_REGULATION_OF_ENDOCRINE_PROCESS | 1.569 | 0.007 | 0.042 | 0.032 |
| 1626 | GOMF_NUCLEOSIDE_TRIPHOSPHATASE_REGULATOR_ACTIVITY | 1.258 | 0.007 | 0.042 | 0.032 |
| 1627 | GOCC_DYSTROPHIN_ASSOCIATED_GLYCOPROTEIN_COMPLEX | 1.595 | 0.007 | 0.042 | 0.032 |
| 1628 | GOBP_CARDIOBLAST_DIFFERENTIATION | 1.526 | 0.007 | 0.042 | 0.032 |
| 1629 | HP_RHEUMATOID_ARTHRITIS | 1.526 | 0.007 | 0.042 | 0.032 |
| 1630 | GOBP_REGULATION_OF_TYPE_B_PANCREATIC_CELL_PROLIFERATION | 1.551 | 0.007 | 0.042 | 0.032 |
| 1631 | GOBP_RESPONSE_TO_FUNGUS | 1.527 | 0.007 | 0.042 | 0.032 |
| 1632 | GOBP_REGULATION_OF_CAMP_MEDIATED_SIGNALING | 1.537 | 0.007 | 0.042 | 0.032 |
| 1633 | HP_ABNORMAL_NEPHRON_MORPHOLOGY | 1.358 | 0.007 | 0.042 | 0.032 |
| 1634 | GOBP_OUTFLOW_TRACT_MORPHOGENESIS | 1.507 | 0.007 | 0.043 | 0.032 |
| 1635 | GOBP_CELLULAR_RESPONSE_TO_UV_A | 1.572 | 0.007 | 0.043 | 0.032 |
| 1636 | HP_ABNORMALITY_OF_THE_VERTEBRAL_ENDPLATES | 1.573 | 0.007 | 0.043 | 0.032 |
| 1637 | HP_PULMONIC_STENOSIS | 1.418 | 0.007 | 0.043 | 0.032 |
| 1638 | HP_PROLONGED_BLEEDING_AFTER_SURGERY | 1.565 | 0.007 | 0.043 | 0.032 |
| 1639 | GOBP_MONOCYTE_ACTIVATION | 1.564 | 0.007 | 0.043 | 0.032 |
| 1640 | GOMF_CATION_CHANNEL_ACTIVITY | 1.298 | 0.007 | 0.043 | 0.032 |
| 1641 | GOBP_SYNAPSE_PRUNING | 1.572 | 0.007 | 0.043 | 0.032 |
| 1642 | HP_DILATATION_OF_THE_BLADDER | 1.572 | 0.007 | 0.043 | 0.032 |
| 1643 | HP_HIP_DYSPLASIA | 1.369 | 0.007 | 0.043 | 0.032 |
| 1644 | GOBP_APPENDAGE_DEVELOPMENT | 1.385 | 0.007 | 0.043 | 0.032 |
| 1645 | GOBP_PHAGOLYSOSOME_ASSEMBLY | 1.593 | 0.007 | 0.043 | 0.033 |
| 1646 | HP_GENU_RECURVATUM | 1.559 | 0.007 | 0.043 | 0.033 |
| 1647 | GOBP_L_ALPHA_AMINO_ACID_TRANSMEMBRANE_TRANSPORT | 1.528 | 0.007 | 0.044 | 0.033 |
| 1648 | HP_MUSCLE_SPASM | 1.412 | 0.007 | 0.044 | 0.033 |
| 1649 | GOBP_REGULATION_OF_GLIAL_CELL_MIGRATION | 1.562 | 0.007 | 0.044 | 0.033 |
| 1650 | GOBP_REGULATION_OF_NEUROTRANSMITTER_LEVELS | 1.363 | 0.007 | 0.044 | 0.033 |
| 1651 | HP_LIMITED_NECK_RANGE_OF_MOTION | 1.549 | 0.007 | 0.044 | 0.033 |
| 1652 | HP_ABNORMALITY_OF_PERIAURICULAR_REGION | 1.465 | 0.007 | 0.044 | 0.033 |
| 1653 | GOBP_NEGATIVE_REGULATION_OF_POTASSIUM_ION_TRANSPORT | 1.564 | 0.007 | 0.044 | 0.033 |
| 1654 | GOBP_DEFENSE_RESPONSE_TO_GRAM_POSITIVE_BACTERIUM | 1.449 | 0.007 | 0.044 | 0.033 |
| 1655 | GOBP_NEGATIVE_REGULATION_OF_CHONDROCYTE_DIFFERENTIATION | 1.543 | 0.007 | 0.044 | 0.033 |
| 1656 | HP_ABNORMALITY_OF_TIBIA_MORPHOLOGY | 1.438 | 0.007 | 0.044 | 0.033 |
| 1657 | HP_GASTROINTESTINAL_OBSTRUCTION | 1.501 | 0.007 | 0.045 | 0.034 |
| 1658 | GOBP_CELL_ADHESION_MOLECULE_PRODUCTION | 1.553 | 0.007 | 0.045 | 0.034 |
| 1659 | HP_METAMORPHOPSIA | 1.591 | 0.007 | 0.045 | 0.034 |
| 1660 | GOBP_ANATOMICAL_STRUCTURE_MATURATION | 1.331 | 0.007 | 0.045 | 0.034 |
| 1661 | GOCC_ENDOCYTIC_VESICLE_LUMEN | 1.551 | 0.007 | 0.045 | 0.034 |
| 1662 | GOBP_REGULATION_OF_VASCULAR_PERMEABILITY | 1.517 | 0.007 | 0.045 | 0.034 |
| 1663 | HP_APLASIA_HYPOPLASIA_INVOLVING_THE_SKELETAL_MUSCULATURE | 1.517 | 0.007 | 0.045 | 0.034 |
| 1664 | GOBP_REGULATION_OF_LONG_TERM_SYNAPTIC_POTENTIATION | 1.518 | 0.007 | 0.045 | 0.034 |
| 1665 | HP_FOLLICULAR_HYPERKERATOSIS | 1.549 | 0.007 | 0.046 | 0.034 |
| 1666 | GOBP_REGULATORY_T_CELL_DIFFERENTIATION | 1.562 | 0.007 | 0.046 | 0.034 |
| 1667 | HP_RIMMED_VACUOLES | 1.562 | 0.007 | 0.046 | 0.034 |
| 1668 | GOBP_CELL_DIFFERENTIATION_INVOLVED_IN_METANEPHROS_DEVELOPMENT | 1.569 | 0.007 | 0.046 | 0.034 |
| 1669 | HP_INCREASED_MUSCLE_LIPID_CONTENT | 1.547 | 0.007 | 0.046 | 0.034 |
| 1670 | GOBP_REGULATION_OF_JNK_CASCADE | 1.399 | 0.007 | 0.046 | 0.034 |
| 1671 | HP_HYPERLIPIDEMIA | 1.433 | 0.007 | 0.046 | 0.034 |
| 1672 | GOBP_NEGATIVE_REGULATION_OF_RESPONSE_TO_CYTOKINE_STIMULUS | 1.474 | 0.008 | 0.046 | 0.035 |
| 1673 | GOBP_GRANULOCYTE_ACTIVATION | 1.519 | 0.008 | 0.046 | 0.035 |
| 1674 | HP_CHOANAL_STENOSIS | 1.539 | 0.008 | 0.046 | 0.035 |
| 1675 | GOBP_POSITIVE_REGULATION_OF_BONE_MINERALIZATION | 1.525 | 0.008 | 0.046 | 0.035 |
| 1676 | GOBP_POSITIVE_REGULATION_OF_ACTIN_FILAMENT_POLYMERIZATION | 1.529 | 0.008 | 0.046 | 0.035 |
| 1677 | GOBP_POSITIVE_REGULATION_OF_BIOMINERALIZATION | 1.528 | 0.008 | 0.046 | 0.035 |
| 1678 | GOBP_NEGATIVE_REGULATION_OF_POTASSIUM_ION_TRANSMEMBRANE_TRANSPORT | 1.544 | 0.008 | 0.046 | 0.035 |
| 1679 | GOBP_REGULATION_OF_B_CELL_DIFFERENTIATION | 1.546 | 0.008 | 0.046 | 0.035 |
| 1680 | GOBP_NEGATIVE_REGULATION_OF_APOPTOTIC_SIGNALING_PATHWAY | 1.361 | 0.008 | 0.046 | 0.035 |
| 1681 | GOBP_REGULATION_OF_ACTIN_FILAMENT_BUNDLE_ASSEMBLY | 1.455 | 0.008 | 0.046 | 0.035 |
| 1682 | HP_ANOREXIA | 1.444 | 0.008 | 0.046 | 0.035 |
| 1683 | HP_ABNORMAL_TRACHEOBRONCHIAL_MORPHOLOGY | 1.297 | 0.008 | 0.046 | 0.035 |
| 1684 | HP_ABNORMALITY_OF_UPPER_LIMB_METAPHYSIS | 1.588 | 0.008 | 0.046 | 0.035 |
| 1685 | GOBP_CELLULAR_RESPONSE_TO_KETONE | 1.430 | 0.008 | 0.047 | 0.035 |
| 1686 | HP_INABILITY_TO_WALK | 1.340 | 0.008 | 0.047 | 0.035 |
| 1687 | GOCC_DENDRITE_MEMBRANE | 1.523 | 0.008 | 0.047 | 0.035 |
| 1688 | GOCC_INTRINSIC_COMPONENT_OF_POSTSYNAPTIC_DENSITY_MEMBRANE | 1.505 | 0.008 | 0.047 | 0.035 |
| 1689 | GOBP_RETINA_VASCULATURE_MORPHOGENESIS_IN_CAMERA_TYPE_EYE | 1.566 | 0.008 | 0.047 | 0.035 |
| 1690 | GOBP_NEGATIVE_REGULATION_OF_EPITHELIAL_CELL_PROLIFERATION | 1.387 | 0.008 | 0.047 | 0.035 |
| 1691 | GOBP_NEGATIVE_REGULATION_OF_RESPONSE_TO_WOUNDING | 1.461 | 0.008 | 0.047 | 0.035 |
| 1692 | GOBP_POSITIVE_REGULATION_OF_CELL_MATRIX_ADHESION | 1.520 | 0.008 | 0.047 | 0.035 |
| 1693 | GOMF_STRUCTURAL_CONSTITUENT_OF_CYTOSKELETON | 1.444 | 0.008 | 0.047 | 0.035 |
| 1694 | GOBP_POSITIVE_REGULATION_OF_INTERLEUKIN_8_PRODUCTION | 1.518 | 0.008 | 0.047 | 0.035 |
| 1695 | HP_PUNCTATE_CATARACT | 1.541 | 0.008 | 0.047 | 0.035 |
| 1696 | HP_ABNORMALITY_OF_THE_PHALANGES_OF_THE_TOES | 1.413 | 0.008 | 0.047 | 0.035 |
| 1697 | HP_DECREASED_FACIAL_EXPRESSION | 1.402 | 0.008 | 0.047 | 0.035 |
| 1698 | HP_PULMONARY_HYPOPLASIA | 1.415 | 0.008 | 0.047 | 0.035 |
| 1699 | GOBP_REGULATION_OF_CELL_MIGRATION_INVOLVED_IN_SPROUTING_ANGIOGENESIS | 1.488 | 0.008 | 0.047 | 0.035 |
| 1700 | HP_ABNORMAL_FACIAL_EXPRESSION | 1.407 | 0.008 | 0.047 | 0.036 |
| 1701 | GOBP_ENTRY_INTO_HOST | 1.389 | 0.008 | 0.047 | 0.036 |
| 1702 | HP_PERIODONTITIS | 1.559 | 0.008 | 0.047 | 0.036 |
| 1703 | GOBP_ANTIGEN_PROCESSING_AND_PRESENTATION_OF_PEPTIDE_OR_POLYSACCHARIDE_ANTIGEN_VIA_MHC_CLASS_II | 1.537 | 0.008 | 0.048 | 0.036 |
| 1704 | HP_ABNORMALITY_OF_FONTANELLES | 1.370 | 0.008 | 0.048 | 0.036 |
| 1705 | GOBP_NEGATIVE_REGULATION_OF_NITRIC_OXIDE_METABOLIC_PROCESS | 1.535 | 0.008 | 0.048 | 0.036 |
| 1706 | GOBP_POSITIVE_REGULATION_OF_VASCULAR_ASSOCIATED_SMOOTH_MUSCLE_CELL_MIGRATION | 1.535 | 0.008 | 0.048 | 0.036 |
| 1707 | GOBP_REGULATION_OF_DENDRITIC_CELL_CHEMOTAXIS | 1.562 | 0.008 | 0.048 | 0.036 |
| 1708 | GOMF_TAU_PROTEIN_BINDING | 1.520 | 0.008 | 0.049 | 0.037 |
| 1709 | HP_ESOPHAGITIS | 1.523 | 0.008 | 0.049 | 0.037 |
| 1710 | GOBP_AMINO_ACID_TRANSMEMBRANE_TRANSPORT | 1.428 | 0.008 | 0.049 | 0.037 |
| 1711 | GOBP_REGULATION_OF_CARDIAC_EPITHELIAL_TO_MESENCHYMAL_TRANSITION | 1.560 | 0.008 | 0.049 | 0.037 |
| 1712 | GOBP_REGULATION_OF_T_CELL_MEDIATED_IMMUNITY | 1.466 | 0.008 | 0.049 | 0.037 |
| 1713 | GOBP_POSITIVE_REGULATION_OF_STRESS_FIBER_ASSEMBLY | 1.509 | 0.008 | 0.049 | 0.037 |
| 1714 | GOBP_ACUTE_INFLAMMATORY_RESPONSE_TO_ANTIGENIC_STIMULUS | 1.562 | 0.008 | 0.049 | 0.037 |
| 1715 | GOBP_NEGATIVE_REGULATION_OF_CATION_CHANNEL_ACTIVITY | 1.497 | 0.008 | 0.050 | 0.037 |
| 1716 | HP_CORNEAL_DYSTROPHY | 1.558 | 0.008 | 0.050 | 0.037 |
| 1717 | HP_TOE_WALKING | 1.509 | 0.008 | 0.050 | 0.037 |
| 1718 | GOBP_ORGANELLE_MEMBRANE_FUSION | 1.426 | 0.008 | 0.050 | 0.037 |
| 1719 | HP_ABNORMALITY_OF_THE_AMNIOTIC_FLUID | 1.281 | 0.008 | 0.050 | 0.038 |

**2.5 Supplementary Table 5.** 51 KEGG pathways enriched by GSEA.

| **NO.** | **ID** | **NES** | **P value** | **P.adjust** | **Qvalues** |
| --- | --- | --- | --- | --- | --- |
| 1 | KEGG_CHEMOKINE_SIGNALING_PATHWAY | 1.836 | <0.001 | <0.001 | <0.001 |
| 2 | KEGG_CYTOKINE_CYTOKINE_RECEPTOR_INTERACTION | 1.863 | <0.001 | <0.001 | <0.001 |
| 3 | KEGG_FOCAL_ADHESION | 1.843 | <0.001 | <0.001 | <0.001 |
| 4 | KEGG_SYSTEMIC_LUPUS_ERYTHEMATOSUS | 2.017 | <0.001 | <0.001 | <0.001 |
| 5 | KEGG_HEMATOPOIETIC_CELL_LINEAGE | 1.939 | <0.001 | <0.001 | <0.001 |
| 6 | KEGG_CELL_ADHESION_MOLECULES_CAMS | 1.808 | <0.001 | <0.001 | <0.001 |
| 7 | KEGG_COMPLEMENT_AND_COAGULATION_CASCADES | 1.912 | <0.001 | <0.001 | <0.001 |
| 8 | KEGG_METABOLISM_OF_XENOBIOTICS_BY_CYTOCHROME_P450 | -2.158 | <0.001 | <0.001 | <0.001 |
| 9 | KEGG_ECM_RECEPTOR_INTERACTION | 1.836 | <0.001 | <0.001 | <0.001 |
| 10 | KEGG_NEUROACTIVE_LIGAND_RECEPTOR_INTERACTION | 1.607 | <0.001 | <0.001 | <0.001 |
| 11 | KEGG_VASCULAR_SMOOTH_MUSCLE_CONTRACTION | 1.684 | <0.001 | <0.001 | <0.001 |
| 12 | KEGG_REGULATION_OF_ACTIN_CYTOSKELETON | 1.590 | <0.001 | <0.001 | <0.001 |
| 13 | KEGG_AUTOIMMUNE_THYROID_DISEASE | 1.806 | <0.001 | <0.001 | <0.001 |
| 14 | KEGG_RIBOSOME | -1.879 | <0.001 | <0.001 | <0.001 |
| 15 | KEGG_LEUKOCYTE_TRANSENDOTHELIAL_MIGRATION | 1.665 | <0.001 | <0.001 | <0.001 |
| 16 | KEGG_ASTHMA | 1.782 | <0.001 | <0.001 | <0.001 |
| 17 | KEGG_DILATED_CARDIOMYOPATHY | 1.734 | <0.001 | <0.001 | <0.001 |
| 18 | KEGG_VIRAL_MYOCARDITIS | 1.785 | <0.001 | <0.001 | <0.001 |
| 19 | KEGG_HYPERTROPHIC_CARDIOMYOPATHY_HCM | 1.714 | <0.001 | <0.001 | <0.001 |
| 20 | KEGG_CALCIUM_SIGNALING_PATHWAY | 1.603 | <0.001 | <0.001 | <0.001 |
| 21 | KEGG_DRUG_METABOLISM_CYTOCHROME_P450 | -1.840 | <0.001 | 0.001 | <0.001 |
| 22 | KEGG_INTESTINAL_IMMUNE_NETWORK_FOR_IGA_PRODUCTION | 1.732 | <0.001 | 0.001 | <0.001 |
| 23 | KEGG_LEISHMANIA_INFECTION | 1.719 | <0.001 | 0.001 | 0.001 |
| 24 | KEGG_ALLOGRAFT_REJECTION | 1.704 | <0.001 | 0.001 | 0.001 |
| 25 | KEGG_PENTOSE_AND_GLUCURONATE_INTERCONVERSIONS | -1.950 | <0.001 | 0.002 | 0.001 |
| 26 | KEGG_TOLL_LIKE_RECEPTOR_SIGNALING_PATHWAY | 1.575 | <0.001 | 0.003 | 0.002 |
| 27 | KEGG_PRIMARY_IMMUNODEFICIENCY | 1.654 | <0.001 | 0.003 | 0.002 |
| 28 | KEGG_STEROID_HORMONE_BIOSYNTHESIS | -1.767 | <0.001 | 0.003 | 0.002 |
| 29 | KEGG_GAP_JUNCTION | 1.596 | 0.001 | 0.003 | 0.002 |
| 30 | KEGG_MELANOMA | 1.620 | 0.001 | 0.005 | 0.003 |
| 31 | KEGG_JAK_STAT_SIGNALING_PATHWAY | 1.502 | 0.001 | 0.005 | 0.004 |
| 32 | KEGG_RETINOL_METABOLISM | -1.690 | 0.001 | 0.006 | 0.004 |
| 33 | KEGG_PRION_DISEASES | 1.612 | 0.001 | 0.007 | 0.005 |
| 34 | KEGG_ARRHYTHMOGENIC_RIGHT_VENTRICULAR_CARDIOMYOPATHY_ARVC | 1.585 | 0.001 | 0.007 | 0.005 |
| 35 | KEGG_MAPK_SIGNALING_PATHWAY | 1.375 | 0.001 | 0.007 | 0.005 |
| 36 | KEGG_PORPHYRIN_AND_CHLOROPHYLL_METABOLISM | -1.708 | 0.002 | 0.009 | 0.007 |
| 37 | KEGG_GRAFT_VERSUS_HOST_DISEASE | 1.597 | 0.002 | 0.010 | 0.007 |
| 38 | KEGG_PATHWAYS_IN_CANCER | 1.357 | 0.002 | 0.011 | 0.008 |
| 39 | KEGG_TGF_BETA_SIGNALING_PATHWAY | 1.516 | 0.002 | 0.011 | 0.008 |
| 40 | KEGG_NATURAL_KILLER_CELL_MEDIATED_CYTOTOXICITY | 1.445 | 0.003 | 0.015 | 0.011 |
| 41 | KEGG_NOD_LIKE_RECEPTOR_SIGNALING_PATHWAY | 1.531 | 0.005 | 0.024 | 0.017 |
| 42 | KEGG_LINOLEIC_ACID_METABOLISM | -1.627 | 0.006 | 0.028 | 0.020 |
| 43 | KEGG_TYPE_I_DIABETES_MELLITUS | 1.515 | 0.007 | 0.028 | 0.020 |
| 44 | KEGG_WNT_SIGNALING_PATHWAY | 1.415 | 0.007 | 0.028 | 0.020 |
| 45 | KEGG_PEROXISOME | -1.508 | 0.007 | 0.031 | 0.022 |
| 46 | KEGG_T_CELL_RECEPTOR_SIGNALING_PATHWAY | 1.392 | 0.009 | 0.036 | 0.026 |
| 47 | KEGG_ANTIGEN_PROCESSING_AND_PRESENTATION | 1.473 | 0.010 | 0.038 | 0.027 |
| 48 | KEGG_B_CELL_RECEPTOR_SIGNALING_PATHWAY | 1.472 | 0.010 | 0.039 | 0.027 |
| 49 | KEGG_DRUG_METABOLISM_OTHER_ENZYMES | -1.479 | 0.012 | 0.044 | 0.031 |
| 50 | KEGG_RENIN_ANGIOTENSIN_SYSTEM | 1.533 | 0.012 | 0.044 | 0.031 |
| 51 | KEGG_PROGESTERONE_MEDIATED_OOCYTE_MATURATION | 1.420 | 0.012 | 0.045 | 0.032 |
